# Highly Conductive and Stable Naphthalenediimide-Based Organic Salt Cathode for Robust Lithium-Ion Batteries

**DOI:** 10.1007/s40820-026-02176-x

**Published:** 2026-04-08

**Authors:** Xiangyu Su, Zixuan Shan, Xuan Peng, Jianyi Chu, Zhihao Jia, Yanan Kou, Min Jiang, Yuan Chen

**Affiliations:** 1 College of Energy Materials and Chemistry, State Key Laboratory of New Textile Materials and Advanced Processing, Inner Mongolia University, Hohhot, 010070 People’s Republic of China; 2 Department of Physics, City University of Hong Kong, Hong Kong, 999077 People’s Republic of China; 3 School of Materials Science and Engineering, Shanghai Jiao Tong University, Shanghai, 200240 People’s Republic of China

**Keywords:** Organic electrodes, Organic lithium salt cathode, Naphthalenediimide, Lithium-ion batteries, *π*-conjugated structure

## Abstract

Organic lithium salts are promising cathode candidates for sustainable lithium-ion batteries (LIBs) due to their synthetic simplicity and environmental friendliness. However, their practical application is often thwarted by active material dissolution and sluggish redox kinetics, stemming from limited conjugation and deficient electronic conductivity. Herein, we report a naphthalenediimide-based lithium salt (NDI-OLi) designed with a robust π-conjugated structure and high aromaticity to enhance electronic transport. Experimental and theoretical studies reveal that the relatively high electronic conductivity, rapid reaction kinetics, and stable delocalized electronic geometry of the NDI-OLi cathode are key factors enabling sufficient coordination between carbonyl sites and lithium ions. As a result, the NDI-OLi electrode presents a high reversible capacity of 160 mAh g^−1^ at 0.1 A g^−1^, excellent rate performance (99.9 mAh g^−1^ at 8 A g^−1^), and exceptional cycling stability with 85% capacity retention after 5000 cycles at 1 A g^−1^. This work opens up new avenues for developing highly conductive and stable organic salt cathodes toward high-performance and durable LIBs.

**Supplementary Information:**

The online version contains supplementary material available at 10.1007/s40820-026-02176-x.

## Highlights

- A naphthalenediimide-based lithium salt (NDI-OLi) featuring a stable π-conjugated structure and high aromaticity is proposed as a cathode, which facilitates relatively high electronic conductivity and rapid electron transfer.
- Experimental and theoretical investigations reveal that the stable delocalized electronic geometry and fast reaction kinetics enable sufficient coupling between carbonyl sites and lithium ions, ensuring excellent energy storage performance.
- The NDI-OLi electrode demonstrates superior rate capability and long-term cyclability (85% capacity retention after 5000 cycles), outperforming previously reported organic lithium salt cathodes.

**Supplementary Information:**

The online version contains supplementary material available at 10.1007/s40820-026-02176-x.

## Graphical abstract

**Figure:**
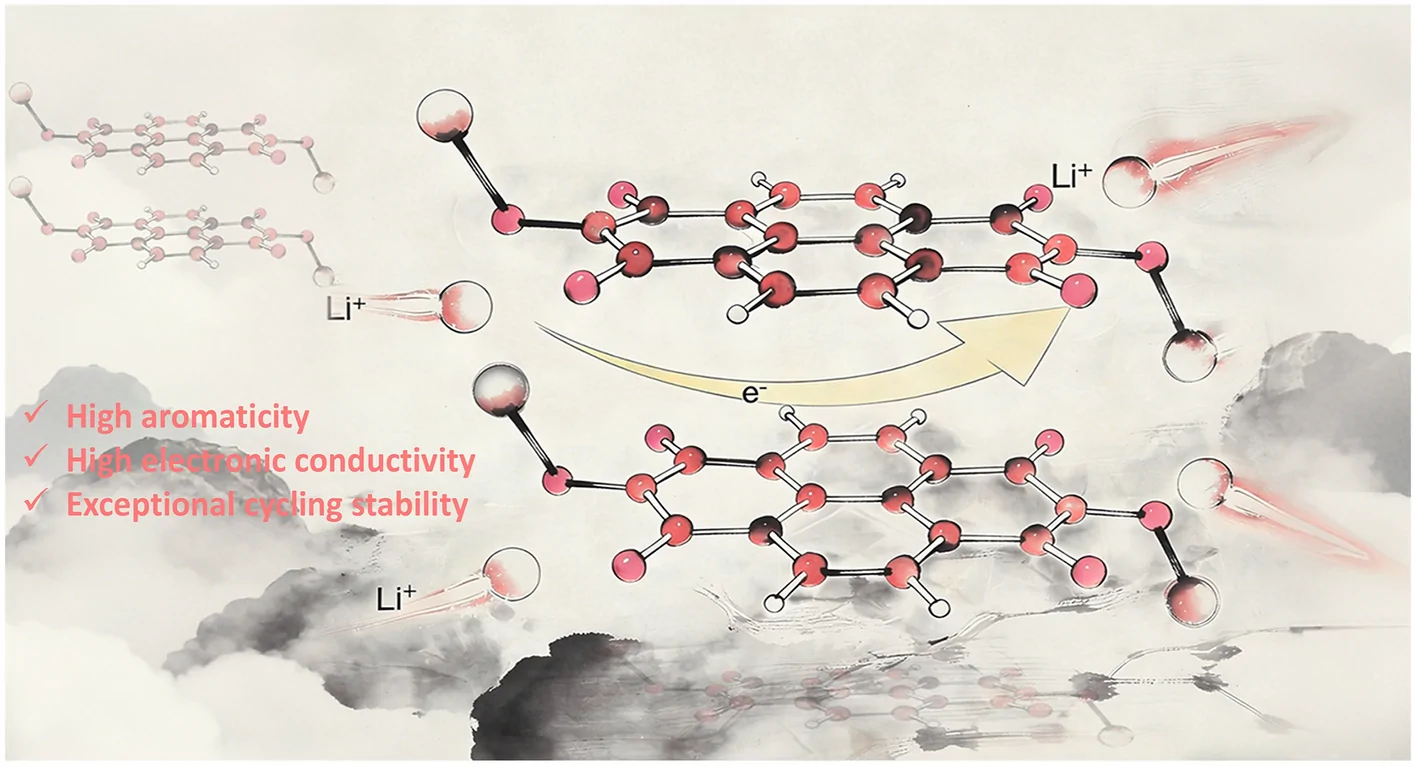

## Introduction

Over the past few decades, lithium-ion batteries (LIBs) have achieved widespread application across multiple sectors, successfully driving the advancement of key industries such as portable electronics, grid energy storage systems, and electric vehicles [1–5]. However, inorganic cathode materials employed in commercial lithium-ion batteries face challenges such as limited resource availability, high energy consumption during preparation and recycling, and severe environmental pollution [6, 7]. This has stimulated a surge in research interest in organic electrode materials, which offer the advantages of low cost, abundant sources, structural flexibility, and environmental friendliness [8–12]. In addition, organic electrode materials possess the potential to achieve satisfactory electrochemical performances with high capacity and adjustable voltage due to their structural versatility and designability [13–23].

Among numerous organic electrode materials, carbonyl compounds have garnered increasing attention as potential cathode materials for LIBs due to their excellent redox activity and high discharge operating voltage (~ 2.5 V) [24]. However, the high solubility of small-molecule carbonyl compounds in electrolytes severely restricts their application development. Although polymerization can mitigate this issue, it typically results in reduced energy density due to the presence of redox-inactive linkers and involves complex synthetic procedures [25–31]. In contrast, the salification strategy for small molecules offers simpler synthesis and higher efficiency, the O···M···O ionic/coordination bonds formed between molecules can significantly strengthen intermolecular interactions [32]. Despite achieving breakthroughs in capacity with quinone-based salts, maintaining long-term cycling stability remains elusive [33–35]. Some studies indicate that although ionic bonds enhance intermolecular interactions, the insertion/extraction of metal ions during cycling still causes phase transitions, particle fragmentation and dissolution, all of which are related to molecular structural stability [36]. More importantly, such small-molecule salts inherently possess low conductivity, typically requiring the addition of at least 30% or even 40% conductive carbon additives and then significantly limiting the battery’s energy density and fast-charging performance [27, 37–44]. Therefore, there is an urgent need to develop novel organic salt cathodes that simultaneously possess high conductivity and superior structural stability to enable high-rate and durable LIBs.

**Fig. 1:**
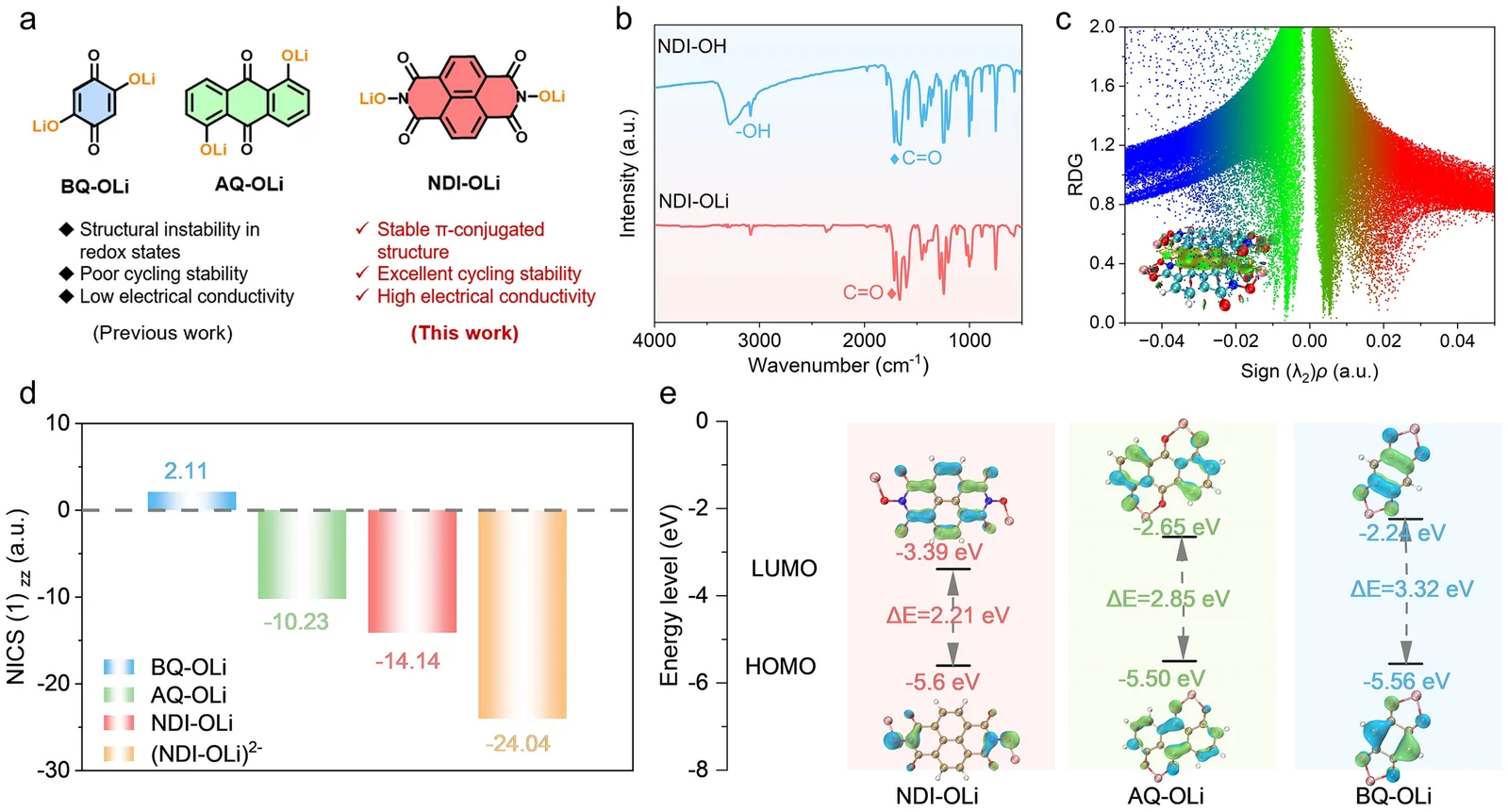
**a** Structural formula and respective advantages and disadvantages of NDI-OLi, AQ-OLi and BQ-OLi. **b** The FTIR spectra of NDI-OH and NDI-OLi. **c** Scatter diagram of RDG versus sign (*λ*_2_)ρ (inset is the corresponding gradient isosurface). **d** Calculated NICS(1)_zz_ values of BQ-OLi, AQ-OLi, NDI-OLi and (NDI-OLi)^2−^. **e** HOMO and LUMO energy levels for NDI-OLi, BQ-OLi and AQ-OLi, respectively

Herein, we report a naphthalenediimide-based lithium salt (NDI-OLi) with stable *π*-conjugated structure as a cathode for LIBs (Fig. 1a). The stable *π*-conjugated structure and strong intermolecular *π*-*π* interactions endow NDI-OLi with relatively high conductivity, facilitating rapid electron transfer. Compared with control samples lithium 2,5-dihydroxybenzoquinone (BQ-OLi) and lithium 1,5-dihydroxy-9,10-anthraquinone (AQ-OLi), NDI-OLi exhibits higher aromaticity and structural stability. Consequently, the NDI-OLi cathode delivers a high specific capacity of 160 mAh g^−1^ at 0.1 A g^−1^ (average operating voltage 2.6 V), exhibits outstanding rate performance (99.9 mAh g^−1^ capacity retention at 8 A g^−1^), and maintains 85% capacity retention after 5000 cycles at 1 A g^−1^, significantly surpassing the electrochemical performance of BQ-OLi and AQ-OLi. Additionally, the NDI-OLi//graphite full battery also shows a high discharge capacity of 136.7 mAh g^−1^ and excellent cycling stability (95% capacity retention after 1000 cycles at 1 A g^−1^). Furthermore, the storage mechanism of NDI-OLi is comprehensively revealed through in situ characterization and theoretical calculations. This work provides a promising strategy for designing highly conductive and stable organic salt cathodes for advanced LIBs.

## Experimental Section

### Material

MeOH, N,N-dimethylformamide (DMF), Et_3_N, hydroxyl amine hydrochloride and lithium methoxide were purchased from Energy Chemical. Lithium hydroxide was purchased from InnoChem. 1,4,5,8-Naphthalenetetracarboxylic dianhydride was purchased from Tansoole. 2,6-Dihydroxy-anthraquinon and 2,5-dihydroxy-1,4-benzoquinone were purchased from Bide Pharmatech Ltd. Poly(vinylidene fluoride) (PVDF) and sodium carboxymethyl cellulose (CMC-Na), 1 M LiTFSI/DME:DOL were purchased from DoDoChem. All chemical reagents and solvents are commercially available and require no additional purification.

### Synthesis of N,N’-Dihydroxy Naphthalenediimide (NDI-OH)

NDI-OH was synthesized according to previous literature [45]. 1,4,5,8-Naphthalenetetracarboxylic dianhydride (NDI, 5.4 g, 20 mmol) and hydroxyl amine hydrochloride (4.62 g, 60 mmol) were dispersed into 100 mL of anhydrous DMF. The mixture was stirred at 70 °C for 3 h and then refluxed for another 5 h. The yellow solid was obtained by thermal filtration and then washed with a small amount of DMF and diethyl ether. ^1^H-NMR (600 MHz, d_6_-DMSO): d(ppm) 11.02 (s, 2H), 8.69 (s, 4H).

### Synthesis of NDI-OLi

The compound NDI-OLi was synthesized by a simple deprotonation process. 0.6 g of NDI-OH was dissolved in 30 mL of anhydrous methanol, followed by the addition of 0.27 g of lithium methoxide. The mixture was stirred at room temperature for 24h, the product was filtered and then washed several times sequentially with anhydrous methanol and diethyl ether. The obtained brown powder (NDI-OLi) was dried in a vacuum at 80 °C.

### Synthesis of AQ-OLi

0.56 g of AQ-OH (4 mmol) was dissolved in 50 mL of deionized water and 0.22 g of lithium hydroxide (9 mmol) was added. The reaction mixture was stirred at 80 °C for 12 h to ensure complete deprotonation. The resulting product was purified through sequential washing with deionized water and absolute ethanol to remove residual reactants and byproducts. Finally, the obtained yellow–brown powder was vacuum-dried at 80 °C for 24 h.

### Synthesis of BQ-OLi

The synthesis of BQ-OLi was similar to the preparation of AQ-OLi. Specifically, 0.48 g (2 mmol) of BQ-OH was dissolved in 30 mL of deionized water. After addition of 0.12 g (5 mmol) of lithium hydroxide, the reaction mixture was stirred vigorously at 80 °C for 12 h under nitrogen. The resulting product was purified by multiple washes with deionized water and absolute ethanol to remove unreacted starting materials and lithium salts. Vacuum drying at 80 °C for 24 h resulted in an orange crystalline product.

## Results and Discussion

### Synthesis and Characterization

The three organic lithium salts BQ-OLi, AQ-OLi, and NDI-OLi were synthesized via a simple deprotonation process, with the detailed synthetic routes shown in Fig. S1. The chemical structures of three samples were characterized using Fourier transform infrared (FTIR) spectroscopy. As shown in Fig. 1b, the characteristic C=O signal at 1700 cm^−1^ remains observable in the NDI-OLi, while the broad absorption band associated with the -OH groups (at ~ 3300 cm^−1^) completely disappears, indicating successful proton elimination [46, 47]. Similar structural transitions were observed for BQ-OLi and AQ-OLi (Fig. S2). In addition, powder X-ray diffraction (PXRD) results indicate that NDI-OLi exhibits sharper and more intense diffraction peaks compared to its precursor NDI-OH (Fig. S3), indicating significantly enhanced crystallinity following salification. Notably, NDI-OLi possesses higher crystallinity than both BQ-OLi and AQ-OLi. This superior crystalline order is likely attributed to the expanded *π*-conjugated system and enhanced molecular planarity, which facilitate stronger intermolecular *π*-*π* stacking [35]. Scanning electron microscope (SEM) images show that all three samples exhibit bulk particle morphology (Fig. S4). Furthermore, thermogravimetric analysis (TGA) indicates that salification substantially improves the thermal stability of these molecules (Fig. S5). The decomposition temperature of AQ-OLi is higher than that of NDI-OLi, which may be ascribed to the thermal susceptibility of the imide rings.

To investigate the dissolution behavior of materials, the UV–Vis absorption spectroscopy analysis was conducted. As shown in Fig. S6, NDI-OLi powder exhibits no distinct absorption peaks compared to the control samples, indicating lower solubility. We further investigated the dissolution behavior of the NDI-OLi electrode in the electrolyte. Even after immersion for up to 48 h, the NDI-OLi electrode shows no clear characteristic absorption peaks (Fig. S7). In parallel, gas chromatography (GC) results further confirmed that the NDI-OLi electrode is insoluble in the electrolyte (Fig. S8), demonstrating excellent dissolution resistance. This property helps maintain the structural stability of the electrode, thereby enhancing the cycling performance of the battery. The dissolution suppression effect is likely attributed to the more stable molecular structure of NDI-OLi and its strong intermolecular interactions. This interaction was visualized through reduced density gradient (RDG) analysis and the corresponding gradient isosurface (Fig. 1c). The green peak detected in the region where sign(*λ*_2_)ρ ranges from − 0.02–0 atomic units clearly identifies *π*-*π* interactions between NDI-OLi molecules. Furthermore, the aromaticity and electronic stability of the salts were further quantified using the iso-chemical shielding surface (ICSS) method [48]. As depicted in Figs. 1d and S9, the central naphthalene ring of NDI-OLi exhibits negative NICS(1)_zz_ values, indicating its aromatic character. The diimide rings are non-aromatic (NICS(1)_zz_ values are positive). The quinone ring of BQ-OLi exhibits a positive value, while the total NICS(1)_zz_ value for AQ-OLi is also more positive than that of NDI-OLi. Evidently, the total NICS(1)_zz_ values indicate an aromaticity sequence of NDI-OLi > AQ-OLi > BQ-OLi, consistent with their molecular stability. Notably, the NICS(1)zz values for (NDI-OLi)^2−^ become even more negative upon reduction (Fig. S9), signifying that the entire framework achieves enhanced aromatic delocalization in its reduced state, thereby stabilizing the lithiated discharge products.

Based on molecular orbital theory, highly conjugated structures can reduce the energy levels (Figs. 1e and S10) between the lowest unoccupied molecular orbital (LUMO) and the highest occupied molecular orbital (HOMO). As shown in Fig. 1e, the energy gap of NDI-OLi (2.21 eV) is significantly narrower than that of BQ-OLi (3.32 eV) and AQ-OLi (2.85 eV), implying a lower barrier for electron excitation and enhanced thermodynamic stability. Additionally, the lower LUMO energy corresponds to higher electron affinity, contributing to improved reduction potential. The LUMO electron density of NDI-OLi is distributed throughout the molecular structure, exhibiting the lowest LUMO energy (− 3.39 eV), while BQ-OLi and AQ-OLi have LUMO energies of − 2.24 and − 2.65 eV, respectively. Local orbital localization plot (LOL-*π*) further confirms the highly conjugated structure and delocalized electrons throughout the NDI-OLi molecule (Fig. S11). This enhanced charge delocalization along the conjugated structure facilitates electron injection into the organic core. Furthermore, NDI-OLi exhibits a low optical bandgap (*E*_*g*_) of 1.34 eV (Fig. S12), ensuring relatively high intrinsic electronic conductivity. The electronic conductivity of the three materials was further measured using the four-probe method, where the conductivity of NDI-OLi, AQ-OLi and BQ-OLi powder was 9.06 × 10^−7^, 1.47 × 10^−7^, and 8.62 × 10^−10^ S m^−1^, respectively (Table S1). The conductivity of NDI-OLi powder is higher than that of most reported organic materials (Table S2). This relatively high conductivity effectively promotes electron transfer at the electrode/electrolyte interface, thereby enhancing the utilization of active sites in the NDI-OLi cathode.

### Electrochemical Performance

Considering the water-soluble nature of the organic lithium salt products, water-soluble sodium carboxymethyl cellulose (CMC) and hydrophobic fluorinated polyvinylidene fluoride (PVDF) binders were evaluated to optimize the electrochemical performance. As shown in Figs. S13-S18, the electrode using CMC (NDI‑OLi(CMC)) exhibits significantly better performance in capacity, cycling stability, and rate capability compared to the electrode using PVDF (NDI‑OLi(PVDF)). For example, NDI‑OLi(CMC) delivers a discharge capacity of 163 mAh g^−1^ at 100 mA g^−1^, while NDI‑OLi(PVDF) only reaches 97 mAh g^−1^ (Fig. S13). After 1000 cycles at 1 A g^−1^, the capacity of NDI‑OLi(CMC) shows no significant capacity decay (Fig. S14), whereas that of NDI‑OLi(PVDF) retains only 68%. The difference in electrochemical performance between the two binders is likely attributed to the dissolution–reprecipitation process of NDI‑OLi in aqueous solution during electrode preparation with the water‑soluble CMC binder. This process promotes uniform dispersion of active material particles and intimate contact with the conductive network. SEM images (Fig. S15) confirm that the NDI‑OLi(CMC) electrode exhibits a more homogeneous particle distribution before cycling. In contrast, noticeable agglomeration is observed in the NDI‑OLi(PVDF) electrode. These morphological differences highlight the excellent compatibility between NDI‑OLi and CMC, which benefits subsequent charge–discharge processes. Electrochemical impedance spectroscopy (EIS) further reveals that under identical conditions, the charge transfer resistance of NDI‑OLi(CMC) is significantly lower than that of NDI‑OLi(PVDF). Specifically, after 20 cycles, the impedance of NDI‑OLi(CMC) increases only from 30 to 60 Ω, whereas that of NDI‑OLi(PVDF) rises sharply from 40 to 200 Ω, demonstrating superior interfacial charge transfer kinetics for the CMC‑based electrode (Fig. S16). Moreover, TEM results show that the SEI layer on the NDI‑OLi(CMC) electrode is thinner and more uniform (Fig. S17). This stable SEI suppresses continuous side reactions and facilitates reversible lithium‑ion insertion/extraction. Furthermore, XPS spectra of the NDI‑OLi(PVDF) electrode show a significant and irreversible decrease in the fluorine signal after cycling (Fig. S18), particularly relative to other elements such as C and N. This defluorination weakens the adhesion of the binder, leading to particle agglomeration, detachment, increased impedance, and eventual performance degradation [49], which is consistent with the morphological changes observed in PVDF‑based electrodes after cycling. Although the binder also affects the electrochemical performance of AQ‑OLi and BQ‑OLi, both materials exhibit pronounced capacity decay with either CMC or PVDF due to the dissolution and instability of intermediate products (Figs. S19 and S20). These findings highlight the superior compatibility between NDI-OLi and CMC. Consequently, CMC is employed as the binder in all subsequent systematic evaluations of the three materials.

**Fig. 2:**
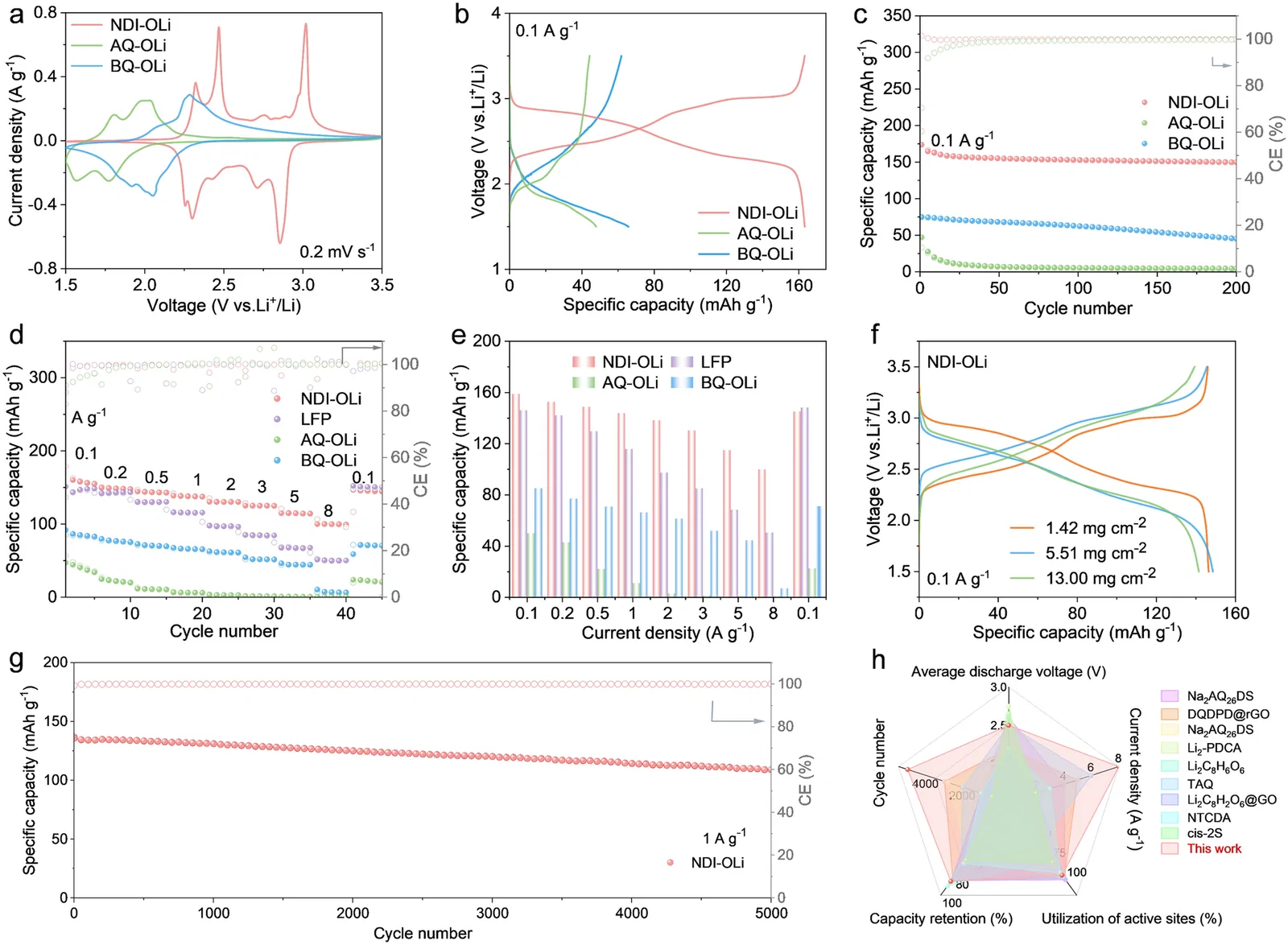
**a** CV curves at 0.2 mV s^−1^, **b** charge–discharge profile at 0.1 A g^−1^, and **c** cycling performance at 0.1 A g^−1^ of NDI-OLi (the mass of the active material is 1.2 mg cm^−2^), AQ-OLi and BQ-OLi, respectively. **d**, **e** Rate capability of NDI-OLi (the mass of the active material is 1.1 mg cm^−2^), AQ-OLi, BQ-OLi and LFP, respectively. **f** The charge–discharge curves with different mass loading and **g** long-term cycling stability of NDI-OLi (the mass of the active material is 1.26 mg cm^−2^). **h** Comparison of the electrochemical performance of NDI-OLi with representative organic lithium salt cathodes

As shown in Fig. 2a, the cyclic voltammetry (CV) curves of three organic salt cathodes (NDI-OLi, AQ-OLi and BQ-OLi) at 0.2 mV s^−1^ show significant differences. The NDI-OLi electrode shows four pairs of distinct redox peaks at 2.25/2.32, 2.30/2.47, 2.71/2.75, and 2.86/3.02 V, with an average discharge voltage of 2.6 V. In contrast, the average discharge voltages of AQ-OLi and BQ-OLi are 1.8 and 1.9 V, respectively. Notably, the CV curve integral area of NDI-OLi is significantly larger than that of other materials, directly reflecting its higher capacity. As shown in Figs. S21 and S22, the NDI-OLi electrode achieves initial charge–discharge capacities of 192/174 mAh g^−1^ at a current density of 0.1 A g^−1^, with an initial coulombic efficiency (ICE) of 90.6%, performing better than AQ-OLi (83.9%) and BQ-OLi (87.1%). The higher ICE values generally indicate fewer side reactions at the electrode/electrolyte interface [49, 50], which contributes to improved cycling stability and reversible capacity of the electrodes. In addition, during the first three cycles, NDI-OLi shows low capacity decay, indicating good electrochemical reversibility (Fig. S21). As shown in Fig. 2b, NDI-OLi delivers a high discharge capacity of 163 mAh g^−1^ at 0.1 A g^−1^ (94% of the theoretical capacity, corresponding to two-electron transfer). In contrast, BQ-OLi exhibits a capacity of approximately 70 mAh g^−1^, while AQ-OLi only reaches about 50 mAh g^−1^. The specific capacity of NDI-OLi is nearly twice and three times higher than those of BQ-OLi and AQ-OLi, respectively. Impressively, the NDI-OLi electrode demonstrates outstanding stability with 92% capacity retention after 200 cycles at 0.1 A g^−1^ (Fig. 2c). In contrast, BQ-OLi and AQ-OLi exhibit capacity decline early in cycling, with AQ-OLi nearly completely depleted after fewer than 50 cycles. This low capacity stems from their inherently low conductivity and molecular structural instability, leading to material dissolution during cycling. The combination of relatively high conductivity and stable molecular structure enhanced the material’s electron transfer kinetics, thereby improving the utilization efficiency of redox-active groups in NDI-OLi and ultimately achieving superior capacity. Consequently, the NDI-OLi electrode achieves impressive cycle stability even at an elevated current density of 1 A g^−1^, delivering a capacity of 110 mAh g^−1^ (89.4 μAh cm^−2^) with 85% capacity retention after 5000 cycles (Figs. 2g and S23). In addition, NDI‑OLi maintains a reversible capacity of 72 mAh g^−1^ after 2000 cycles at 8 A g^−1^, indicating its fast kinetics and excellent cycle stability (Fig. S24). To further elucidate the mechanisms underlying the exceptional cycling stability of NDI-OLi, the cells were disassembled after 1000 cycles for detailed investigation. As shown in Fig. S25, the separator remained colorless, initially suggesting minimal loss of active material. Furthermore, the cycled electrodes were immersed in the electrolyte for 48 h, during which no discernible color change was observed, confirming the low solubility of NDI-OLi. The electrolyte soaked with the electrode was further analyzed by UV–Vis spectroscopy. As shown in Fig. S26, the absorbance peaks of the soaking solutions at various time intervals remain very weak, which fully demonstrates the excellent dissolution suppression capability of NDI-OLi and ensures its outstanding cycling stability.

The rate performances of three electrodes were also investigated at different current densities. As shown in Figs. 2d, e and S27, the NDI-OLi electrode exhibits excellent rate performance, much better than the other electrodes. At current densities of 0.1, 0.2, 0.5, 1, 2, 3, 5, and 8 A g^−1^, the electrode delivers capacities of 158.9, 152.6, 148.8, 143.8, 138.2, 130.3, 114.8, and 99.9 mAh g^−1^ (the corresponding areal capacities are 113.6, 109.1, 106.4, 102.8, 98.8, 93.2, 82.1, and 71.4 μAh cm^−2^), respectively, fully demonstrating its fast kinetics. In contrast, the BQ-OLi and AQ-OLi cathodes not only exhibit low capacity at 0.1 A g^−1^ but also show near-complete capacity decay at 8 A g^−1^, indicating sluggish redox kinetics. Remarkably, NDI-OLi exhibits particularly outstanding high-rate performance even when compared to commercial lithium iron phosphate (LFP) cathodes. Even at 8 A g^−1^, it maintains a capacity of 99.9 mAh g^−1^, far surpassing LFP materials. These findings highlight the great potential of rationally designed organic cathode materials to overcome the limitations of traditional inorganic battery materials in fast charging/discharging applications. Furthermore, the low-temperature electrochemical performance of NDI-OLi was systematically evaluated. As shown in Fig. S28, at a current density of 0.1 A g^−1^, NDI-OLi delivers reversible capacities of 119.2 mAh g^−1^ (78% of the room-temperature capacity) and 99.6 mAh g^−1^ (65% of the room-temperature capacity) at 0 and − 10 °C, respectively. Notably, even at − 10 °C and a high current density of 1 A g^−1^, the NDI-OLi electrode maintains a capacity of 78.3 mAh g^−1^ after 500 cycles, corresponding to an outstanding capacity retention of 97%. These findings underscore the rapid charge transfer kinetics and superior structural robustness of NDI-OLi under low-temperature operating conditions.

Benefiting from the stable conjugated structure and relatively high conductivity of NDI-OLi, it maintains high capacity under high mass-loading conditions (Figs. 2f and S29). At 0.1 A g^−1^, the capacity reaches 148.6 mAh g^−1^ with a loading of 5.51 mg cm^−2^, showing negligible capacity decay after 40 cycles. Even when the loading increases to 13 mg cm^−2^, the capacity remains at 141.5 mAh g^−1^. In terms of both cycling and rate performance, the NDI-OLi electrode outperformed all previously reported organic lithium salt cathodes (Table S3 and Fig. 2h). To explore the practical feasibility of NDI-OLi, pouch cell with dimensions of 2.5 cm × 2.5 cm was fabricated. The cell delivers a specific capacity of approximately 120 mAh g^−1^ at 0.05 A g^−1^ with stable cycling over 40 cycles (Fig. S30). Moreover, the pouch cell can illuminate a device composed of 34 parallel‑connected LEDs even under bending (Fig. S31), demonstrating its potential for use in flexible and wearable electronic fields.

**Fig. 3:**
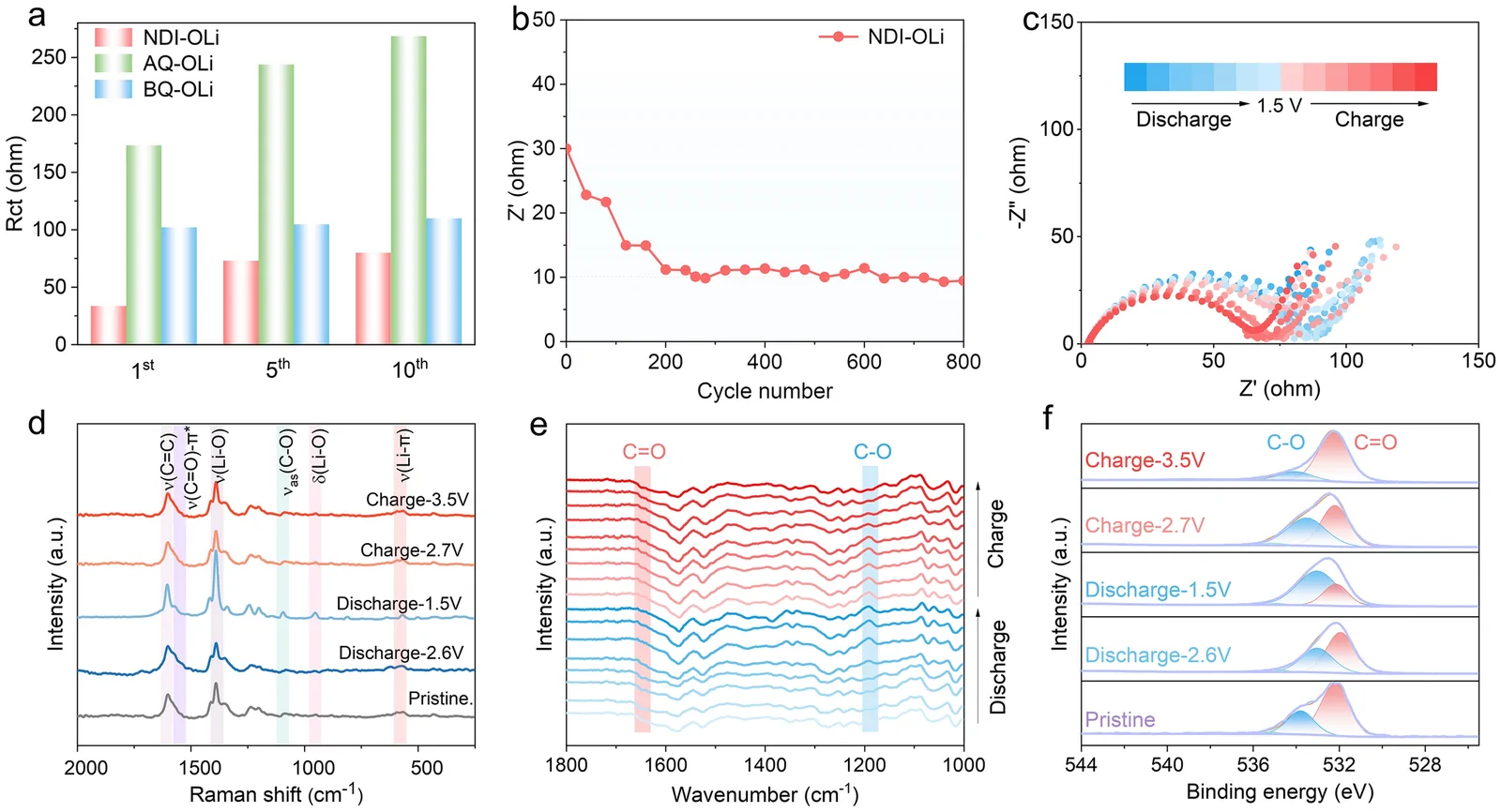
**a** Bar charts of ex situ resistance values for NDI-OLi, AQ-OLi and BQ-OLi electrodes at different cycle numbers. **b** Impedance values of the NDI-OLi electrode at different cycling numbers analyzed via in situ impedance tests. **c** In situ impedance of NDI-OLi electrode at different potentials. **d** Ex situ Raman, **e** in situ FTIR, and **f** ex situ O 1*s* XPS spectra of NDI-OLi during cycling

### Reaction Kinetics and Charge Storage Mechanism

To gain further insight into the electrochemical kinetics of the NDI-OLi electrode, CV tests were performed at scan rates ranging from 0.2 to 2 mV s^−1^ (Fig. S32a). The *b* values for all redox peaks of NDI‑OLi range from 0.75 to 0.85 (Fig. S32b), suggesting that the electrochemical process involves both capacitive and diffusion‑controlled behaviors. The capacitive contribution was further quantified using the equation *i* = *k*_1_*ν* + *k*_2_*ν*^1/2^, where *k*_1_ and *k*_2 _represent the contributions from surface capacitive and diffusion‑controlled processes, respectively [51]. According to the calculation results, the contribution rate of capacitive control capacity (red region in Fig. S32c) is approximately 81% at 0.2 mV s^−1^, and further increases to 91% at 2 mV s^−1^. Such a high capacitive contribution confirms the favorable electrochemical kinetics of NDI‑OLi.

The reaction kinetic of NDI-OLi was further evaluated via electrochemical impedance spectroscopy (EIS). As shown in Figs. 3a and S33, the charge transfer resistance (*R*_ct_) of NDI-OLi stabilizes rapidly after 10 cycles, increasing only slightly from 73.0 Ω (5th cycle) to 79.9 Ω (10th cycle). In sharp contrast, after 10 cycles, the charge transfer impedances of AQ-OLi and BQ-OLi electrodes reached 268.5 and 109.9 Ω, respectively, far exceeding that of NDI-OLi. This comparison highlights the superior charge transfer kinetics of the NDI-OLi cathode. In addition, the kinetic behaviors of the three materials were compared using EIS. From the linear region at low frequencies, the Warburg impedance coefficient (*σ*_*w*_) can be obtained by fitting the relationship between Z’ and *ω*^*−*1/2^, which is correlated with lithium-ion diffusion characteristics. The inset in Fig. S34 clearly shows that the slope of the curve for NDI-OLi is smaller than those for AQ-OLi and BQ-OLi, suggesting faster lithium-ion diffusion in NDI-OLi, thereby contributing to its outstanding rate performance. The lithium-ion diffusion coefficient (*D*_Li⁺_) was calculated using the following equation: *D*_Li⁺_ = *R*^2^*T*^2^/(2*A*^2^*n*^4^*F*^4^*C*^2^*σ*_*w*_^2^), where *R*, *T*, *A*, *n*, *F*, and *C* represent the gas constant, absolute temperature, electrode surface area, number of electrons transferred per repeat unit, Faraday constant, and lithium-ion concentration, respectively [52]. The calculated diffusion coefficient for NDI-OLi is 1.7 × 10^−14^ cm^2^ s^−1^, which is significantly higher than those of the reference materials AQ-OLi (8.96 × 10^−16^ cm^2^ s^−1^) and BQ-OLi (1.45 × 10^−15^ cm^2^ s^−1^), further confirming the superior ion transport kinetics of NDI-OLi.

To further track the kinetic evolution, we conducted in situ impedance tests at different cycling numbers (at 1 A g^−1^). As shown in Figs. 3b and S35, the NDI-OLi electrode exhibits a decreasing trend in resistance within the first 200 cycles, which may be attributed to the nanoscale morphological evolution. As shown in Fig. S36, compared with the pristine electrode, NDI‑OLi exhibits distinct nanosizing behavior after 200 cycles. In the subsequent cycles (500 cycles), the particle morphology remains stable without significant changes. Notably, even after extended cycling for 1000, and up to 5000 cycles, NDI‑OLi is able to retain the previously formed nanoparticle morphology, indicating excellent structural flexibility and stability. This structural flexibility also further ensures the mechanical integrity of the electrode during long-term cycling. In addition, this granular arrangement enhances interfacial contact and improves electron transport efficiency. Importantly, this morphological transition does not compromise redox activity, as the charge–discharge profiles maintain stable voltage plateaus and excellent capacity retention (Fig. S23). After 200 cycles, the resistance stabilizes at a low level, fully demonstrating that the NDI-OLi electrode possesses rapid kinetic characteristics and excellent cycling stability. Furthermore, the impedance evolution of NDI-OLi under different redox states was also investigated, with results presented in Fig. 3c. During the discharge phase, the electrode impedance experiences a slight increase, which subsequently decreases during charging. Overall, the resistance fluctuations remain minimal, indicating excellent structural stability of the NDI-OLi electrode throughout charge–discharge cycles.

The lithium-ion diffusion coefficient was further calculated by the galvanostatic intermittent titration technique (GITT). As shown in Fig. S37, the IR voltage drop of the NDI-OLi electrode is significantly lower than that of AQ-OLi and BQ-OLi, suggesting reduced polarization. Concurrently, the average Li^+^ diffusion coefficient of NDI-OLi during discharge (10^−9.5^ cm^2^ s^−1^) exceeded those of BQ-OLi (10^−9.9^ cm^2^ s^−1^) and AQ-OLi (10^−10.3^ cm^2^ s^−1^), demonstrating more efficient kinetic processes (Fig. S38). This result confirms that the NDI-OLi electrode exhibits rapid energy storage kinetics, primarily attributed to its stable molecular structure, relatively high electronic conductivity, and optimized electronic structure/energy levels, which render the lithium-preferred redox-active sites highly accessible and enable efficient ion diffusion.

In order to investigate the storage mechanism of the NDI-OLi electrode, the redox processes were detected by ex situ Raman, in situ FTIR and ex situ XPS spectra. As shown in Fig. 3d, the C–O stretching band at 1083 cm^−1^ exhibits an enhancement trend during discharge, while the *ν*(Li–O, 1387 cm^−1^) and *δ*(Li–O, 951 cm^−1^) vibration also demonstrate increased intensity [53]. Following recharge, these peaks gradually diminish, indicating that the carbonyl group facilitates the reversible insertion of lithium ions. Concurrently, the *π*-conjugated benzene ring vibrations show analogous modulation patterns, confirming the coupled electronic and structural changes during redox reactions. The in situ FTIR spectroscopy analysis (Fig. 3e) confirmed the redox mechanism of NDI-OLi through the systematic evolution of characteristic vibrational modes. With the background of the open-circuit spectrum, the spectra of the NDI-OLi electrode were collected at various charge–discharge states. During discharge, as electrons are gained, the carbonyl group combines with lithium ions to form C–O–Li bonds to balance the charge. Consequently, the absorption peak at C=O (1660 cm^−1^) becomes weaker, while the absorption peak at C–O (1190 cm^−1^) becomes stronger. Upon recharging, these spectral features undergo a complete reversal, confirming the high reversibility of the redox chemistry [54, 55]. Reversible alterations in the C=C mode near 1580 cm^−1^ were also observed, arising from electron redistribution in the NDI-OLi molecular structure during discharge, which enhances the aromaticity of naphthalene and imine rings [47]. These results are consistent with prior theoretical calculations (Fig. S9), indicating that NDI-OLi exhibits increased aromaticity upon electron acquisition. In addition, the evolutionary behavior of C=O in NDI-OLi was further demonstrated by in ex- situ O 1*s* XPS spectra. As shown in Fig. 3f, the peak assigned to the C=O group decreases after discharged to 1.5 V, along with an enhancement of the C–O group. Upon recharging, the intensities of the C=O and C–O bonds have a contrary tendency, further implying highly reversible redox behavior of C = O/C–O[19]. To avoid interference from the surface SEI layer covering the bulk C=O signals, depth-profiling XPS with argon-ion sputtering was performed on electrodes after 5 cycles (sputtering time: 5 and 10 min). As shown in Fig. S39, the intensity and ratio of the C=O/C–O peaks show no significant variation with sputtering depth, either in the fully charged or fully discharged states. This indicates that the surface SEI layer does not obscure the bulk C=O signals. These results indicate that the C=O group in NDI-OLi serves as the primary active site for Li⁺ storage. The synergistic electron redistribution throughout the *π*-conjugated system during the redox process ensures the structural robustness of NDI-OLi, thereby underpinning its superior electrochemical stability and high-rate performance.

### Theoretical Calculation

The experimental discharge curve of NDI-OLi exhibits two distinct voltage plateaus, each corresponding to an approximately single‑electron transfer process, which suggests a sequential two‑step lithiation mechanism. However, four pairs of redox peaks (instead of two) are observed in the cyclic voltammetry (CV) of NDI‑OLi. To explain this discrepancy, first‑principles density functional theory (DFT) calculations were performed. Owing to the positional effect of the -OLi substituent, the carbonyl groups in the molecule reside in different chemical environments. This leads to two possible pathways for accepting the first electron during reduction (Fig. S40). While the first pathway is energetically more favorable (− 4.22 eV) and tends to attract more lithium ions, the second pathway remains accessible (− 4.19 eV). This accounts for the composite nature of the first cathodic reduction peak, consisting of a main peak at 2.85 V and a shoulder near 2.71 V. Similarly, the storage of the second lithium ion may also proceed via two distinct pathways, resulting in a main peak at 2.31 V and a shoulder near 2.26 V. Overall, the lithiation behavior of NDI‑OLi can be effectively described by a simplified two‑step mechanism.

**Fig. 4:**
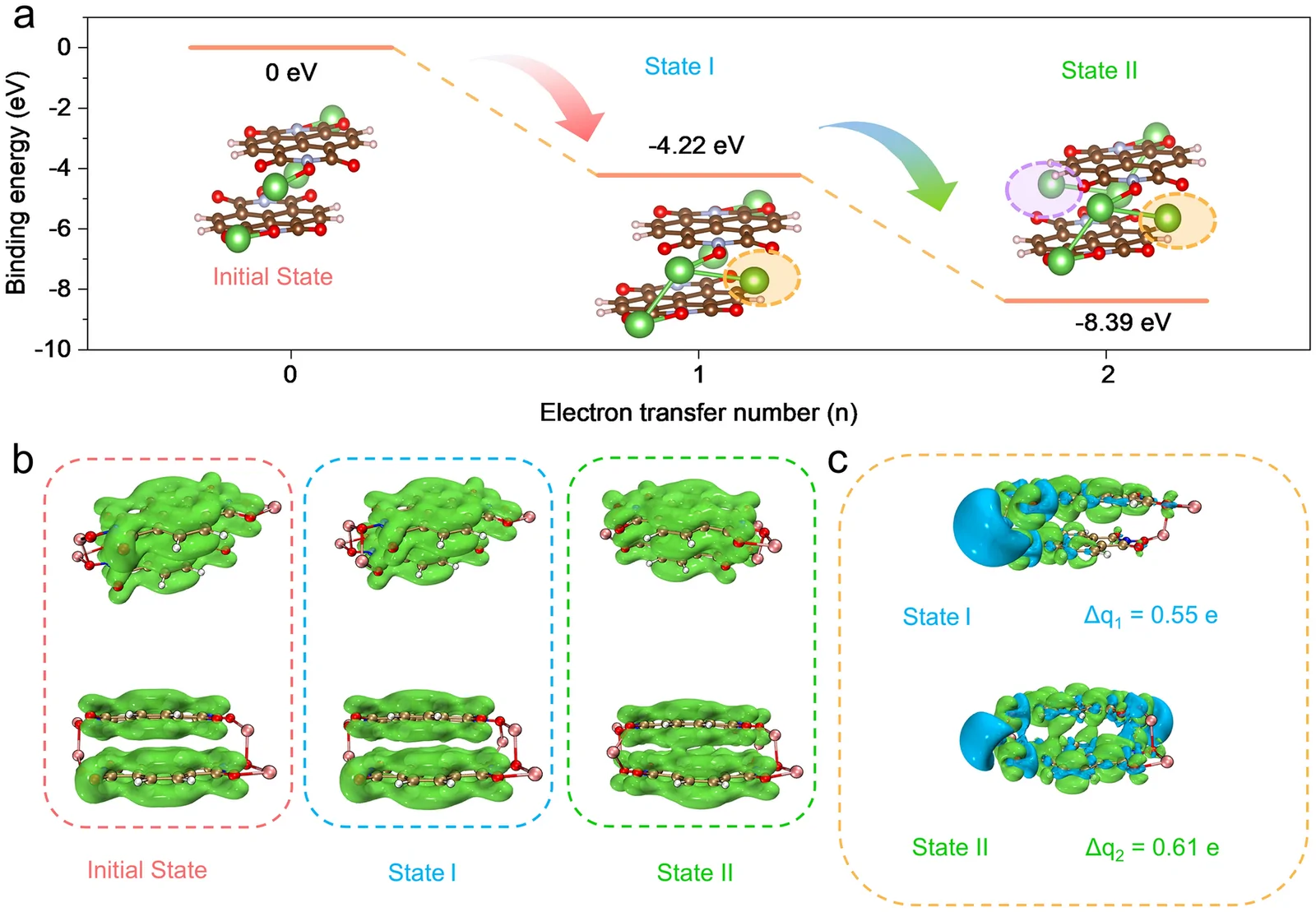
Theoretical simulation of redox behaviors of NDI-OLi under various electrochemical states. **a** Calculated binding energies of NDI-OLi with optimized geometries. **b** ELF-π isosurfaces. **c** Charge density difference isosurfaces

To better elucidate this mechanism and based on the principle of energy minimization, we propose a two-step lithium storage mechanism for NDI-OLi (Fig. 4a). In the first step, a Li⁺ is stored in the interlayer of the NDI-OLi molecule and binds to the C=O groups in both NDI-OLi molecules, requiring a binding energy (*E*_*b*_) of − 4.22 eV. During the subsequent step, a second Li⁺ associates with the carbonyl sites located diagonally to the first coordination center, with the required binding energy *E*_*b*_ decreasing to − 8.39 eV. These increasingly negative *E*_*b*_ values indicate enhanced thermodynamic favorability for Li-ion association and structural stabilization upon lithiation. This strong binding affinity, combined with the structural integrity of the coordination environment, underpins the robust and reversible charge storage capability of the NDI-OLi cathode.

The *π*-electron structure of NDI-OLi under different electrochemical states was analyzed using the *π*-electron localization function (ELF-*π*) method (Fig. 4b). The results reveal that continuous ELF-*π* isosurfaces through the entire NDI-OLi molecule reflect its high conjugation, which facilitates electron delocalization. Notably, upon binding one (State I) and two (State II) lithium ions, the discharged structures of NDI-OLi exhibit significantly intensified *π*-electron isosurface distributions, indicating strong aromaticity and thermodynamic stability in their reduced states. Furthermore, the bonding property of lithium ions combined with the NDI-OLi structure was elucidated through charge density differential isosurfaces. As shown in Fig. 4c, the charge depletion of Li⁺ and charge accumulation in the C=O region indicate that stable configurations form via strong chemical interactions. Bader charge analysis reveals significant charge transfer from Li⁺ to the C=O groups (State I: 0.55 e; State II: 0.61 e), suggesting the chemical interaction between Li⁺ and C=O. Overall, NDI-OLi achieves complete accessibility of the redox-active carbonyl group through its rapid reaction kinetics and stable lithium-ion coupled redox reaction, thereby ensuring stable electrochemical storage.

### Full Cells

**Fig. 5:**
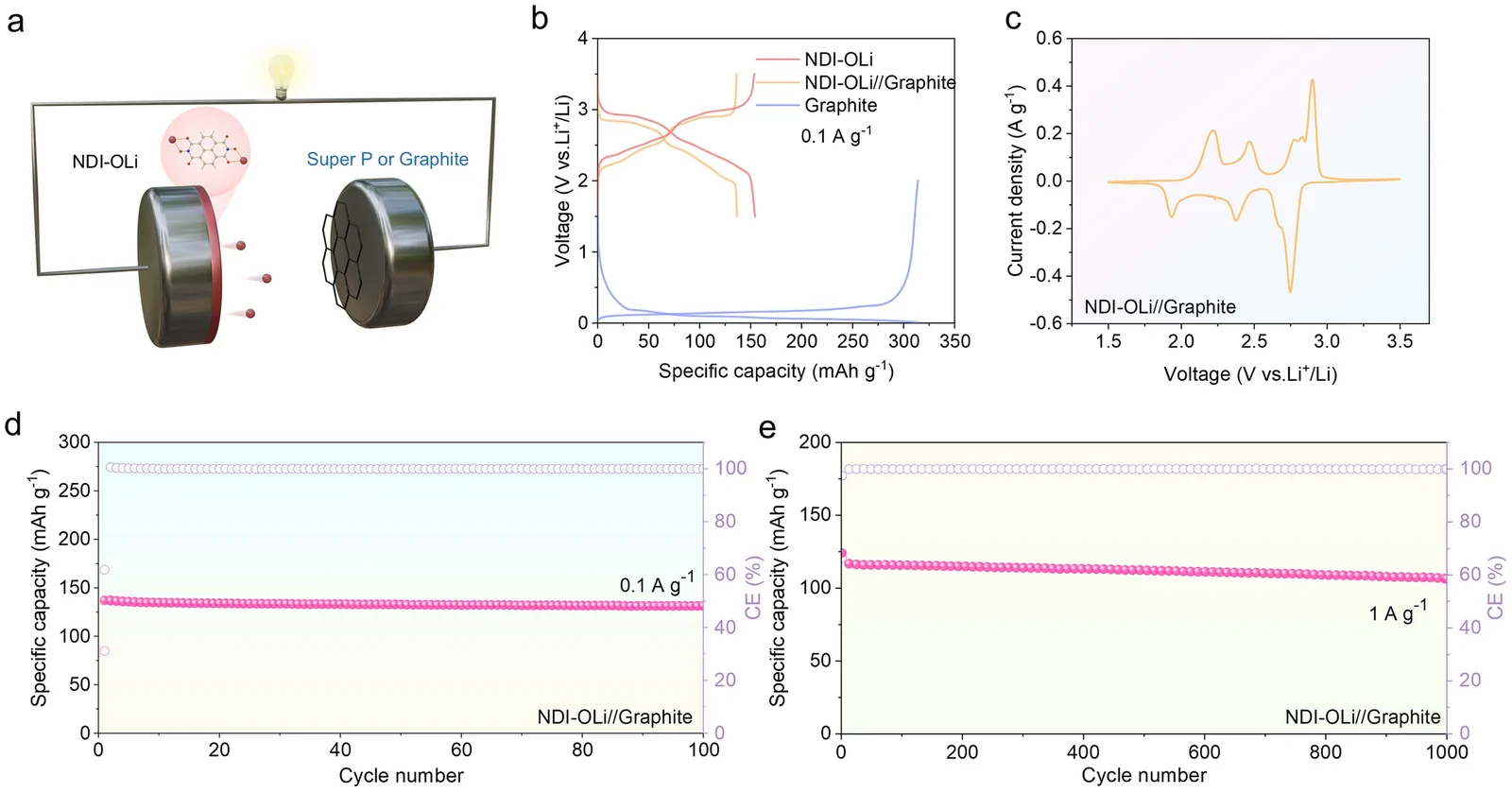
**a** Schematic diagram of the NDI-OLi//Graphite battery. **b** Charge–discharge curves of NDI-OLi//Graphite full cell, NDI-OLi//Li half-cell and Graphite//Li half-cell. **c** CV curve of NDI-OLi//Graphite full cell at 0.2 mV s^−1^. Cycling performance of NDI-OLi//Graphite full cell at **d** 0.1 A g^−1^ (the mass of the active material is 1.1 mg cm^−2^) and **e** 1 A g^−1^ (the mass of the active material is 1.5 mg cm^−2^)

To evaluate the practical viability of the NDI-OLi cathode, full cells were configured using either Super P (SP) or graphite anodes (Fig. 5a). Given the well-known incompatibility between traditional ether-based electrolytes and graphite (Fig. S41) [56], the NDI-OLi//graphite cell was evaluated in an ester-based system (1 M LiPF_6_ in EC/DEC), while the NDI-OLi//SP cell utilized an ether-based electrolyte (1 M LiTFSI in DOL/DME). Notably, NDI-OLi exhibits outstanding electrochemical performance across both electrolyte systems (Fig. S42), underscoring its versatile compatibility with diverse electrolyte chemistries. Accordingly, the as-assembled NDI-OLi//SP and NDI-OLi//graphite full cells deliver high reversible capacities of 140 mAh g^−1^ (113.7 μAh cm^−2^) and 136.7 mAh g^−1^ (97.7 μAh cm^−2^) at 0.1 A g^−1^, respectively (Figs. 5b and S43). Owing to the low redox potential of the graphite anode, the NDI-OLi//graphite cell yields a higher average discharge voltage of ~ 2.5 V (vs. ~ 2.1 V for NDI-OLi//SP, Figs. 5c and S43). The energy density of NDI-OLi//graphite cell calculated based on the cathode active mass reaches 342 Wh kg^−1^, when calculated based on the total active mass of both electrodes, it is 190 Wh kg^−1^, demonstrating excellent energy output characteristics. In terms of cycling stability, the NDI-OLi//graphite full cell demonstrates exceptional performance. It exhibits negligible capacity decay after 100 cycles at 0.1 A g^−1^ (Fig. 5d). Remarkably, even after 1000 cycles at a high current density of 1 A g^−1^, the capacity retention remains above 95% (Fig. 5e). Moreover, the full cell also exhibits good rate capability, delivering reversible capacities of 133, 126.7, 121.5, 112.5, and 105.2 mAh g^−1^ at current densities of 0.1, 0.5, 1, 2, and 3 A g^−1^, respectively (Fig. S44). These findings not only confirm the adaptability of NDI-OLi to ester-based electrolyte but also establish a solid experimental foundation for the development of high-performance organic full cells.

## Conclusion

In summary, a naphthalenediimide-based lithium salt (NDI-OLi) has been successfully developed, integrating a robust *π*-conjugated framework, high aromaticity, and relatively high electronic conductivity. By virtue of its stable *π*-conjugated structure and strong intermolecular interactions, NDI-OLi achieves accelerated electron transfer and structural integrity during cycling. The synergy between relatively high electronic conductivity and stable delocalized electronic geometry enables NDI-OLi to exhibit an outstanding reversible capacity (160 mAh g^−1^ at 0.1 A g^−1^) and superior rate capability (99.9 mAh g^−1^ at 8 A g^−1^). Notably, the electrode achieves a remarkable cycling stability of 5000 cycles with 85% retention, significantly surpassing most previously reported organic lithium salt counterparts. Furthermore, the assembled full battery also exhibits favorable electrochemical performance. We believe these findings will facilitate the exploration of organic materials with stable structures and high electronic conductivity as high-performance electrodes, advancing the development of organic energy storage batteries.

## Supplementary Information

Below is the link to the electronic supplementary material.

Supplementary file1 (DOCX 37314 KB)
