# Supplementary material for: Highly Conductive and Stable Naphthalenediimide-Based Organic Salt Cathode for Robust Lithium-Ion Batteries
Source: Nanomicro Lett. 2026 Apr 8;18:322. doi: 10.1007/s40820-026-02176-x (PMC13062141; doi:10.1007/s40820-026-02176-x)
Supplement: Supplementary file 1 — Supplementary file1 (DOCX 37314 KB) [file 40820_2026_2176_MOESM1_ESM.docx]

Supporting Information for

**Highly Conductive and Stable Naphthalenediimide-Based Organic Salt Cathode for Robust Lithium-Ion Batteries**

Xiangyu Su^1,#^, Zixuan Shan^1,#^, Xuan Peng^1^, Jianyi Chu^1^, Zhihao Jia^1^, Yanan Kou^1^, Min Jiang^2,3,^*, and Yuan Chen^1,^*

^1^ College of Energy Materials and Chemistry, State Key Laboratory of New Textile Materials and Advanced Processing, Inner Mongolia University, Hohhot 010070, P. R. China

^2^ Department of Physics, City University of Hong Kong, Hong Kong 999077, P. R. China

^3^ School of Materials Science and Engineering, Shanghai Jiao Tong University, Shanghai 200240, P. R. China

^#^ Xiangyu Su and Zixuan Shan contributed equally to this work.

*Corresponding authors. E-mail: [yuanchen@imu.edu.cn](mailto:yuanchen@imu.edu.cn) (Yuan Chen); [Min.Jianghh@cityu.edu.hk](mailto:Min.Jianghh@cityu.edu.hk) (Min Jiang)

**S1** **Experimental Section**

**S1.1 Material Characterization**

Thermogravimetric analysis (TGA) was conducted on a TG209 F1 under nitrogen flow (20 mL min^−1^) with a heating rate of 10 ^o^C min ^−1^ (20~800 °C). Scanning electron microscope (SEM) images were obtained on a Gemini SEM 360 at Shiyanjia Lab (https://www.Shiyanjia.com). The Fourier transform infrared (FTIR) spectra were recorded on IRAffinity-1s with samples prepared as KBr pellets. X-ray photoelectron spectra (XPS) were carried out by the ESCALab 250Xi using 200 W monochromated Al Kɑ radiation. All spectra were charge-corrected relative to the C 1s component at 284.8 eV binding energy and analyzed using Thermo Avantage software. The authors would like to thank Xiao Ming Li from SCI-GO (www.sci-go.com) for the XPS analysis. UV-Vis-NIR absorption spectrum was recorded on cary5000. Gas chromatography-mass spectrometry (GC-MS) was performed using an Agilent 8890-5977C system. In situ attenuated total reflection-Fourier transform infrared spectrometer (ATR-FTIR, Nicolet IS50) was performed to analyze the electrochemical energy storage mechanism. In situ ATR-FTIR cell was purchased from the Beijing Scistar Technology Co. Ltd. For ex situ XPS characterization, the cells were cycled to a certain state of charge at a current of 0.1 A g^-1^. The conductivity of the materials was measured using a four‑probe method. All measurements were performed on a CTA-3 electrical property measurement system at room temperature. The sample was pressed into a pellet and securely fixed onto the testing electrode. The system was purged with high-purity helium 2-3 times and maintained at a suitable negative pressure for safety. The electronic conductivity reported in this study was measured using pure NDI-OLi powder without the addition of any conductive additives.

**S1.2 Electrochemical Measurement**

The electrodes (NDI-OLi, AQ-OLi and BQ-OLi) were prepared by mixing the active material, Super P, and sodium carboxymethylcellulose (CMC) binder in the ratio of 7:2:1 by weight using deionized water as the solvent. For the lithium iron phosphate (LFP) control electrode, LFP powder, Super P conductive carbon, and polyvinylidene fluoride (PVDF) binder were thoroughly mixed in a 7:2:1 mass ratio. Finally, all mixed slurries are evenly coated onto aluminum foil, then dried overnight under vacuum at 80 ^o^C. The mass loading of the active materials was about 1~13 mg cm^-2^. The assembly of CR2032 coin cell was carried out in an Ar-filled glovebox. Lithium metal used as the counter electrode, glass fibers or polypropylene film used as the separator, and 1 M LiTFSI in DME/DOL (1:1, v:v) used as the electrolyte. Cyclic voltammetry (CV) and electrochemical impedance spectroscopy (EIS) were performed on an electrochemical workstation (CHI 760E). EIS tests were conducted using CR2032-type coin cells. For the ex situ EIS measurement, the cell was first discharged to 1.5 V at 0.1 A g^-1^, followed by a 1h rest, before the impedance measurement was performed on an electrochemical workstation. The EIS frequency range was set from 10 mHz to 1 MHz with an amplitude of 10 mV. All tests were carried out at room temperature. The electrochemical tests were performed using the Neware battery test system (MIHW-200-160CH-B, Shenzhen, China). For the galvanostatic intermittent titration technique (GITT) test, the pulse duration was 10 minutes and the rest period was 30 minutes. The specific capacities of the half-cells and full-cells were calculated based on the weight of NDI-OLi.

**NDI-OLi//Super P full cell**. Super P (SP) anodes were prepared in the same way as described above, with the difference that the weight ratio of Super P: PVDF was 9:1. The full cells were assembled in a similar procedure. Before assembling the full cell, the SP anode was pre-lithiated (electrodes discharged to 0.01 V at a current of 0.1 A g^-1^). The electrolyte is 1 M LiTFSI in DOL/DME (1:1, v/v). In the full cell, the active mass of the cathode and anode was 1.5 and 1.3 mg, respectively.

**NDI-OLi//graphite full cell**. The preparation of graphite anodes follows the same methodology as that for SP anodes. Prior to full cell assembly, the graphite anode must be discharged to 0.01V. The electrolyte is 1M LiPF_6_ in EC/DEC (1:1, v/v). The active mass ratio between cathode and anode is 1:0.8.

**S1.3 Computational Details**

The geometry optimization and frequency analysis were calculated through density functional theory (DFT) method under B3LYP/6-311G (d) basic sets by Gaussian 16 program [1]. The implicit solvent model (IEPCM) was employed to describe the influence of polarity effects on the system, and DFT-D3 dispersion correction was incorporated to characterize the dispersion of structure. The HOMO-LUMO gap was analyzed using the Multiwfn 3.8 program,[2, 3] and visualized with the Visual Molecular Dynamics (VMD) software.[4] Mulliken atomic charge calculations were performed using Gaussian combined with the Multiwfn program to describe the charge transfer process during Li⁺ insertion in NDI-OLi.

**Supplementary Figures and Tables**

**
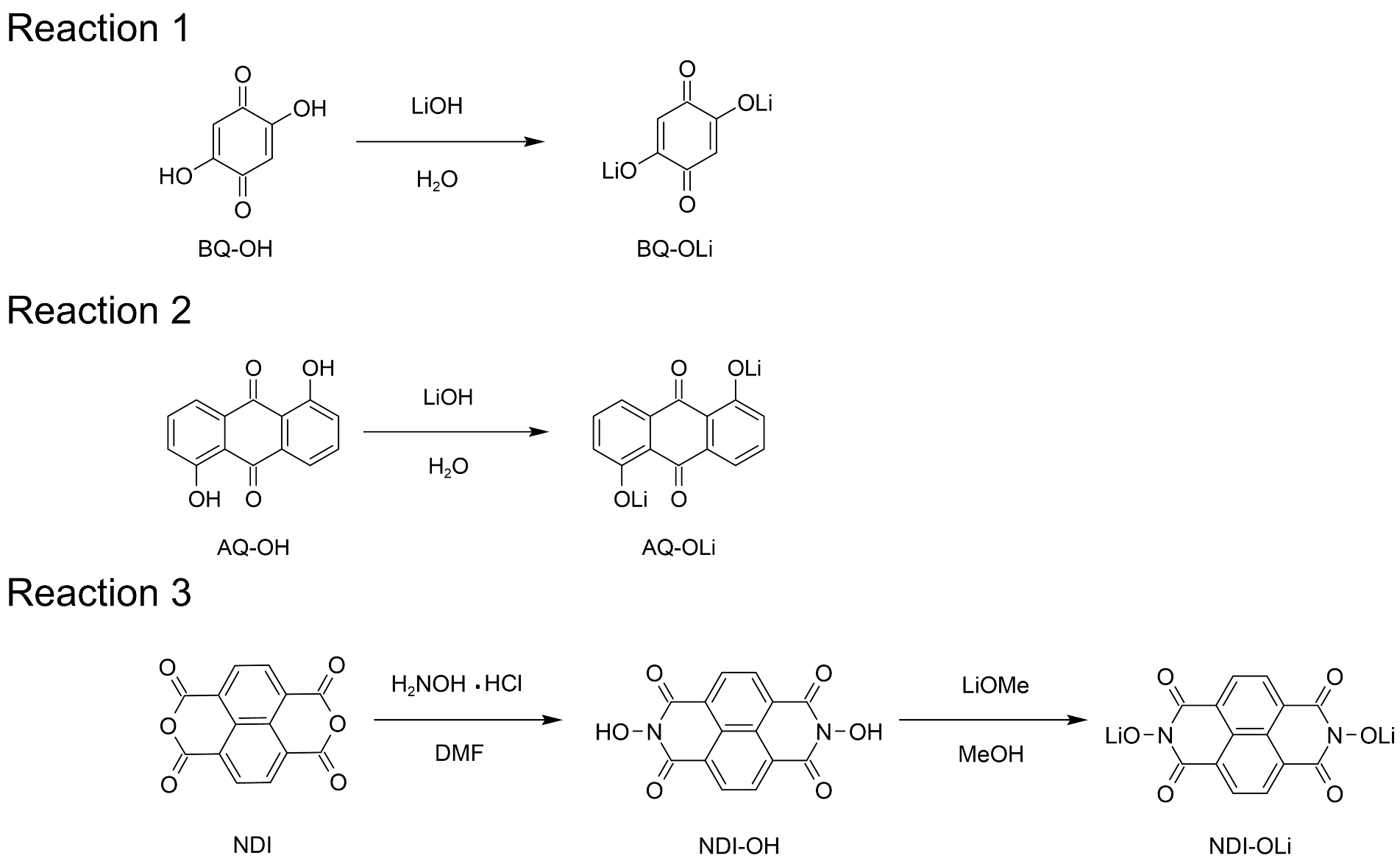
**

**Fig. S1** Schematic of synthesis of BQ-OLi, AQ-OLi and NDI-OLi

**
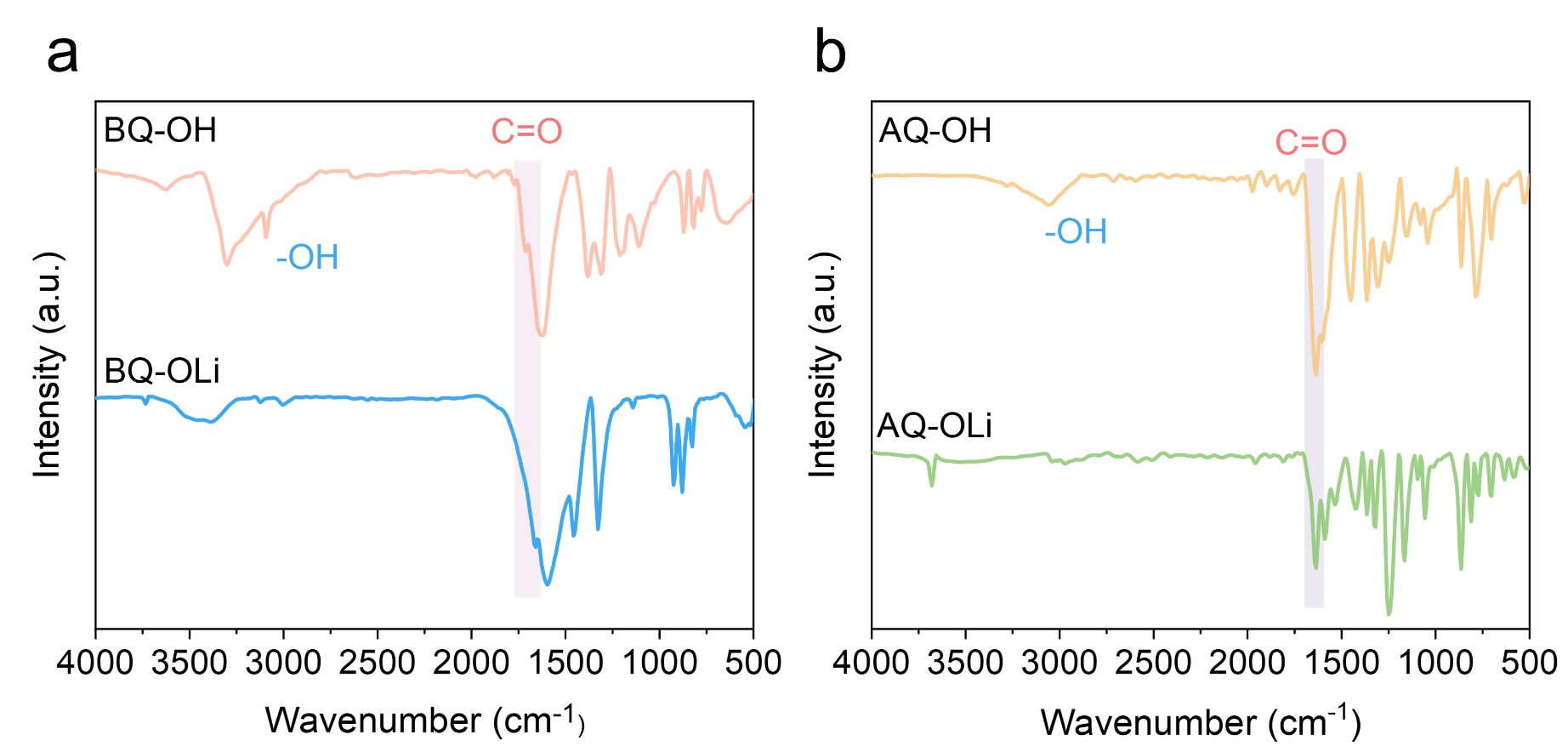
**

**Fig. S2** The FTIR spectrum of (**a**) BQ-OH, BQ-OLi, (**b**) AQ-OH and AQ-OLi, respectively

**
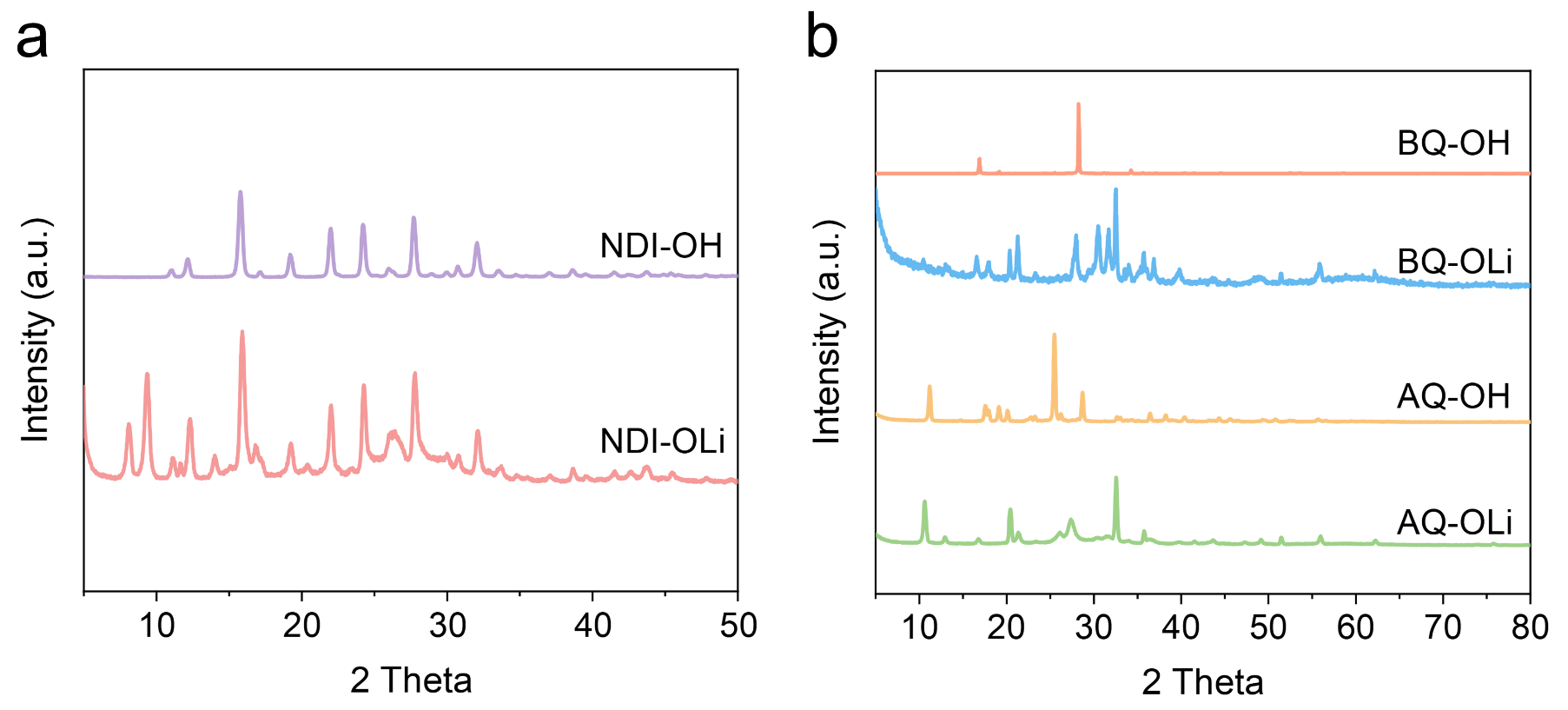
**

**Fig. S3** The PXRD patterns of (**a**) NDI-OLi, (**b**) BQ-OLi and AQ-OLi, respectively


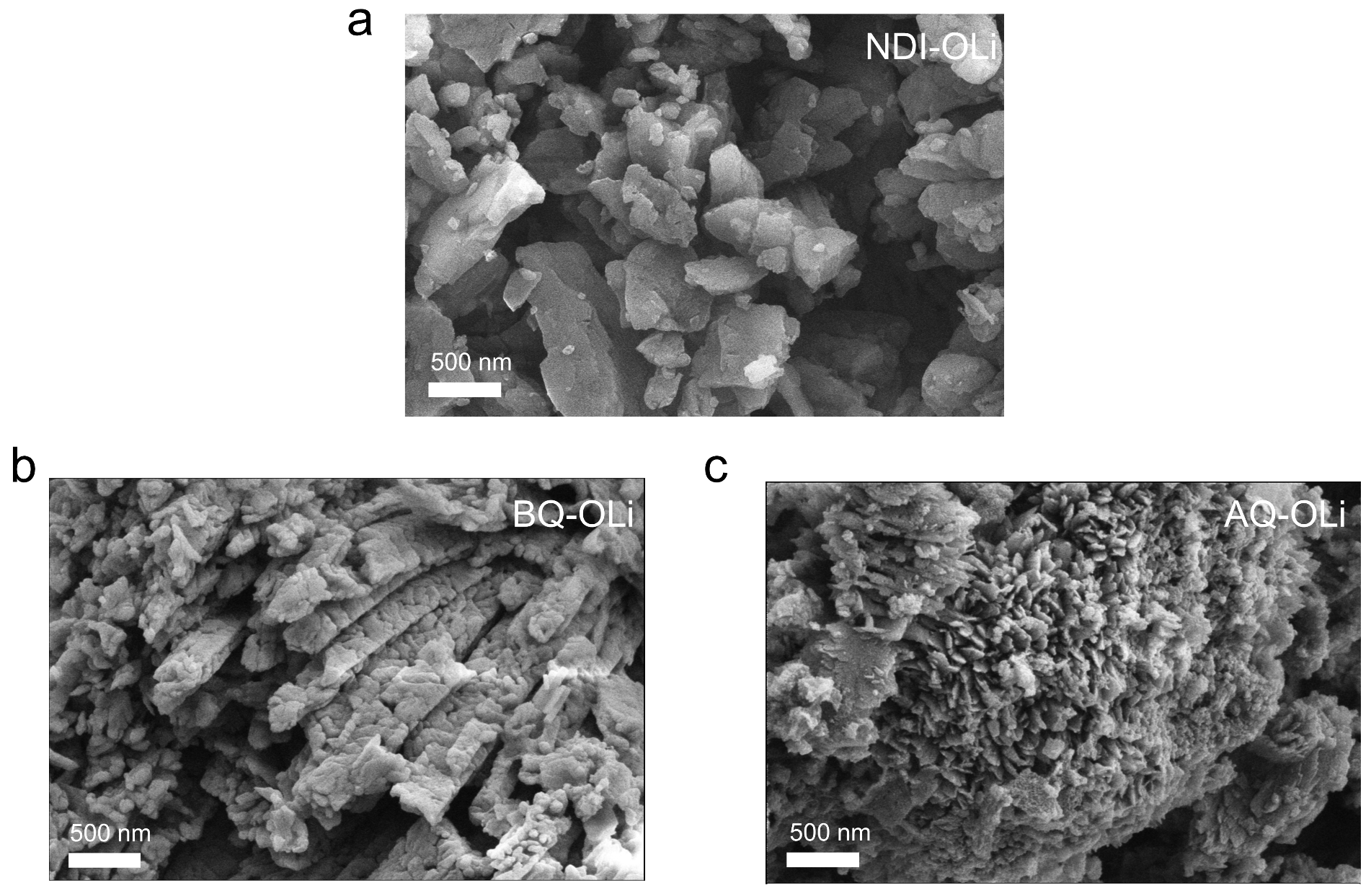


**Fig. S4** SEM images of NDI-OLi, BQ-OLi and AQ-OLi


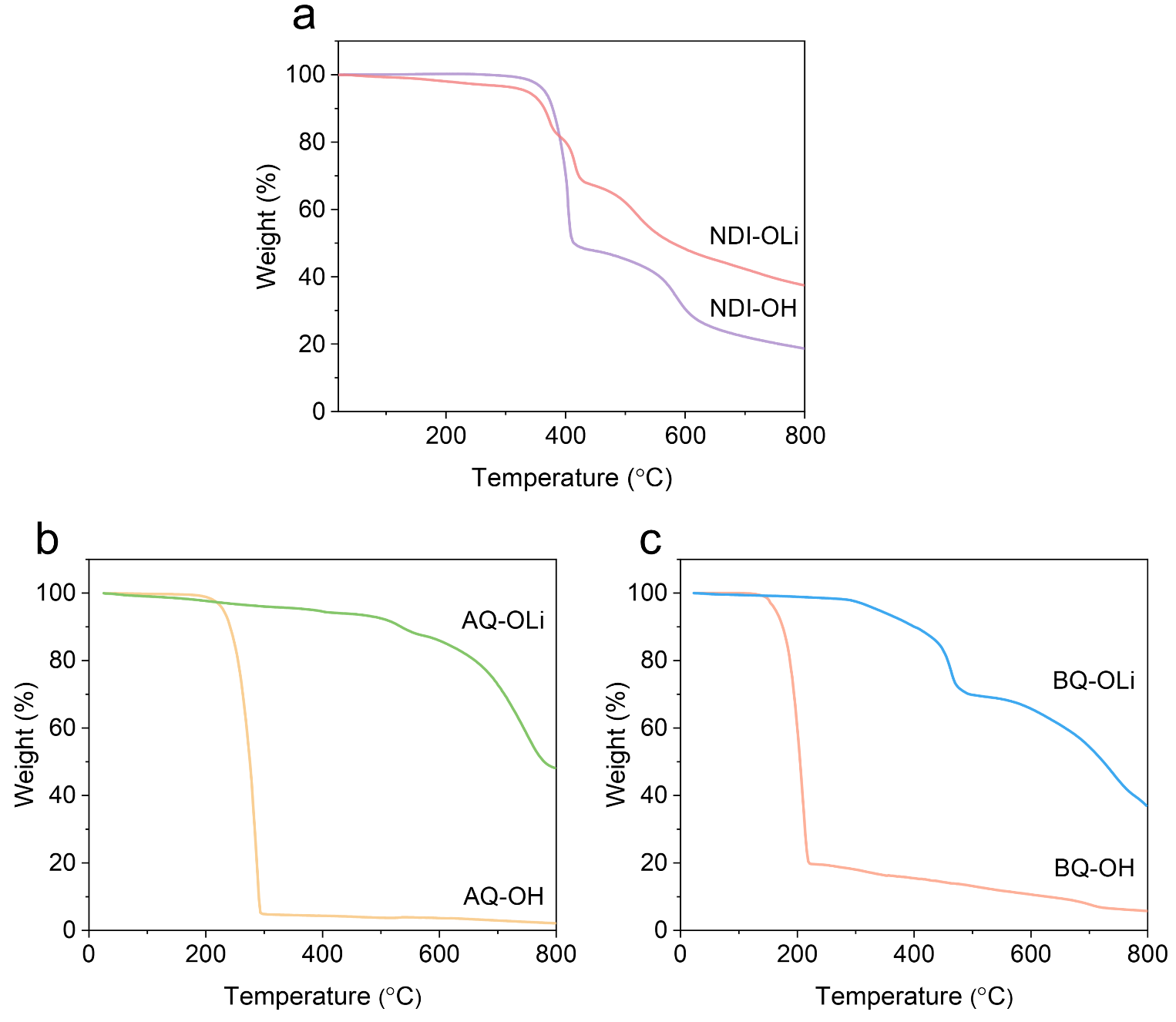


**Fig. S5** TGA curves of (**a**) NDI-OH, NDI-OLi, (**b**) AQ-OH, AQ-OLi and (**c**) BQ-OH, BQ-OLi, respectively


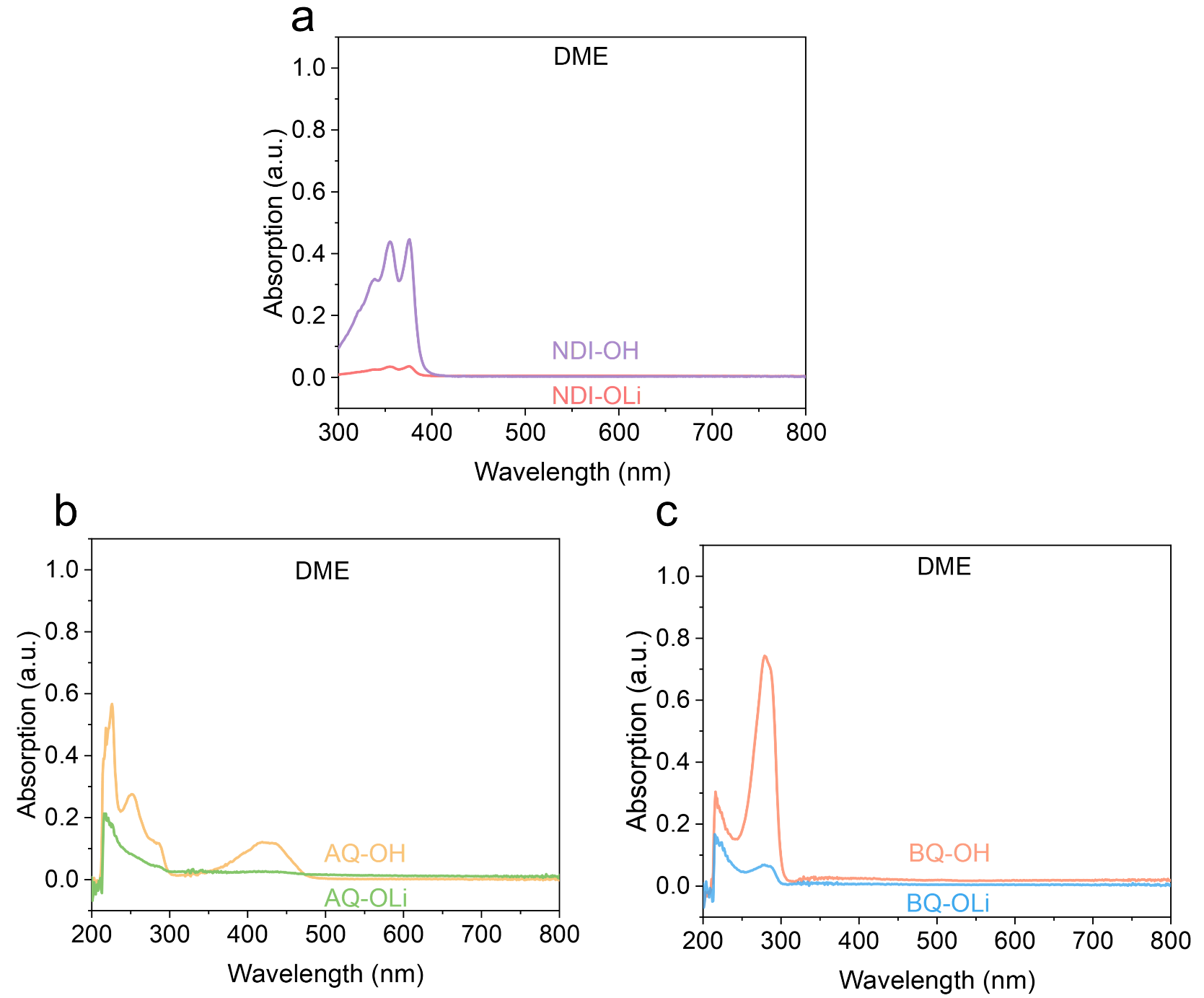


**Fig. S6** UV-Vis absorption spectra of (**a**) NDI-OH, NDI-OLi, (**b**) AQ-OH, AQ-OLi, and (**c**) BQ-OH, BQ-OLi powders in DME


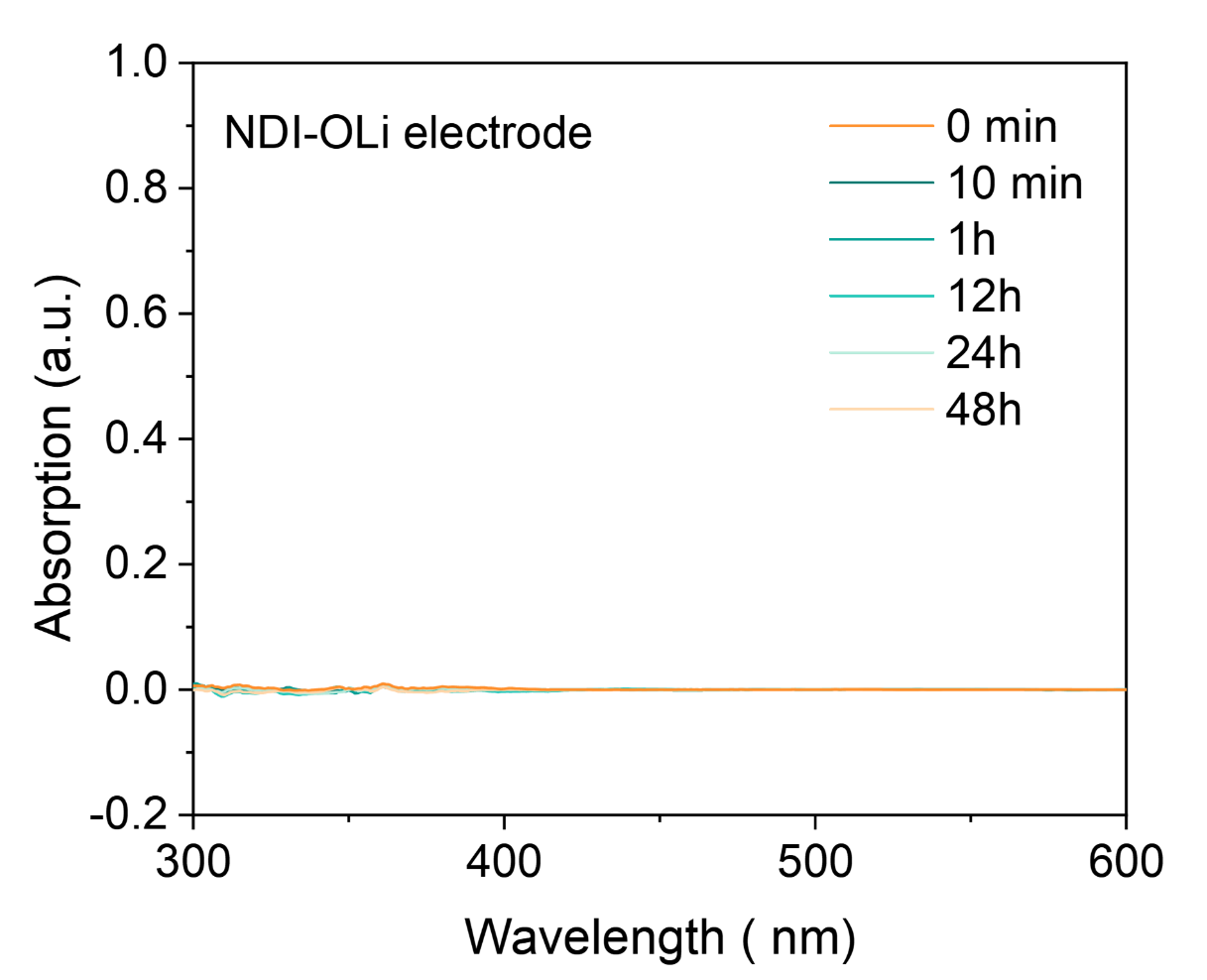


**Fig. S7** UV-Vis absorption spectra of the NDI-OLi electrode immersed in the electrolyte for different time intervals. Before each test, an equal amount of the soaked solution was taken and diluted with fresh electrolyte to the same volume


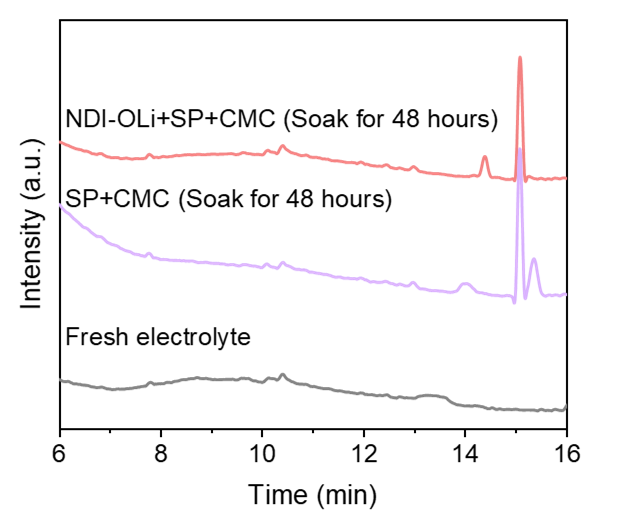


**Fig. S8** Gas chromatography of different electrodes immersed in 1M LiTFSI-DOL/DME solution

UV-Vis absorption measurement was performed on the NDI-OLi electrode (with an active material loading of 1.25 mg·cm^-2^) after different immersion periods (0 min, 10 min, 1 h, 12 h, 24 h, and 48 h). As illustrated in Fig. S7, the absence of significant characteristic absorption peaks throughout the 48 hours period confirms the negligible solubility of the NDI-OLi electrode.

As shown in Fig. S8, both the NDI-OLi electrode and the Super P/CMC-Na electrode (control sample) exhibit a set of intense peaks at 15 minutes after 48 hours of immersion. The presence of these identical peaks in the control sample indicates they do not originate from NDI-OLi dissolution, thereby ruling out the leakage of active material. This quantitative result further confirms the negligible solubility of NDI-OLi in the electrolyte.


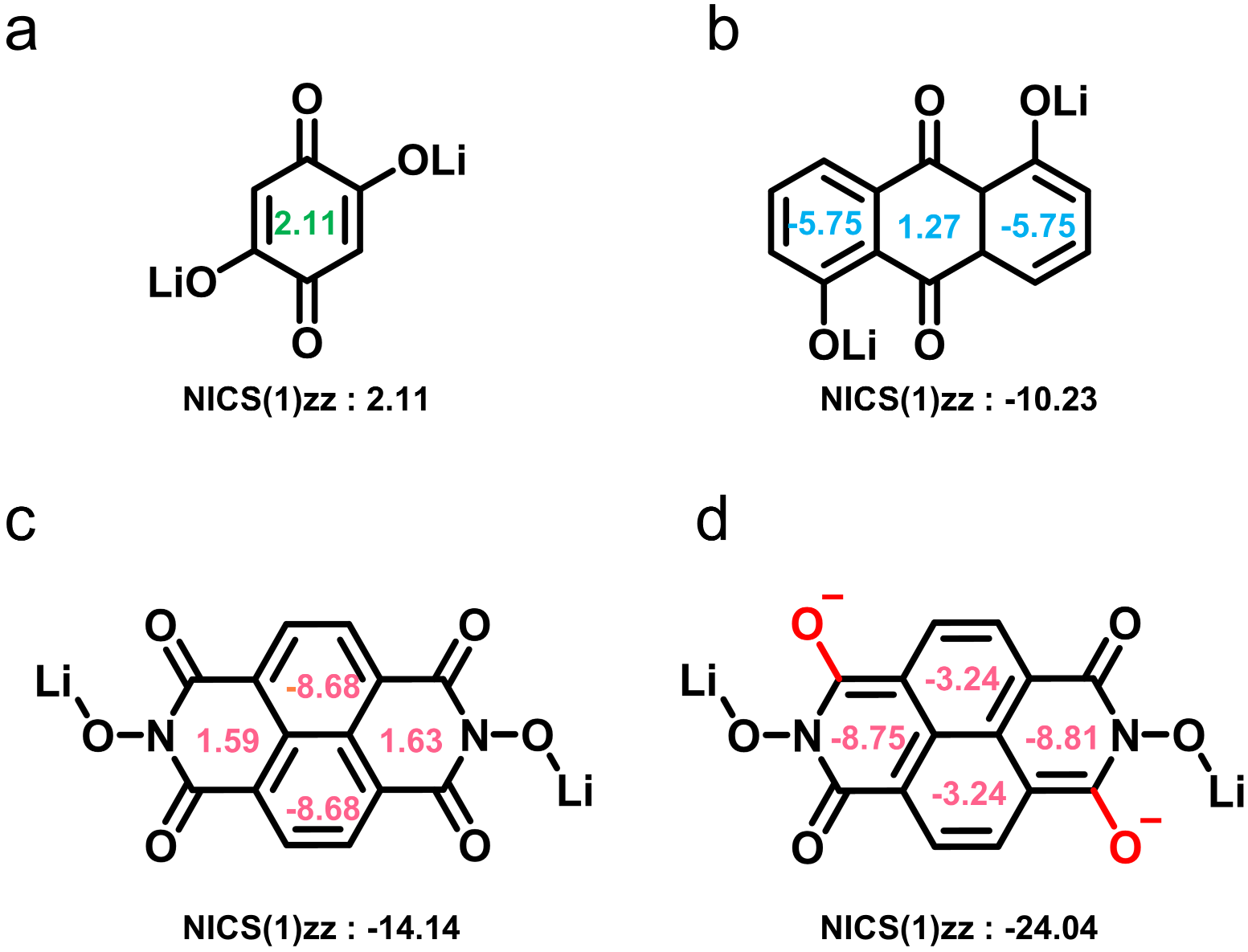


**Fig. S9** Calculated NICS(1)zz values of BQ-OLi, AQ-OLi, NDI-OLi and (NDI-OLi)^2-^

**
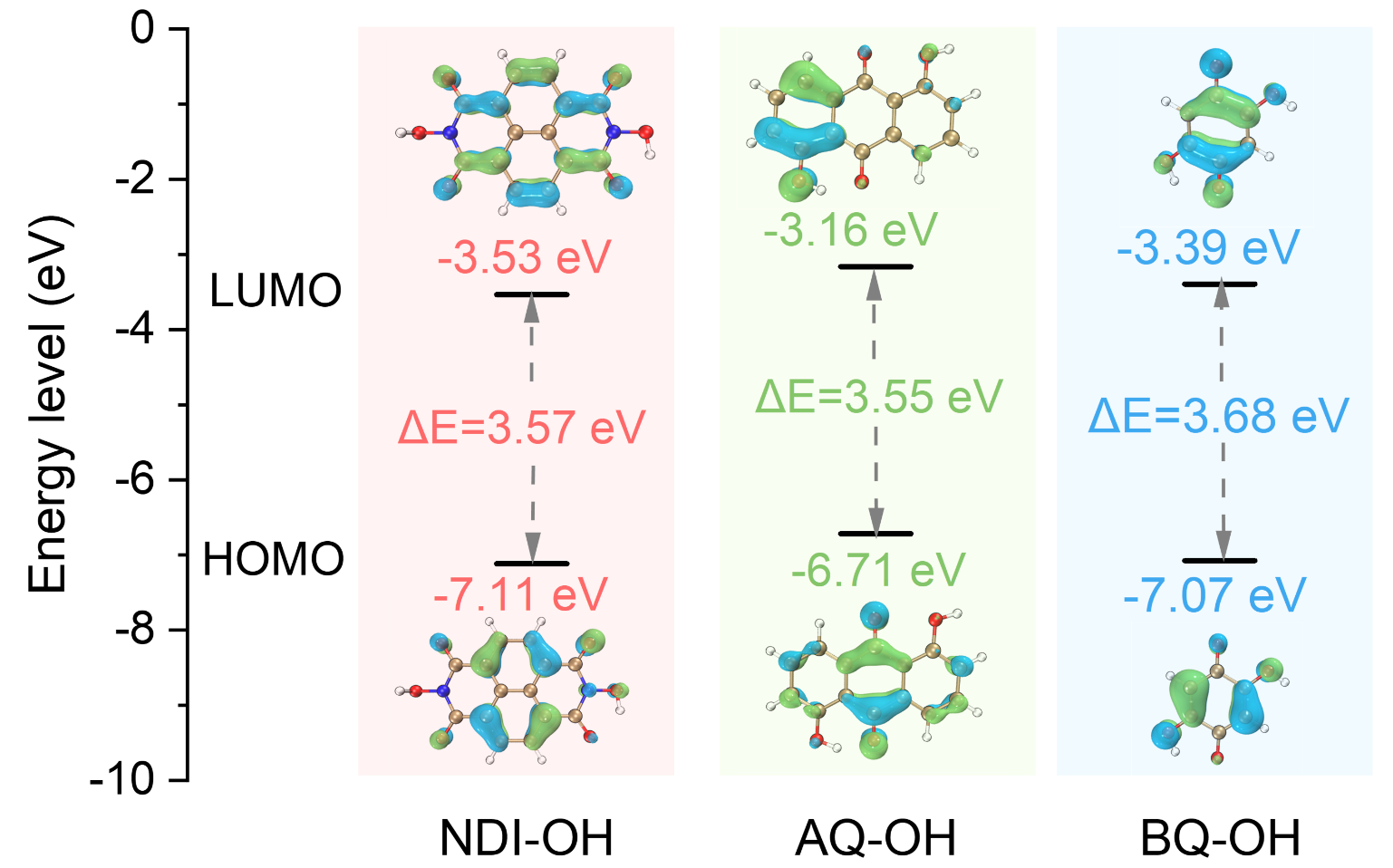
**

**Fig. S10** Calculated HOMO and LUMO energy levels of NDI-OH, AQ-OH and BQ-OH

**Fig. S11** LOL-π map of NDI-Oli


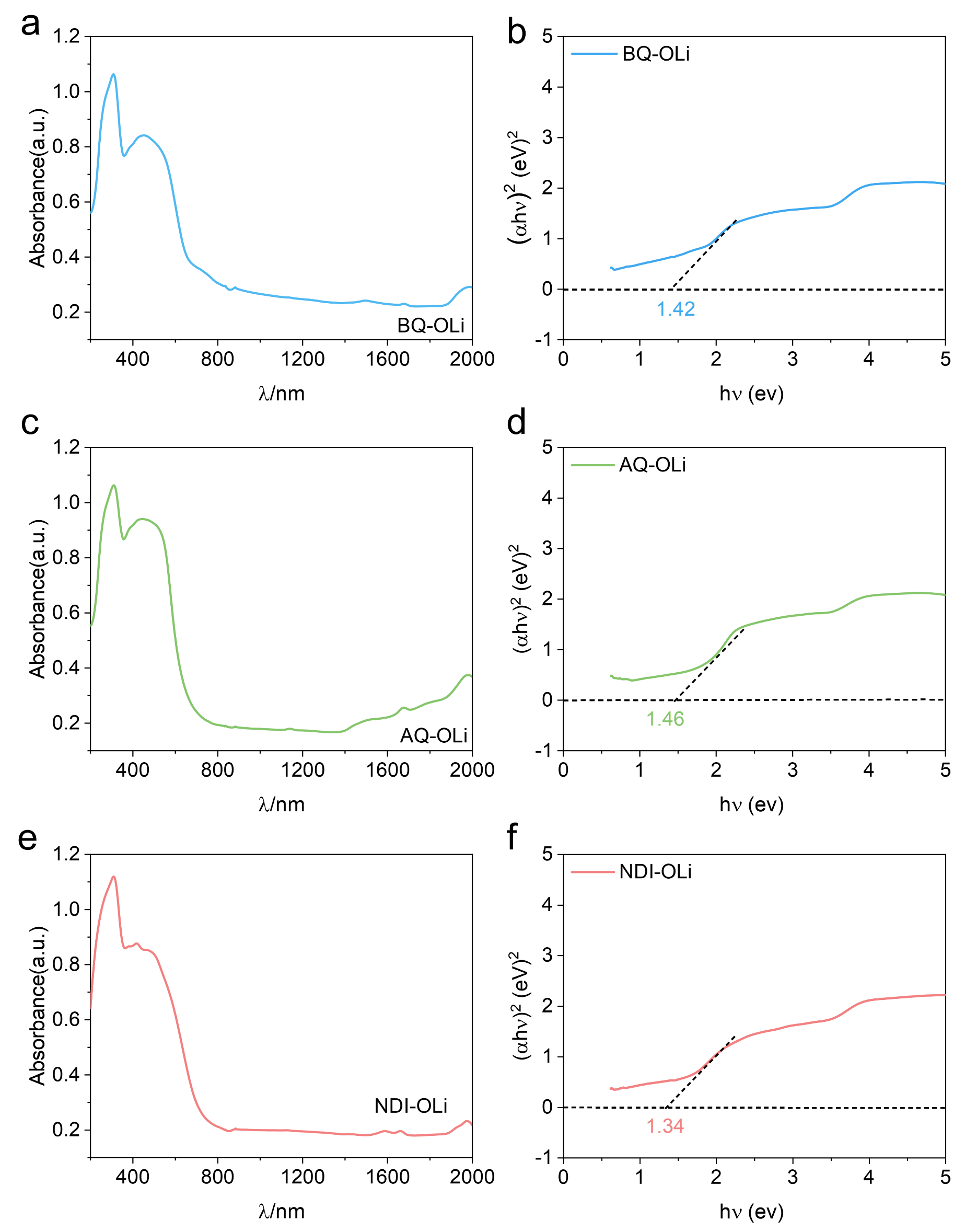


**Fig. S12** The solid-state ultraviolet testing of (**a**) BQ-OLi, (**c**) AQ-OLi and (**e**) NDI-OLi. Indirect bandgap of (**b**) BQ-OLi, (d) AQ-OLi and (f) NDI-OLi


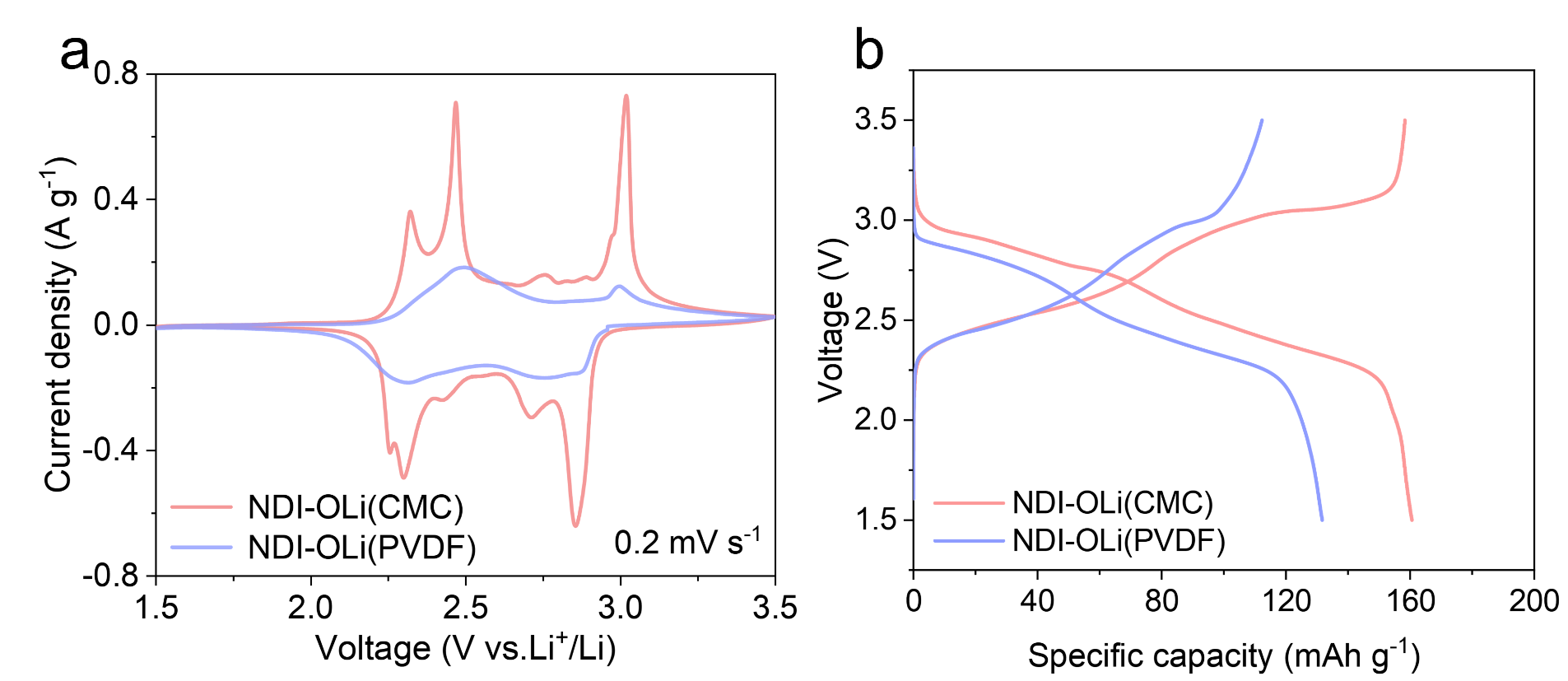


**Fig. S13** (**a**) CV curves at 0.2 mV s^-1^. (**b**) harge-discharge profile at 0.1 A g^-1^ of NDI-OLi electrodes with different binder


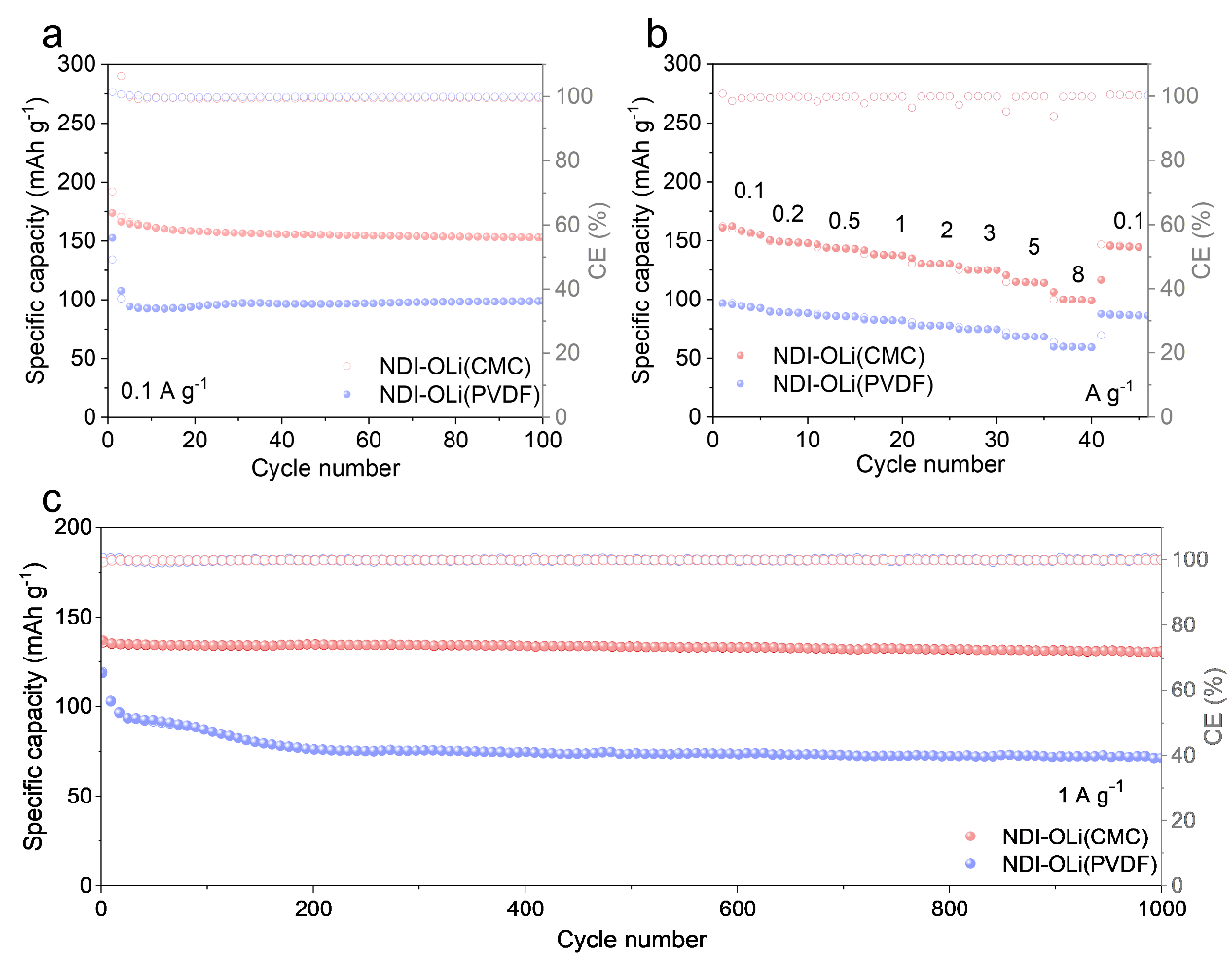


**Fig. S14 (a**) Cycling performance at 0.1 A g^-1^. (**b**) Rate performance; (**c**) Cycling performance at 1 A g^-1^ of NDI-OLi electrodes with different binders


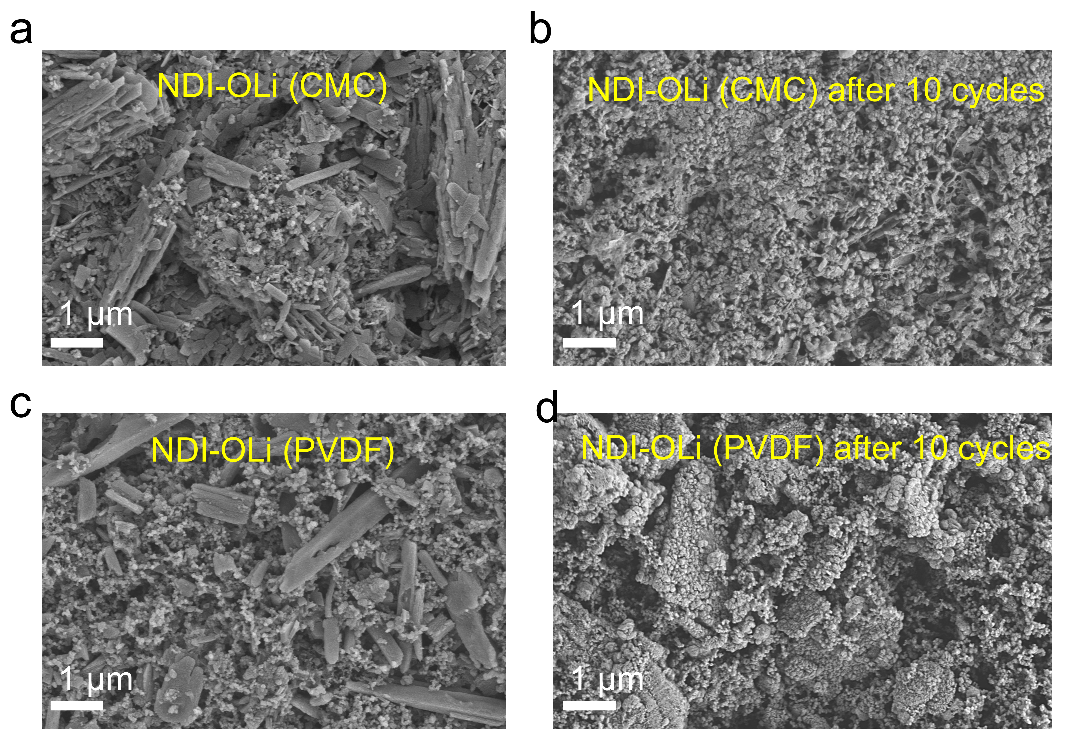


**Fig. S15** SEM images of (**a**) NDI-OLi electrode (with CMC binder), (**b**) cycled NDI-OLi electrode (with CMC binder), (**c**) NDI-OLi electrode (with PVDF binder), (**d**) cycled NDI-OLi electrode (with PVDF binder

**
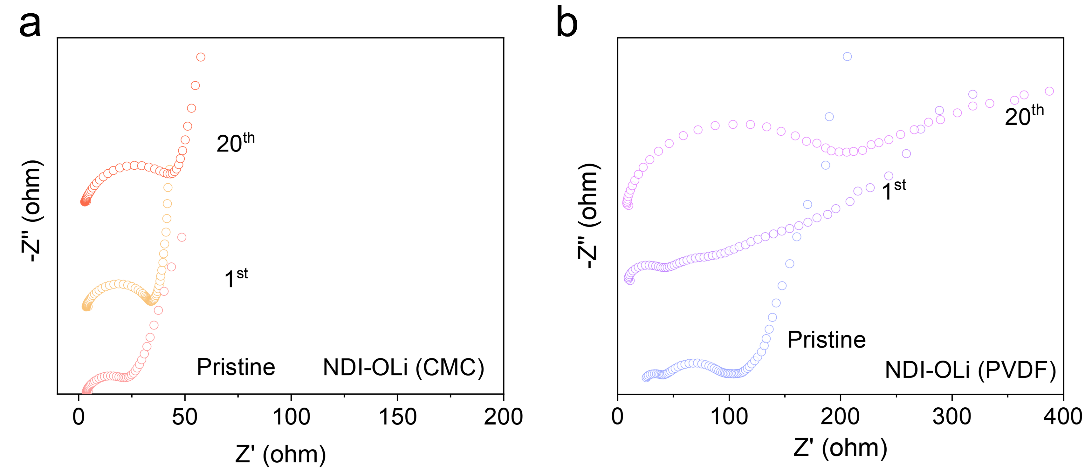
**

**Fig. S16** The Nyquist plots of (**a**) NDI-OLi cathode (with CMC binder) and (**b**) NDI-OLi cathode (with PVDF binder)


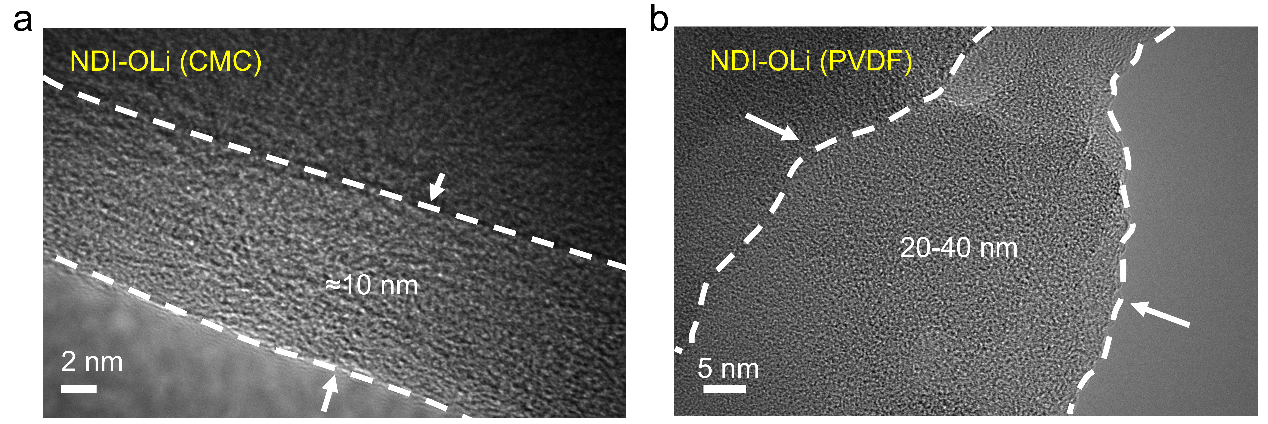


**Fig. S17** TEM of NDI-OLi electrodes using different binders


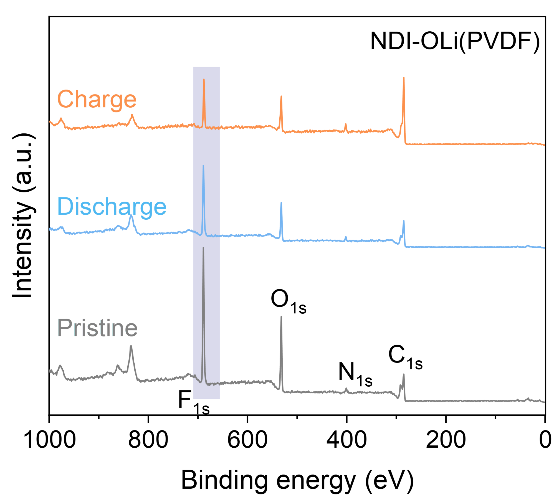


**Fig. S18** The XPS spectra of NDI-OLi using PVDF binder during cycling

XPS spectra of the NDI-OLi (PVDF) electrode revealed a significant irreversible decrease in the F signal after cycling, particularly relative to other elements such as C and N (Fig. R9). This suggests gradual loss of fluorine from the fluorinated binder during cycling. The mechanism is likely nucleophilic substitution, wherein the reduced active material acts as a nucleophile, reacting with the fluorinated binder and leading to F detachment. This defluorination weakens the binder’s adhesion, causing particle agglomeration, detachment, increased impedance, and ultimately poor electrode performance, consistent with the observed morphological changes in PVDF-based electrodes after cycling.


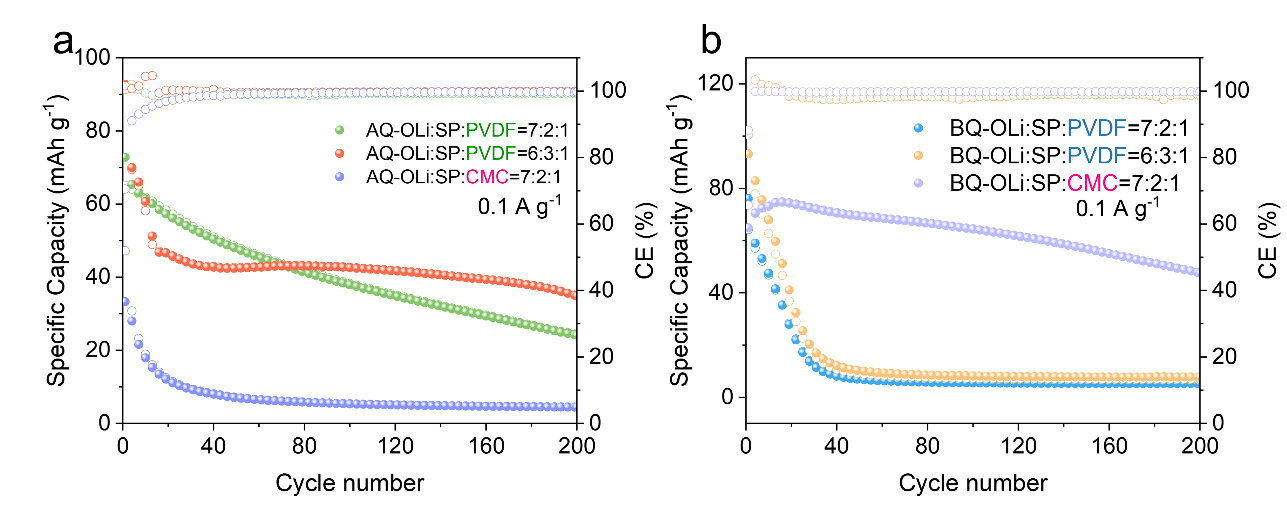


**Fig. S19** Cycling performance at 0.1 A g^-1^ of (**a**) AQ-OLi cathodes and (**b**) BQ-OLi cathodes with different coating ratios and binders

Although the binder also affects the electrochemical performance of AQ‑OLi and BQ‑OLi, both materials exhibited pronounced capacity decay with either CMC or PVDF due to the dissolution and instability of intermediate products (Fig. S19). It is worth noting that AQ‑OLi (PVDF) shows slightly better performance because AQ‑OLi has poor water solubility, resulting in a limited optimization effect from the dissolution‑reprecipitation process (Fig. S20), so the “dissolution-reprecipitation” process during electrode preparation has a limited effect on optimizing its electrode structure.


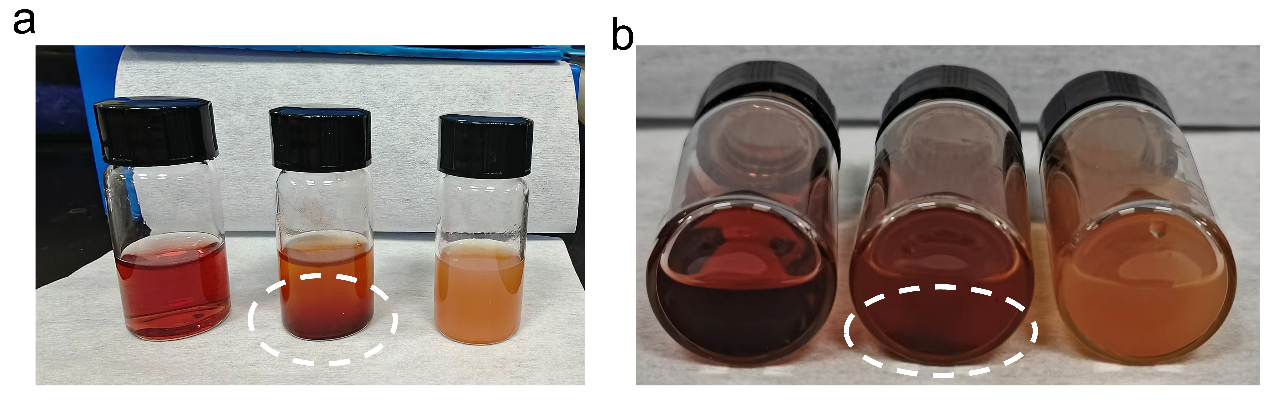


**Fig. S20** Optical images of the three materials dissolved in water (from left to right: BQ-OLi, AQ-OLi, NDI-OLi)

**
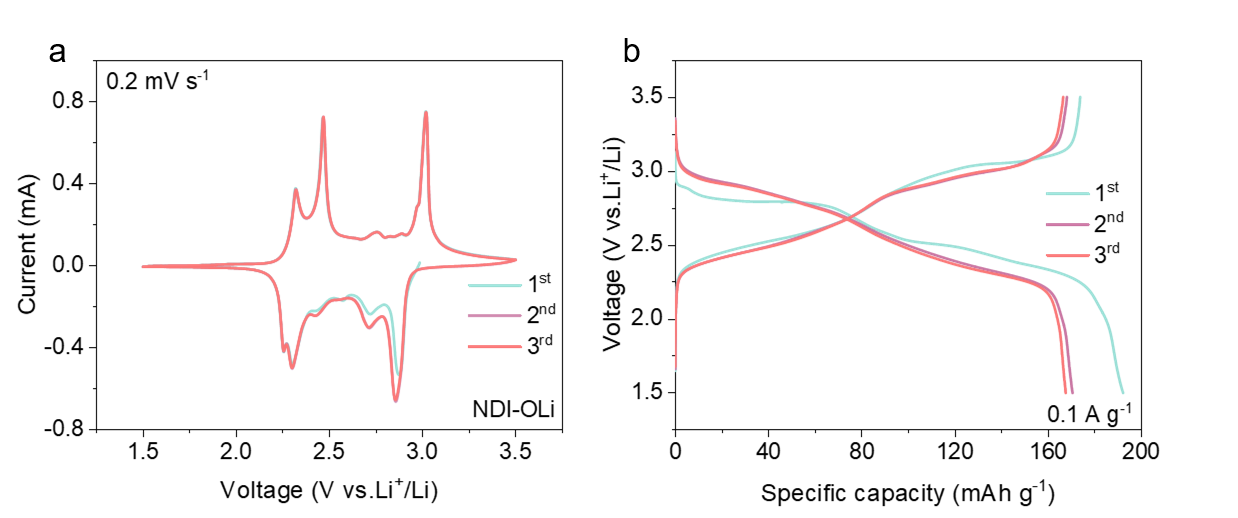
**

**Fig. S21 (a**) the CV curves at 0.2 mV s^-1^ and **b**) the discharge and charge profiles at 0.1 A g^-1^ of NDI-OLi (with CMC binder)


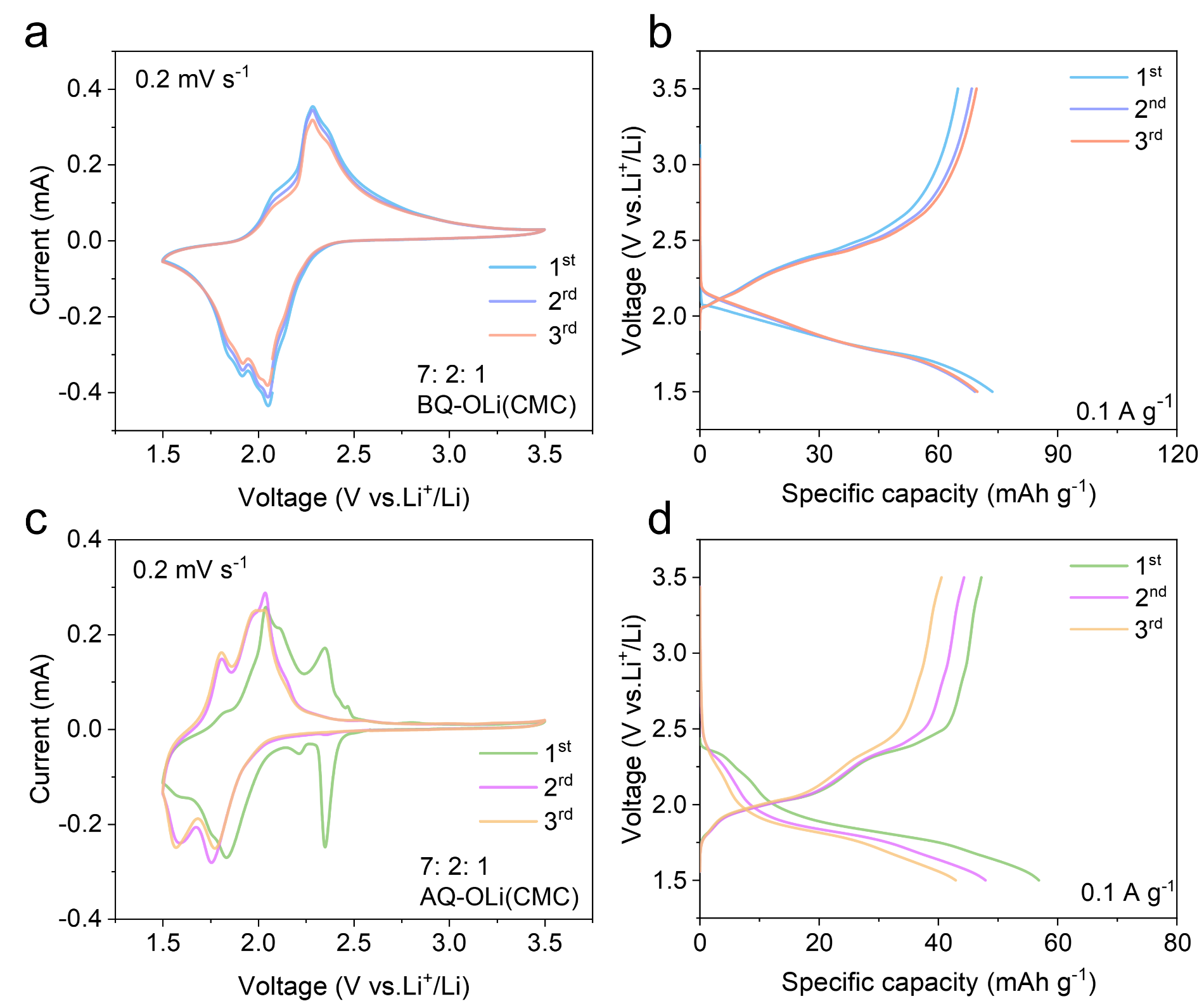


**Fig. S22** (**a, c**) the CV curves at 0.2 mV s^-1^ and **b, d**) the discharge and charge profiles at 0.1 A g^-1^ of BQ-OLi and AQ-OLi (with CMC binder)

**
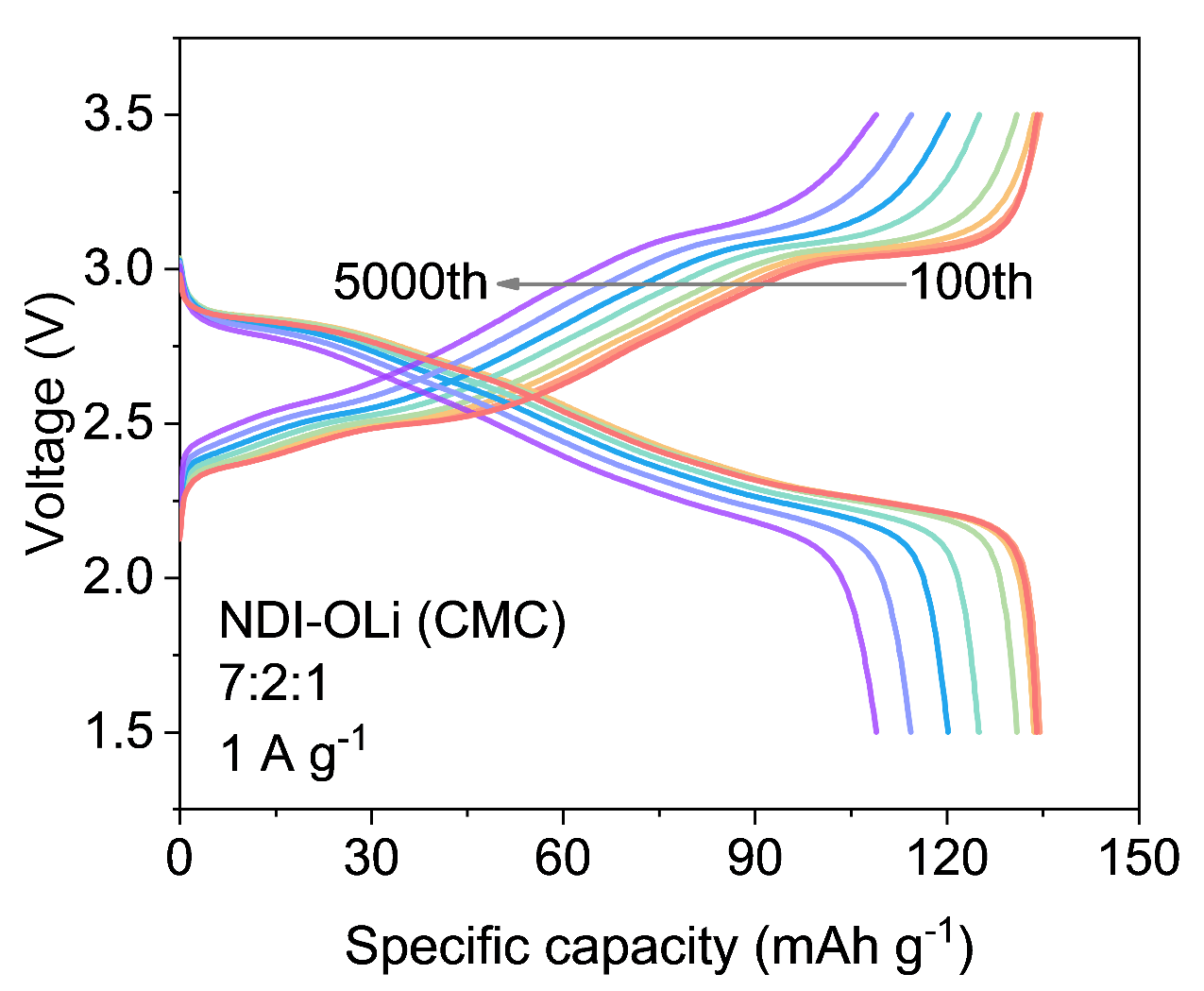
**

**Fig. S23** Charge-discharge curves of NDI-OLi cathode (with CMC binder) at the 100th, 200th, 500th, 1000th, 2000th, 3000th, 4000th and 5000th cycles under a current density of 1 A g^-1^

**
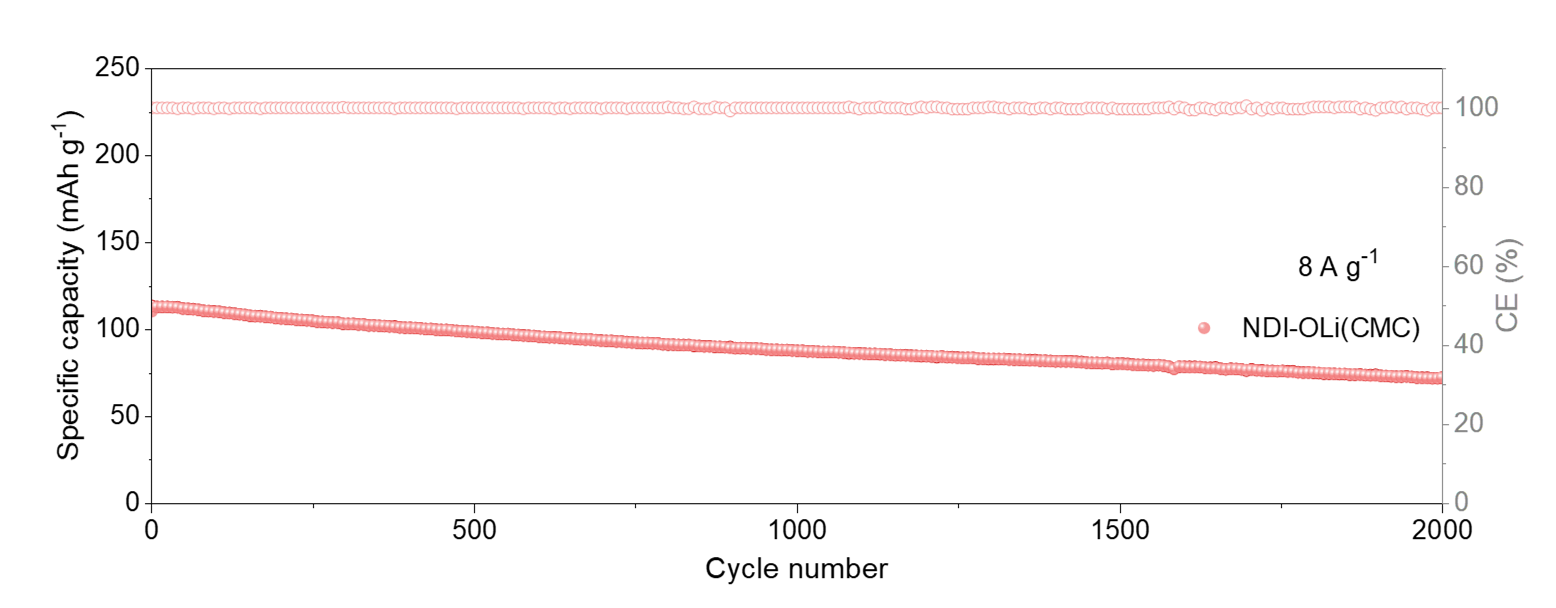
**

**Fig. S24** Long-term cycling stability of NDI-OLi cathode (with CMC binder) at 8 A g^-1^

**
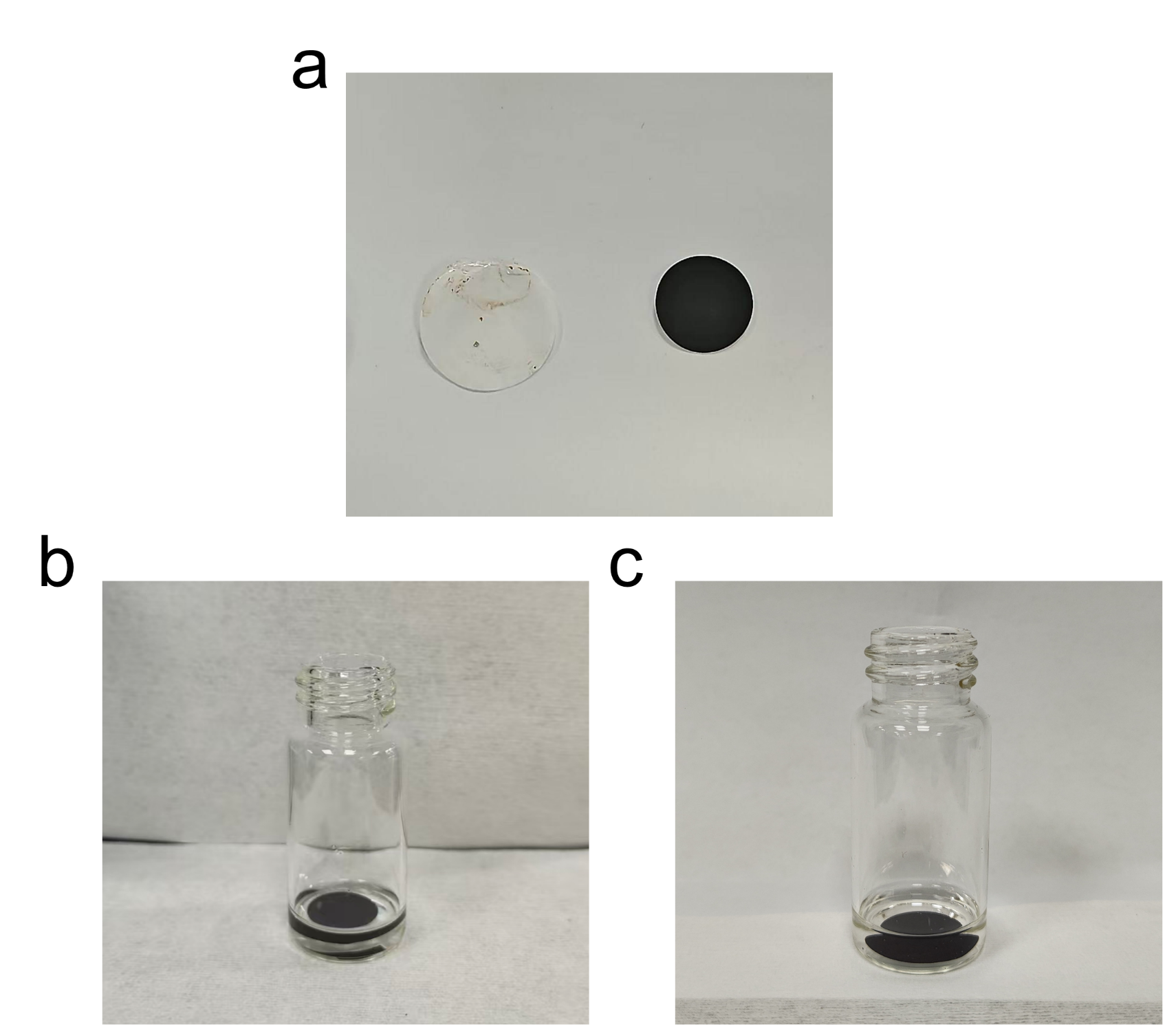
**

**Fig. S25** (**a**) Optical photographs of the cycled NDI-OLi electrode and the corresponding separator after 1000 cycles. (**b**) The cycled electrode immersed in electrolyte immediately after cell disassembly. (**c**) The cycled electrode after immersion in the electrolyte for 48 h.


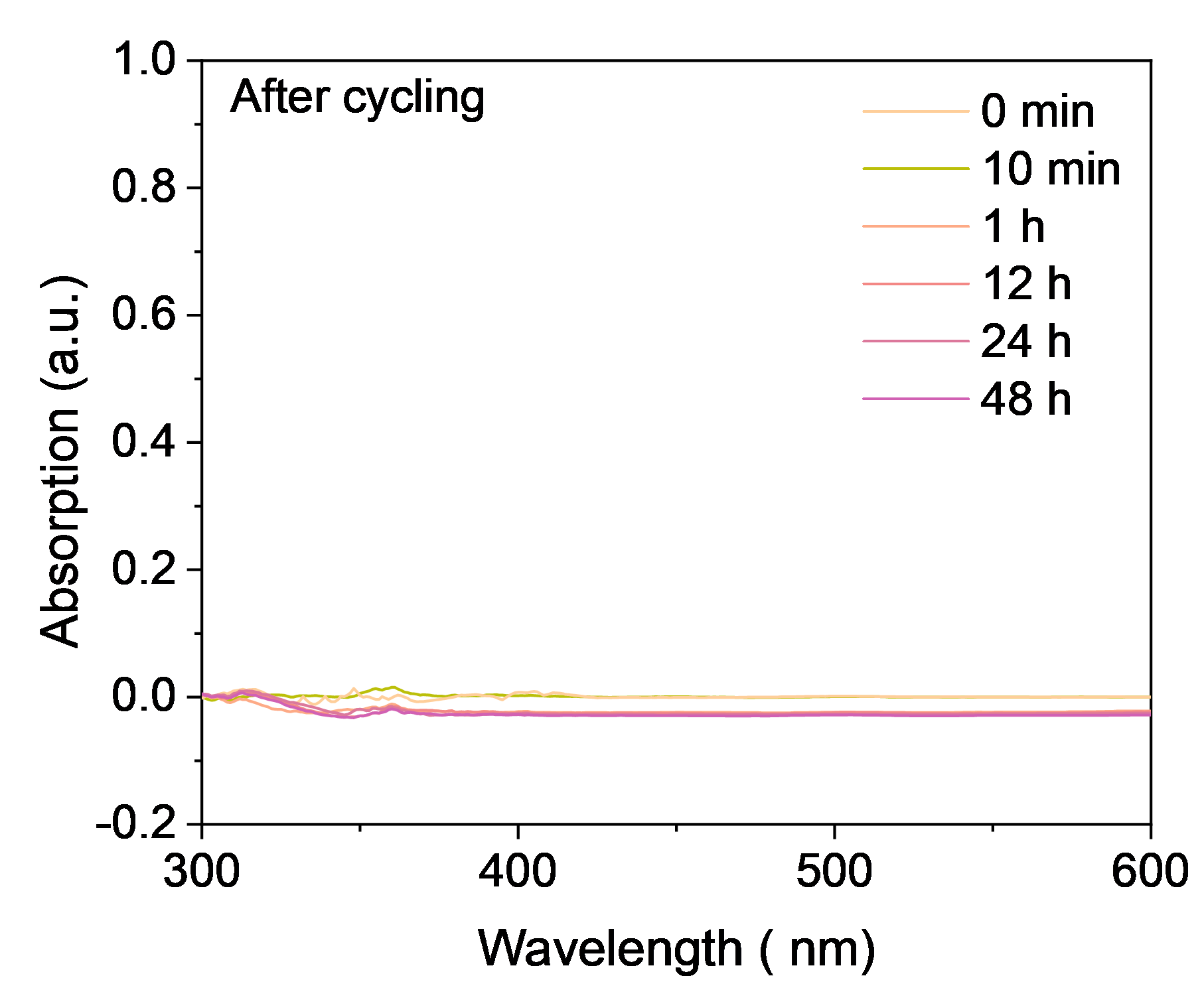


**Fig. S26** UV spectra of the NDI-OLi electrode (with an active material loading of 1.25 mg·cm⁻^2^) soaked in electrolyte after 1000 cycles at 8 A g⁻^1^

**
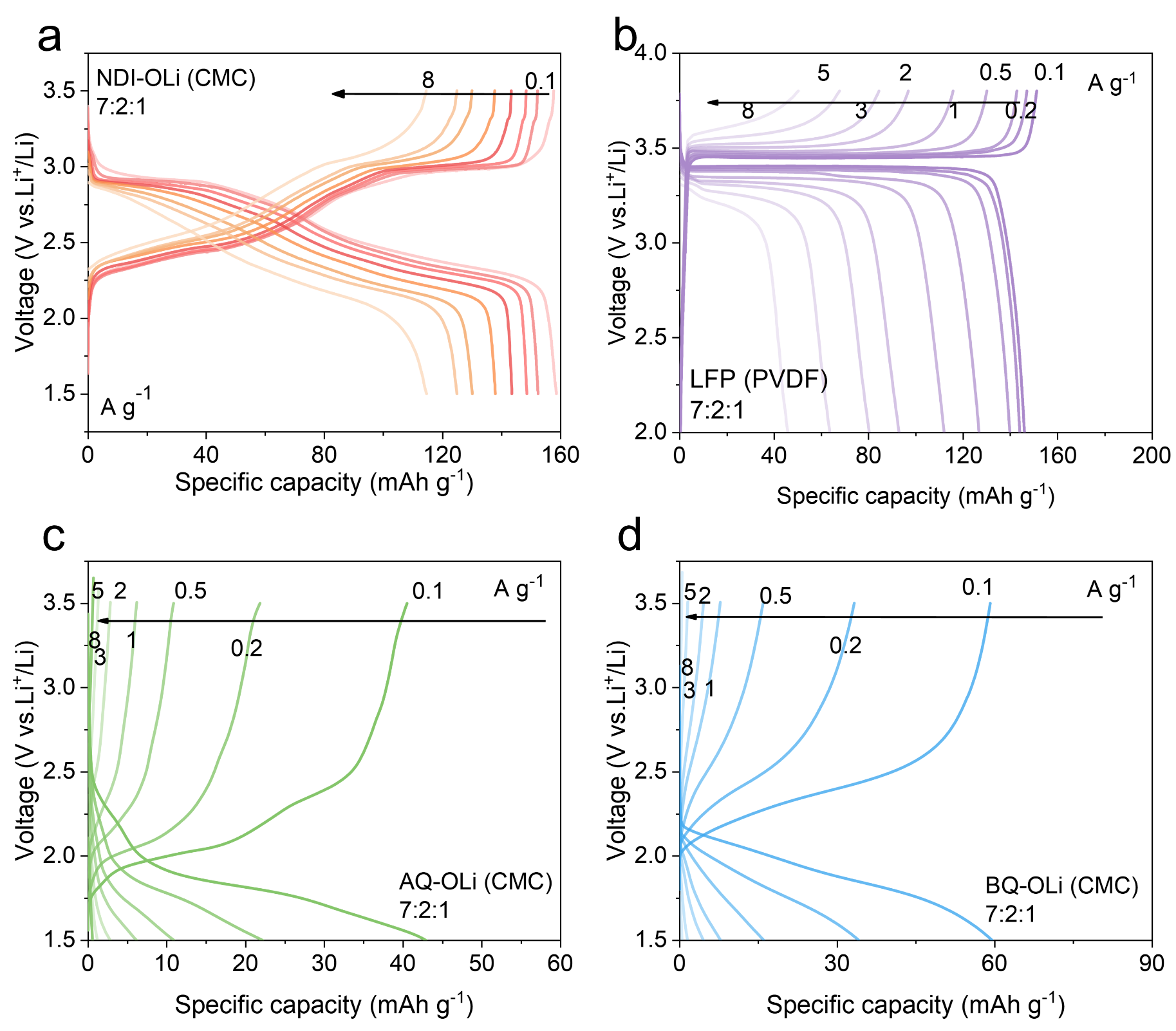
**

**Fig. S27** Rate capability and the discharge and charge profiles of (**a**) NDI-OLi, (**b**) LFP (with PVDF binder), (**c**) AQ-OLi and (d) BQ-OLi, respectively (with CMC binder)


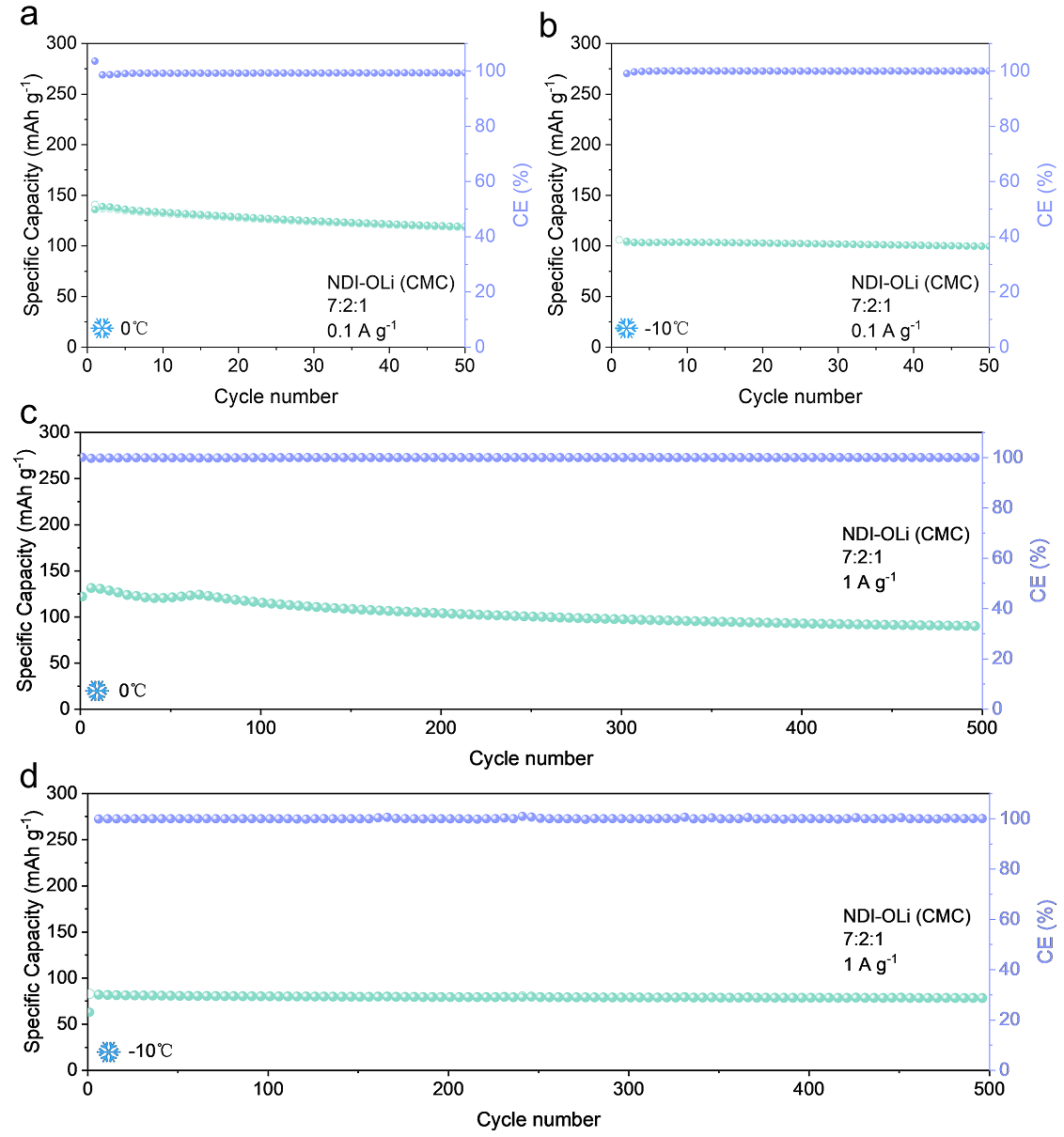


**Fig. S28** Cycling performance of NDI-OLi cathodes (with CMC binder) at (**a**) 0.1 A g^-1^ and (**c**) 1 A g^-1^ at 0 ℃ and at (**b**) 0.1 A g^-1^ and (**d**) 1 A g^-1^ at -10 ℃


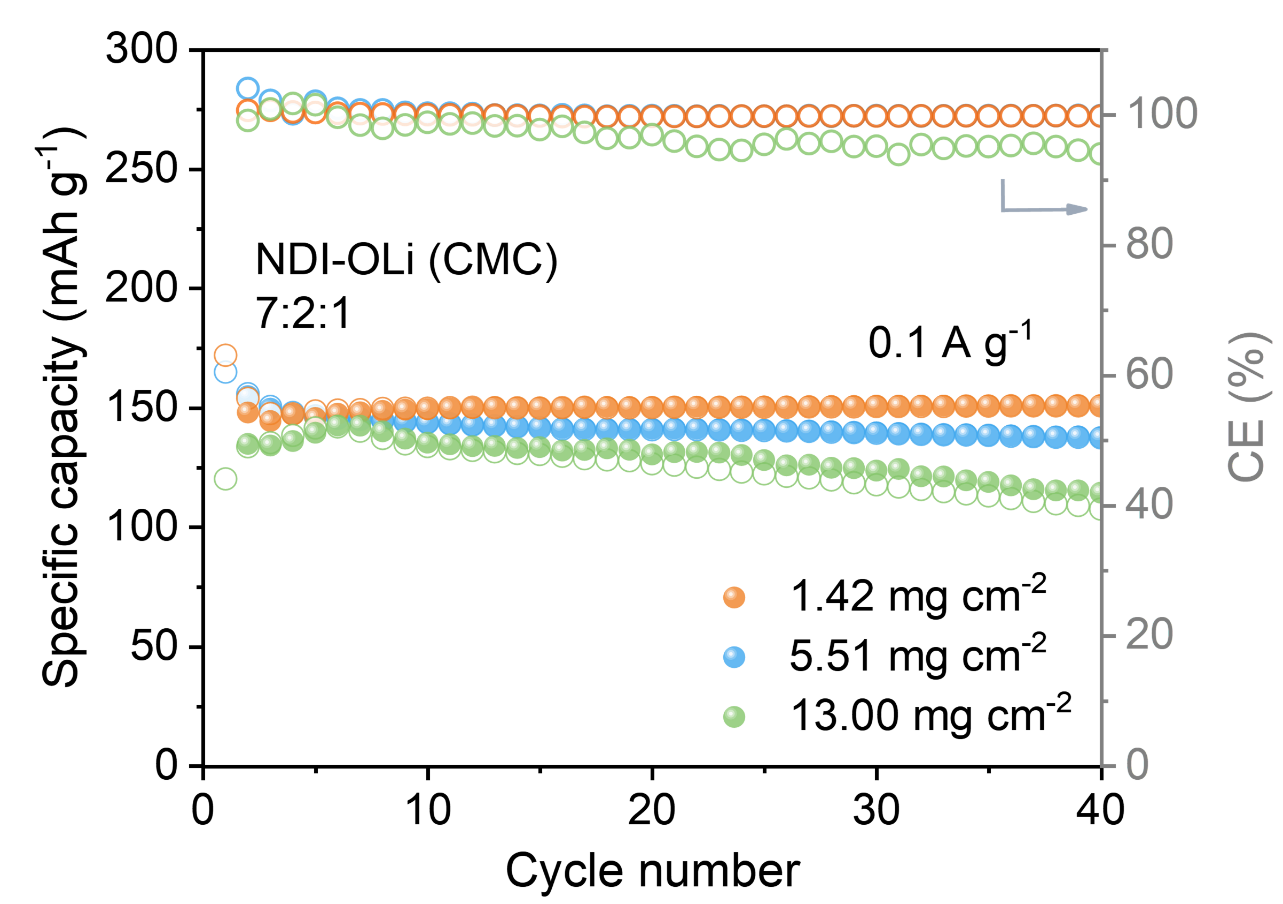


**Fig. S29** Cycling performance of NDI-OLi cathodes (with CMC binder) with different mass loading


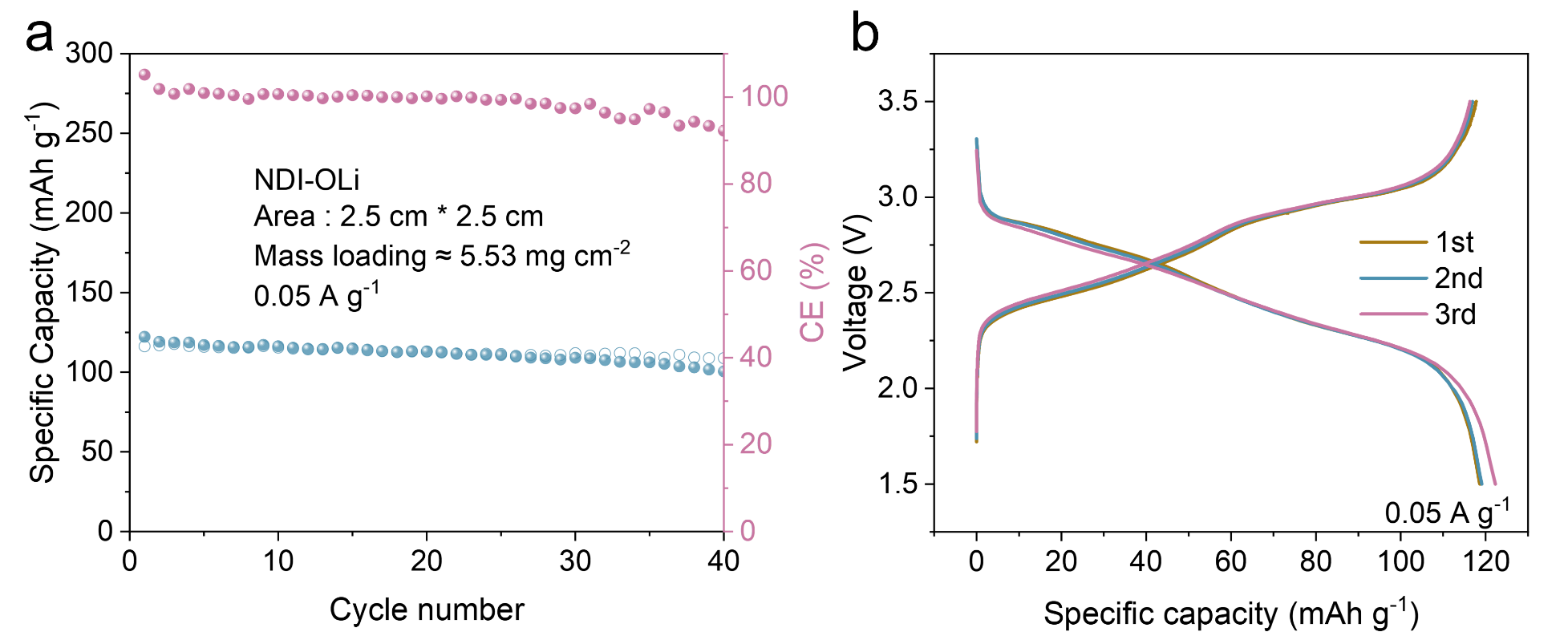


**Fig. S30** (**a**) Cycle performance of the NDI-OLi pouch cell. (**b**) The discharge and charge profiles at 0.1 A g^-1^ of the NDI-OLi pouch cell

**
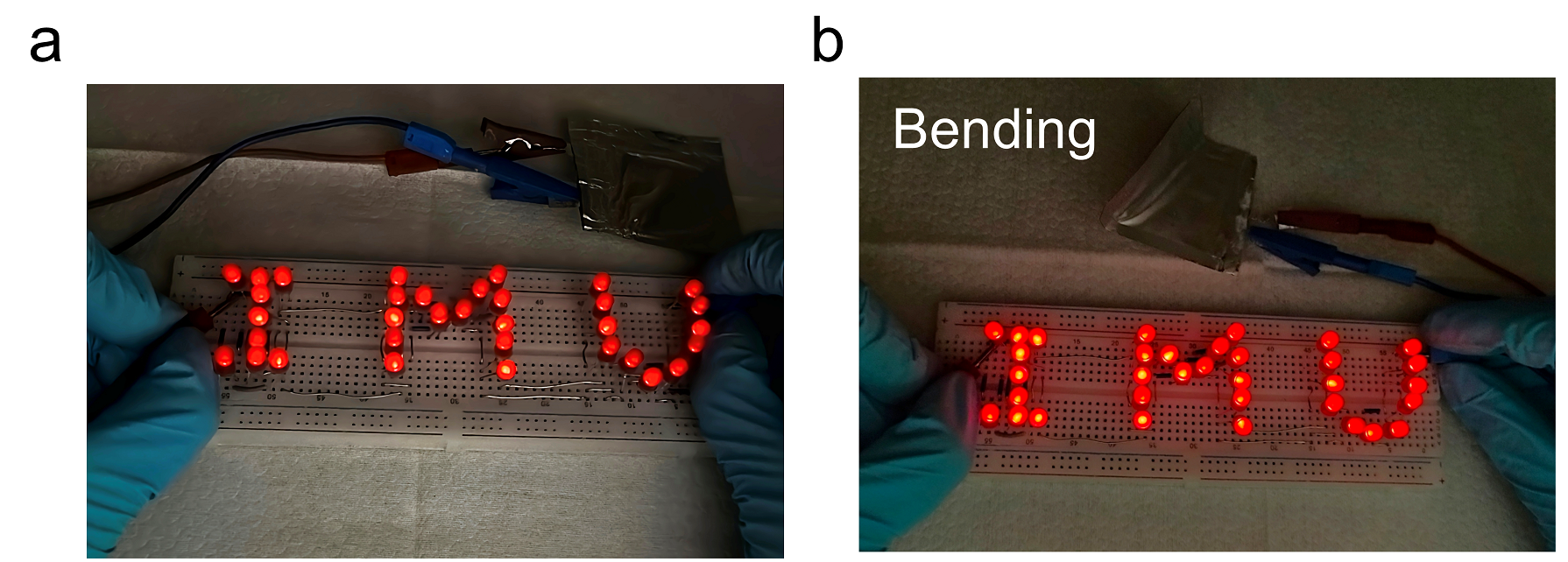
**

**Fig. S31** Optical image of an IMU pattern composed of 34 LED lights powered by NDI-OLi pouch cell **(a**) without bending and (**b**) under bending

**
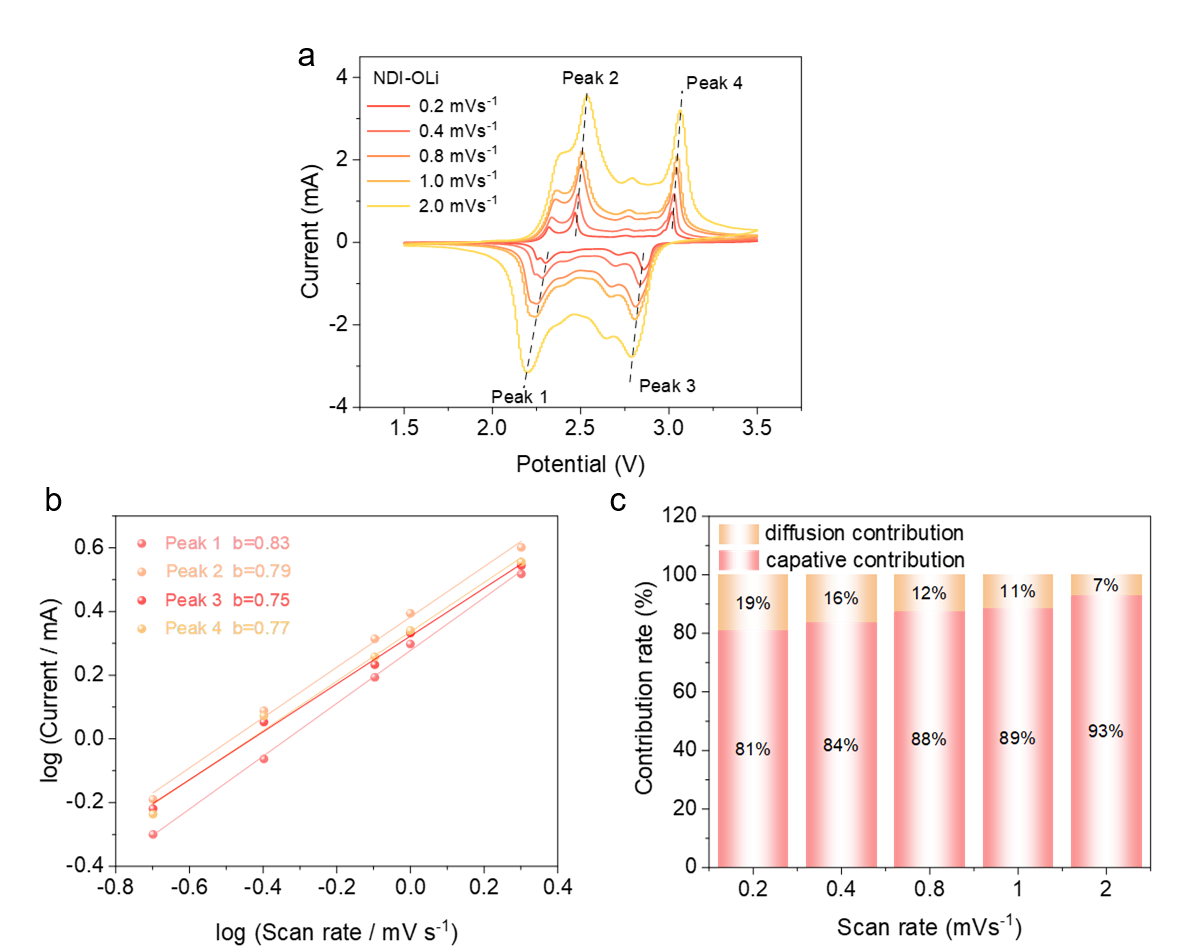
**

**Fig. S32** (**a**) CV curves of NDI-OLi cathode at different scan rates. (**b**) The relationship between logarithm redox peak current and logarithm scan rate for NDI-OLi cathode from CV curves. (**c**) The capacitive and diffusion contributions for NDI-OLi cathode at various scan rates

**
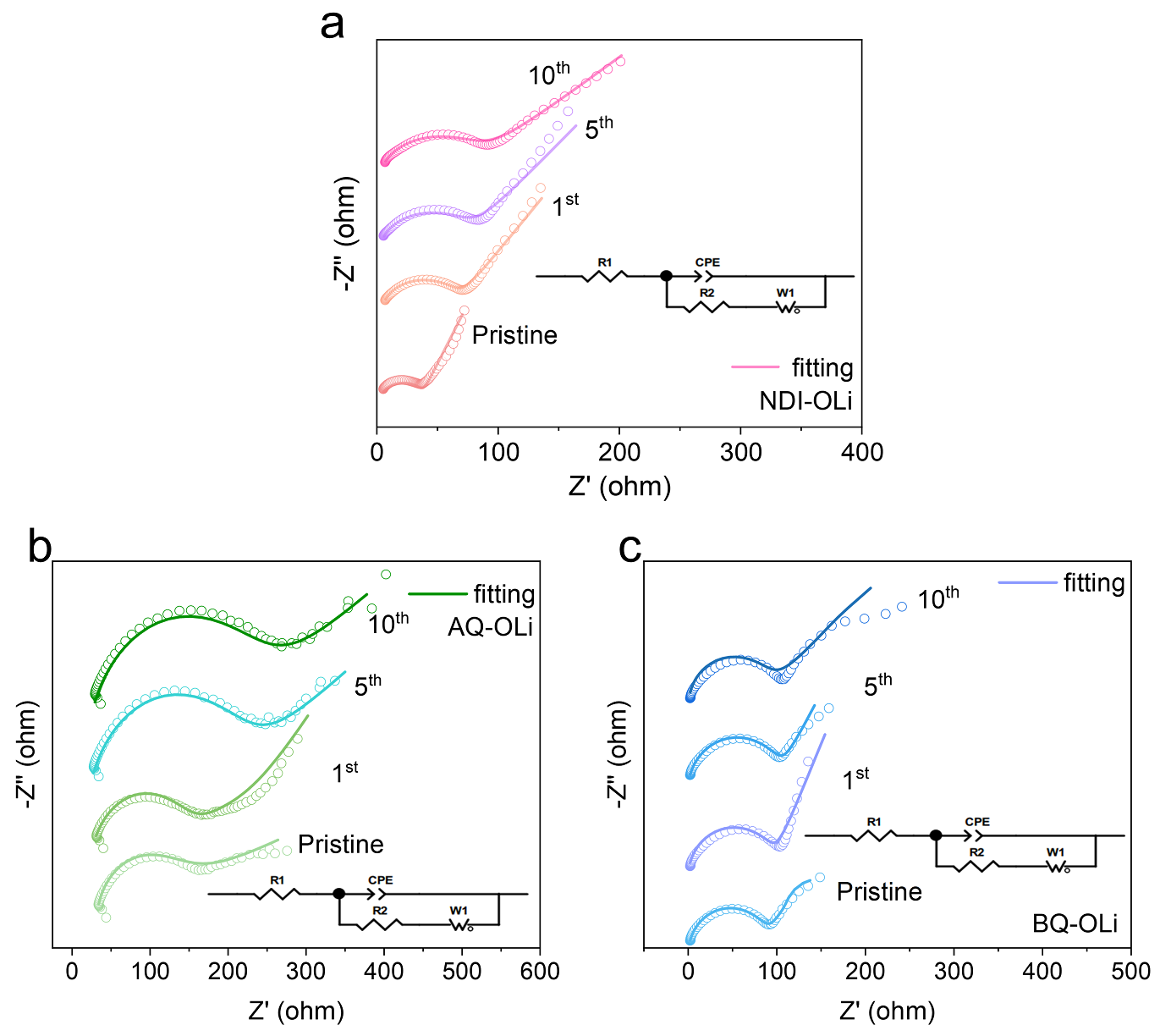
**

**Fig. S33** The Nyquist plots of (**a**) NDI-OLi, (**b**) AQ-OLi and (**c**) BQ-OLi


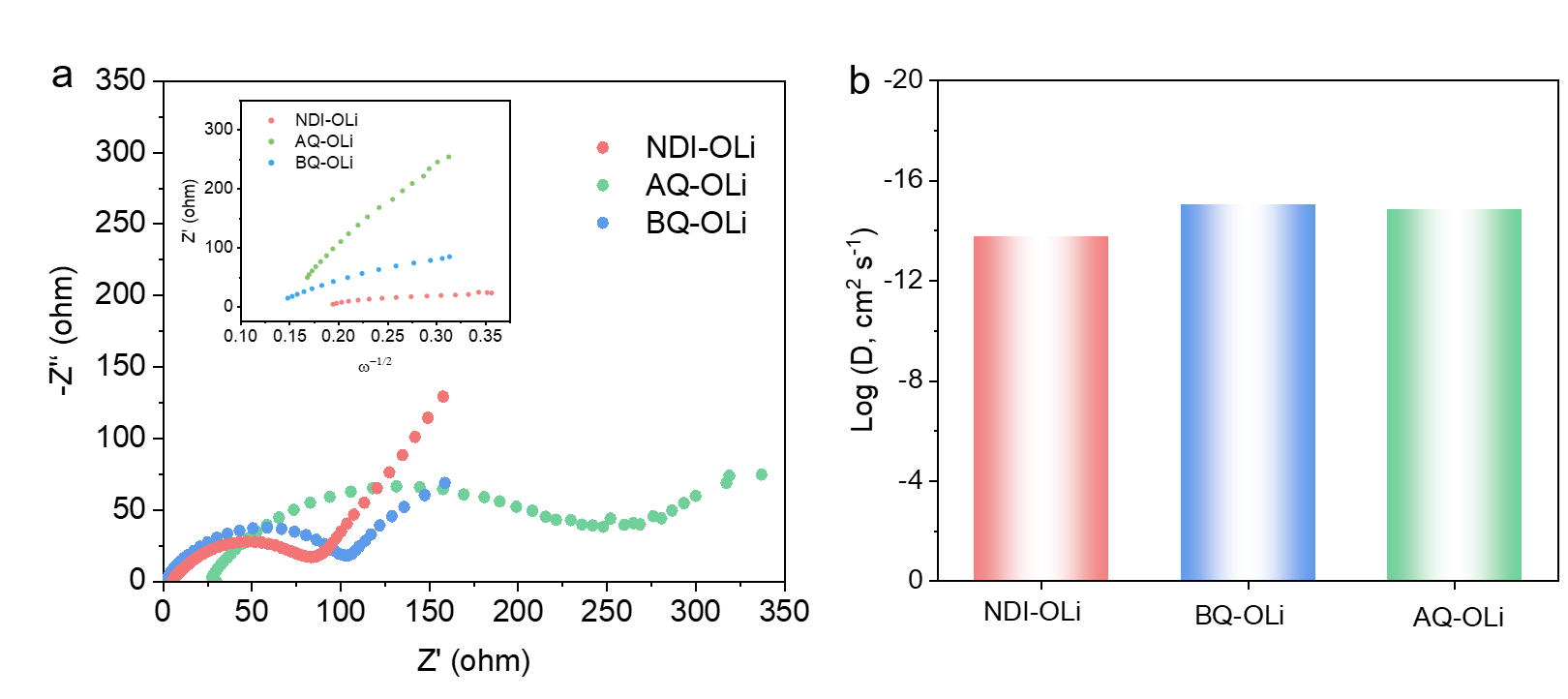


**Fig. S34** (**a**) Nyquist plots of NDI-OLi, AQ-OLi, and BQ-OLi electrodes. (**b**) Corresponding Li⁺ diffusion coefficients calculated from the EIS results for the three materials

**
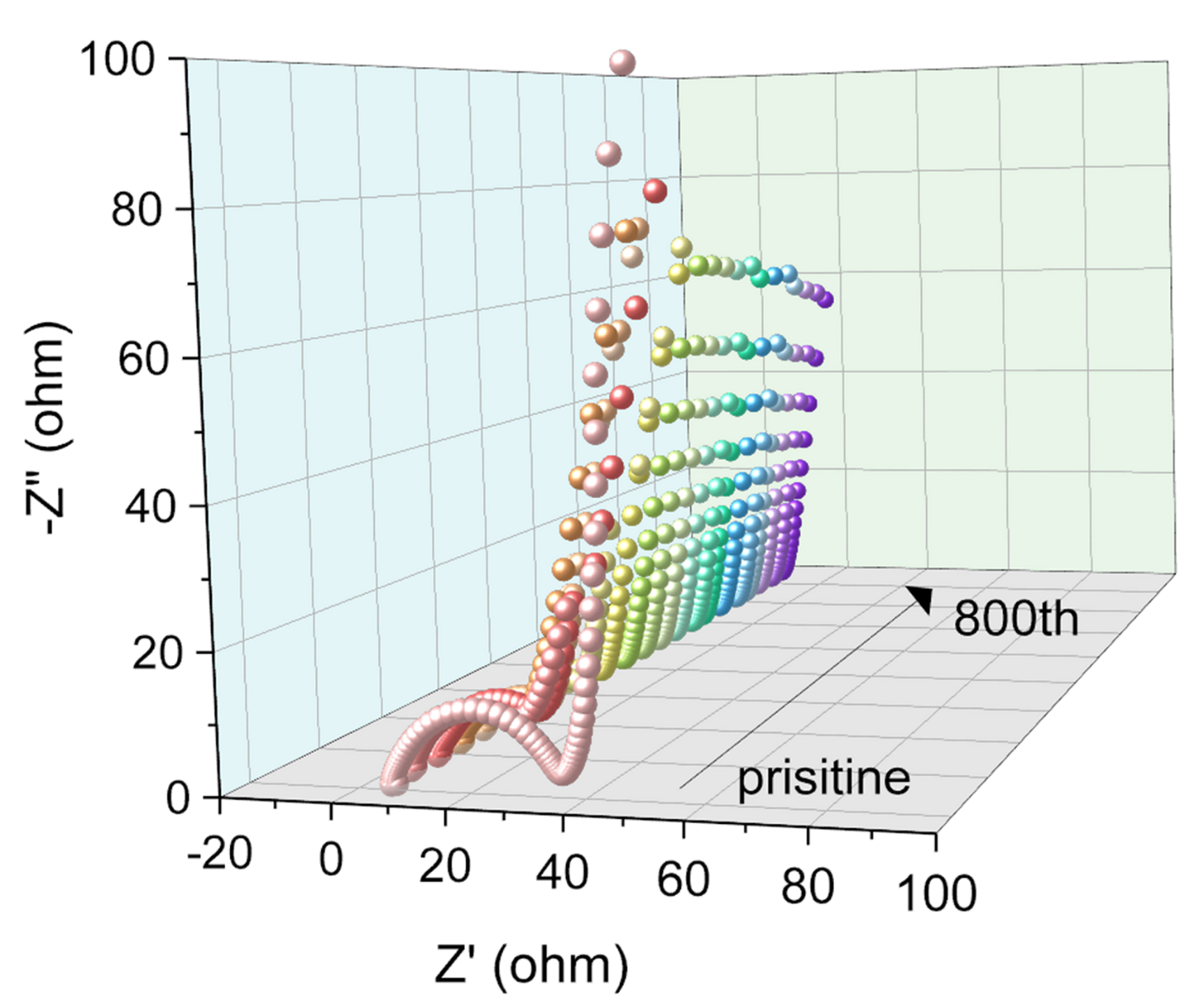
**

**Fig. S35** In-situ impedance of NDI-OLi electrode at different cycle numbers

**
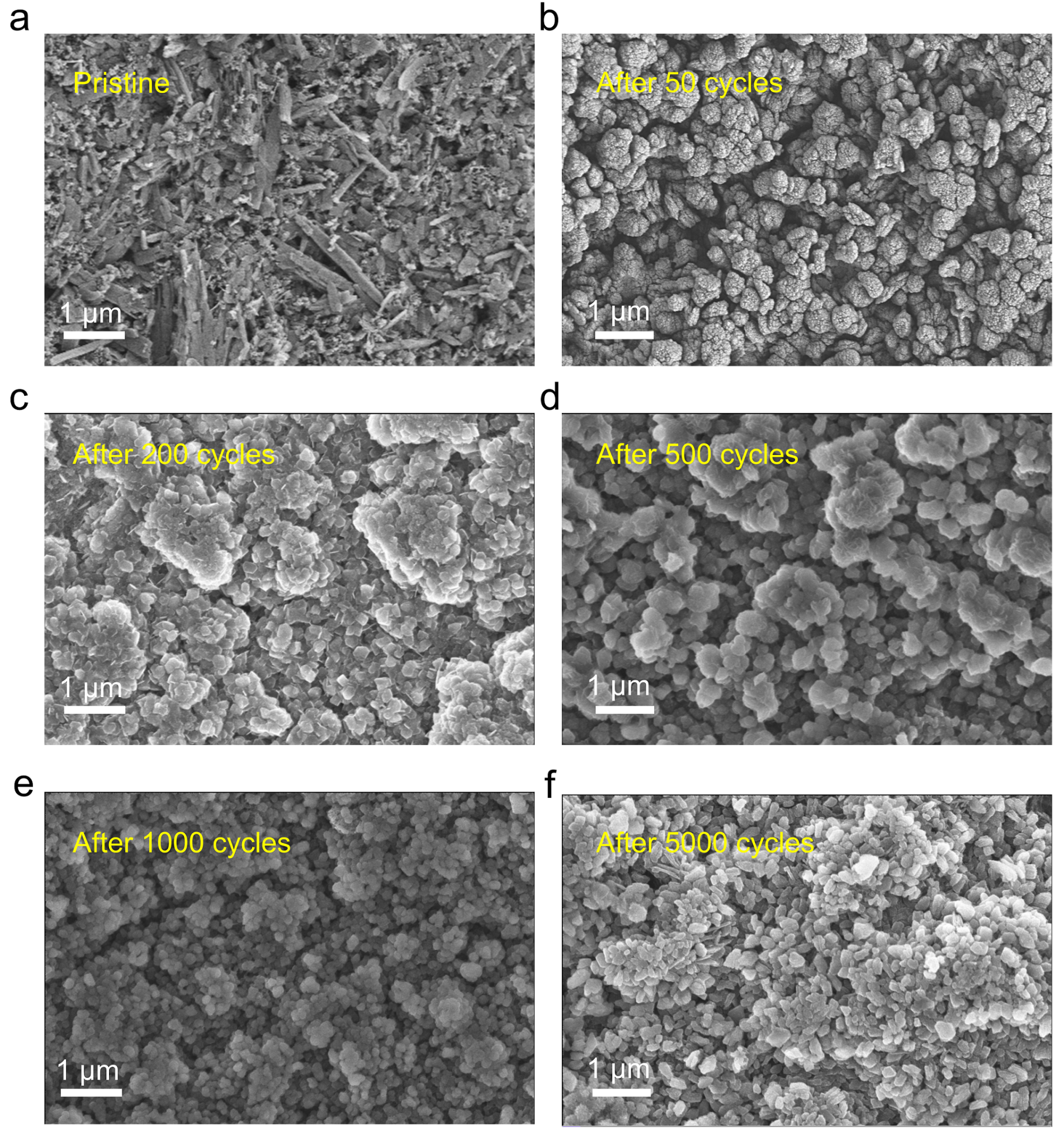
**

**Fig. S36** SEM images of NDI-OLi cathodes at different cycle numbers

**
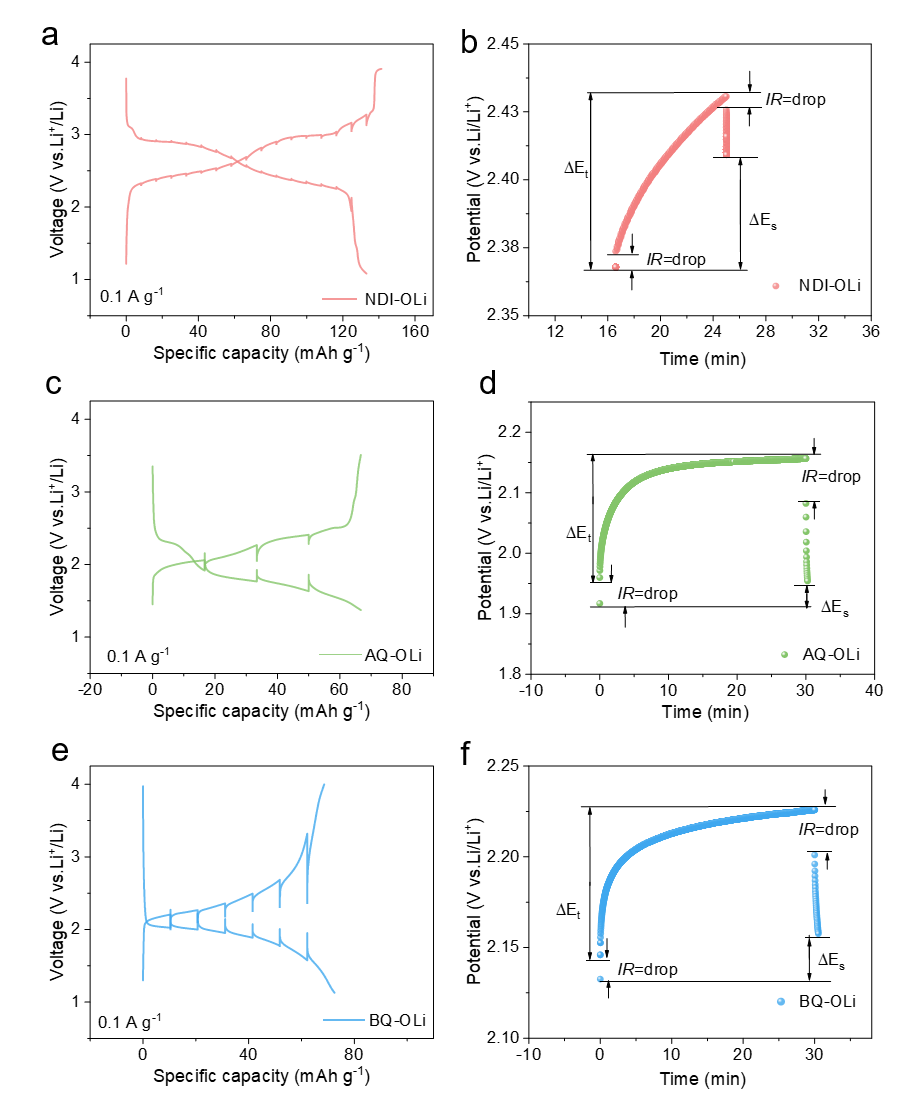
**

**Fig. S37** (**a, c, e**) The GITT curves of NDI-OLi , AQ-OLi and BQ-OLi. (**b, d, f**) GITT potential response curve with time for one typical charge step of NDI-OLi , AQ-OLi and BQ-OLi, respectively. The lower IR values further suggested that NDI-OLi exhibited faster electrochemical reaction kinetics

The Li^+^ ion diffusion coefficient (D_Li_^+^) was further calculated by according to the following equation:

$$D_{{Li}^{+}}=\left( 4/\pi\tau\right)\times\left( m_{a}V_{a}/M_{a}S \right)^{2}\times\left( {\Delta E}_{s}/{\Delta E}_{t} \right)^{2}$$

Where $\tau, m_{a}, V_{a}, M_{a} and S$ are the time of current pulse, mass loading, molar volume, molar mass and electrode-electrolyte interface area of the material, respectively. ${\Delta E}_{s}$ is the voltage difference between the initial state and steady state of each step during current pulse, and ${\Delta E}_{t}$ is the voltage change resulting from the current pulse excluding the IR drop.

**
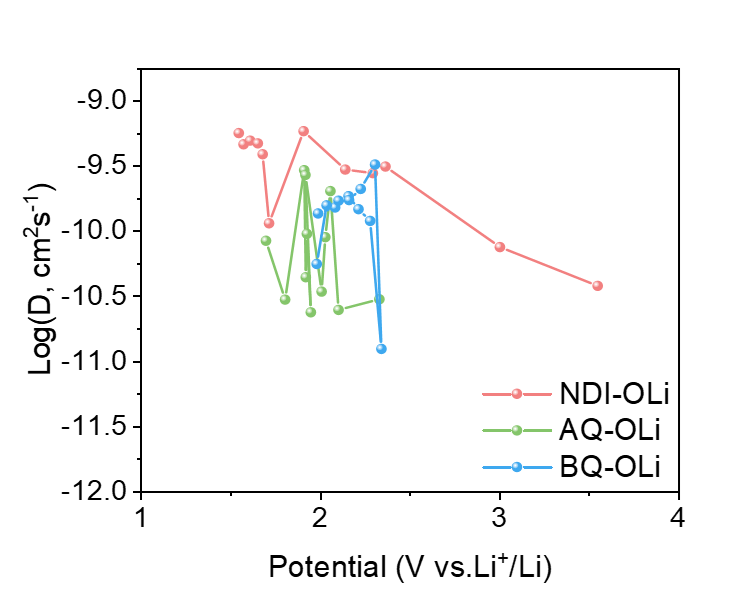
**

**Fig. S38** Diffusion coefficient of Li^+^ in NDI-OLi, AQ-OLi, BQ-OLi, respectively

The difference between the diffusion coefficients obtained from GITT and EIS may be attributed to the fact that GITT primarily reflects bulk diffusion, which is characterized by faster ion transport and lower energy barriers, whereas EIS is more sensitive to interfacial/surface diffusion, a process often associated with higher diffusion barriers and slower kinetics, thus yielding smaller diffusion coefficient values. Nevertheless, both methods consistently show that NDI-OLi possesses the highest lithium-ion diffusion coefficient, further supporting its excellent electrochemical kinetics.


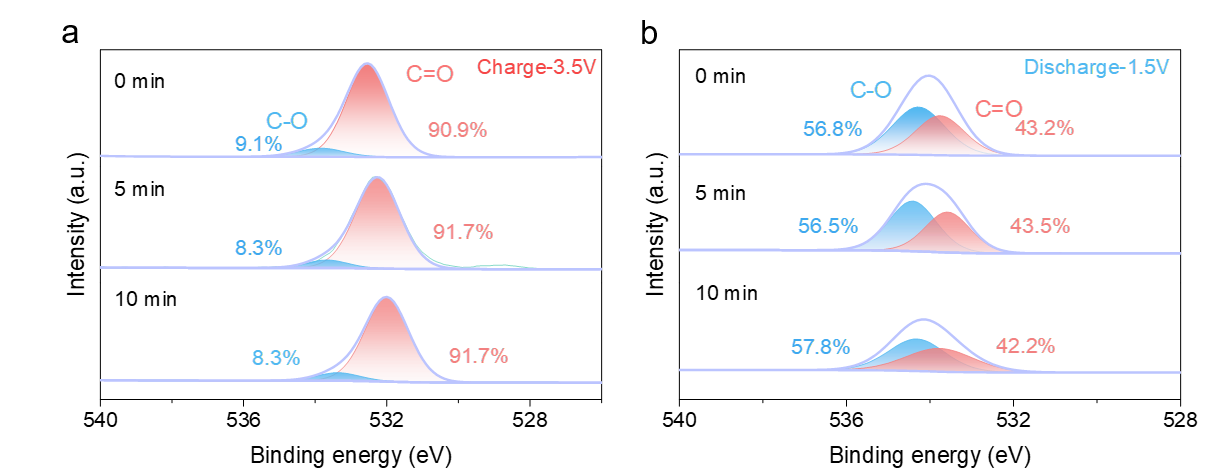


**Fig. S39** Ar⁺ deep sputtering O1s XPS of the NDI-OLi electrode after 5 cycles: (**a**) Fully charged and (**b**) fully discharged


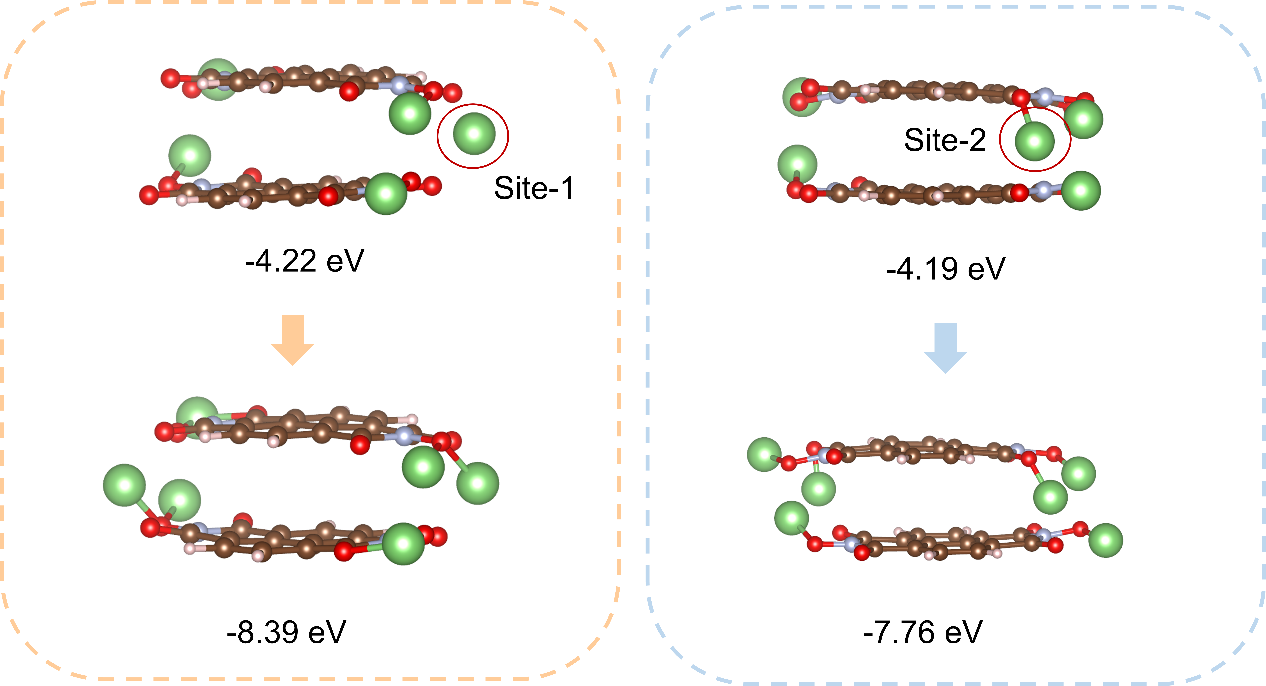


**Fig. S40** DFT calculations of the two possible Li⁺ insertion pathways in NDI-OLi during the first reduction step


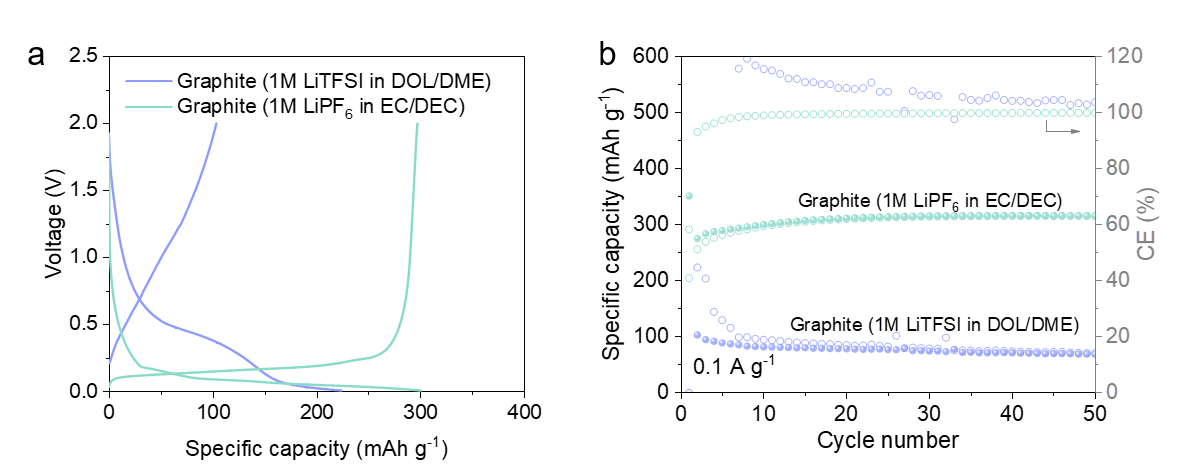


**Fig. S41** (**a**) Galvanostatic charge-discharge profiles and (**b**) cycling performance of graphite electrodes with different electrolytes


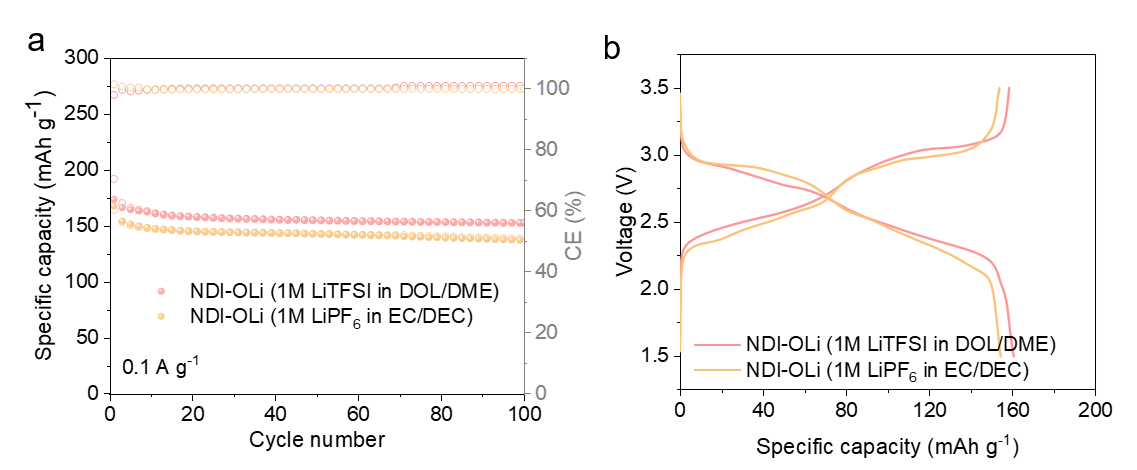


**Fig. S42** (**a**) Cycling performance at a current density of 0.1 A g⁻¹ and (**b**) galvanostatic charge–discharge profiles of NDI-OLi electrodes with different electrolytes


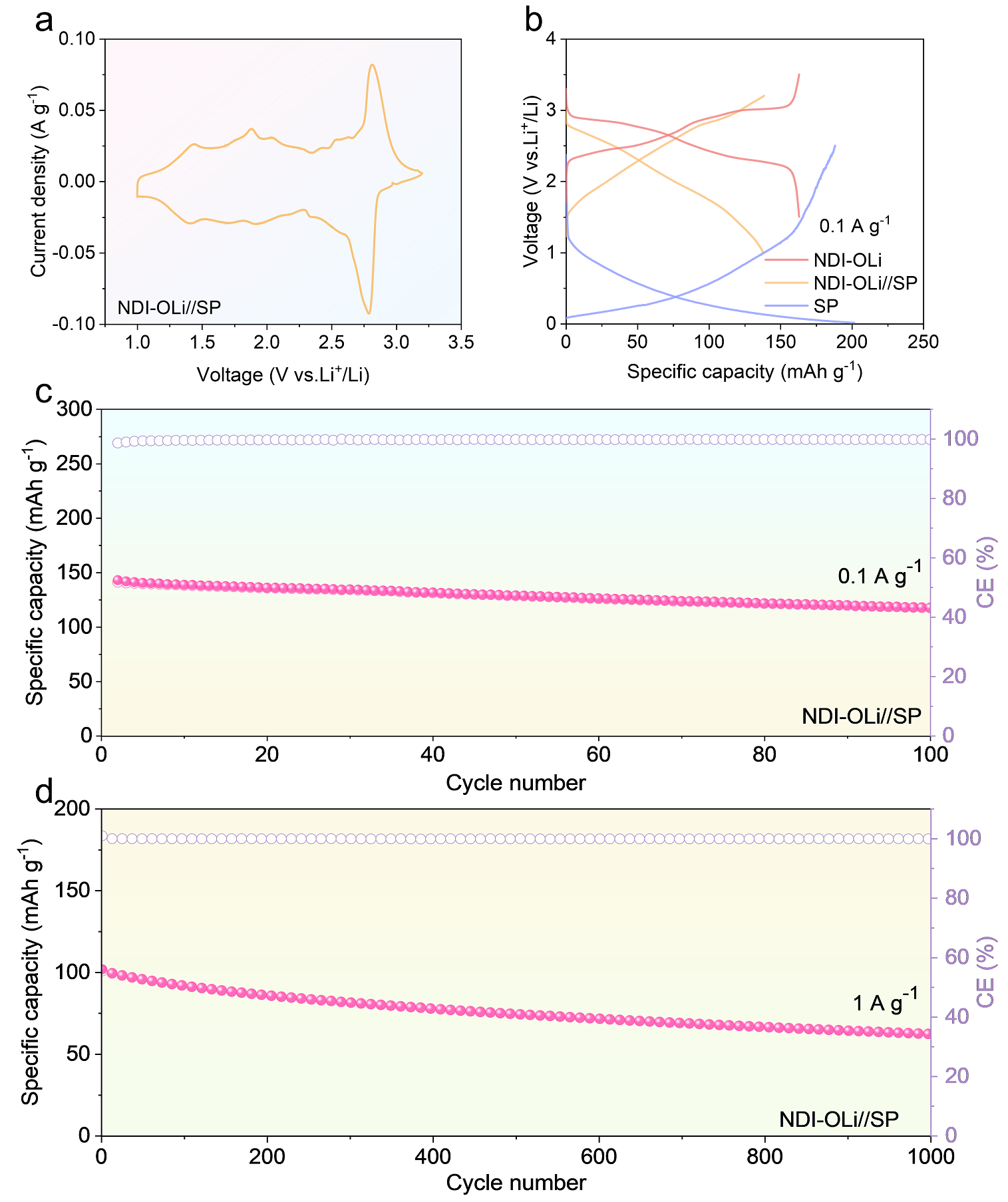


**Fig. S43** (**a**) CV curve of NDI-OLi//SP full cell at 0.2 mV s^-1^. (**b**) Charge-discharge curves of NDI-OLi//SP full cell, NDI-OLi//Li half-cell and SP//Li half-cell. Cycling performance of NDI-OLi//SP full cell at (**c**) 0.1 A g^-1^ (the mass of the active material is 1.25 mg cm^-2^) and (**d**) 1 A g^-1^ (the mass of the active material is 1.1 mg cm^-2^)

As shown in Fig. S43, the NDI-OLi//SP full cell still delivers a high discharge capacity of 140 mAh g^-1^ at 0.1 A g^-1^ and an average voltage greater than 2.1 V. The corresponding energy density is about 290 Wh kg^-1^ (calculated based on the cathode mass) and 155 Wh kg^-1^ (based on the total mass of cathode and anode). Furthermore, the full cell also shows good cycling stability, delivering a capacity of 120 mAh g^-1^ after 100 cycles, with 85% capacity retention (Fig. S43c). Even at a high current density of 1 A g^-1^, the full cell maintains outstanding cycling performance, achieving 62 mAh g^-1^ capacity after 1000 cycles with a 62% capacity retention rate (Fig. S43d).


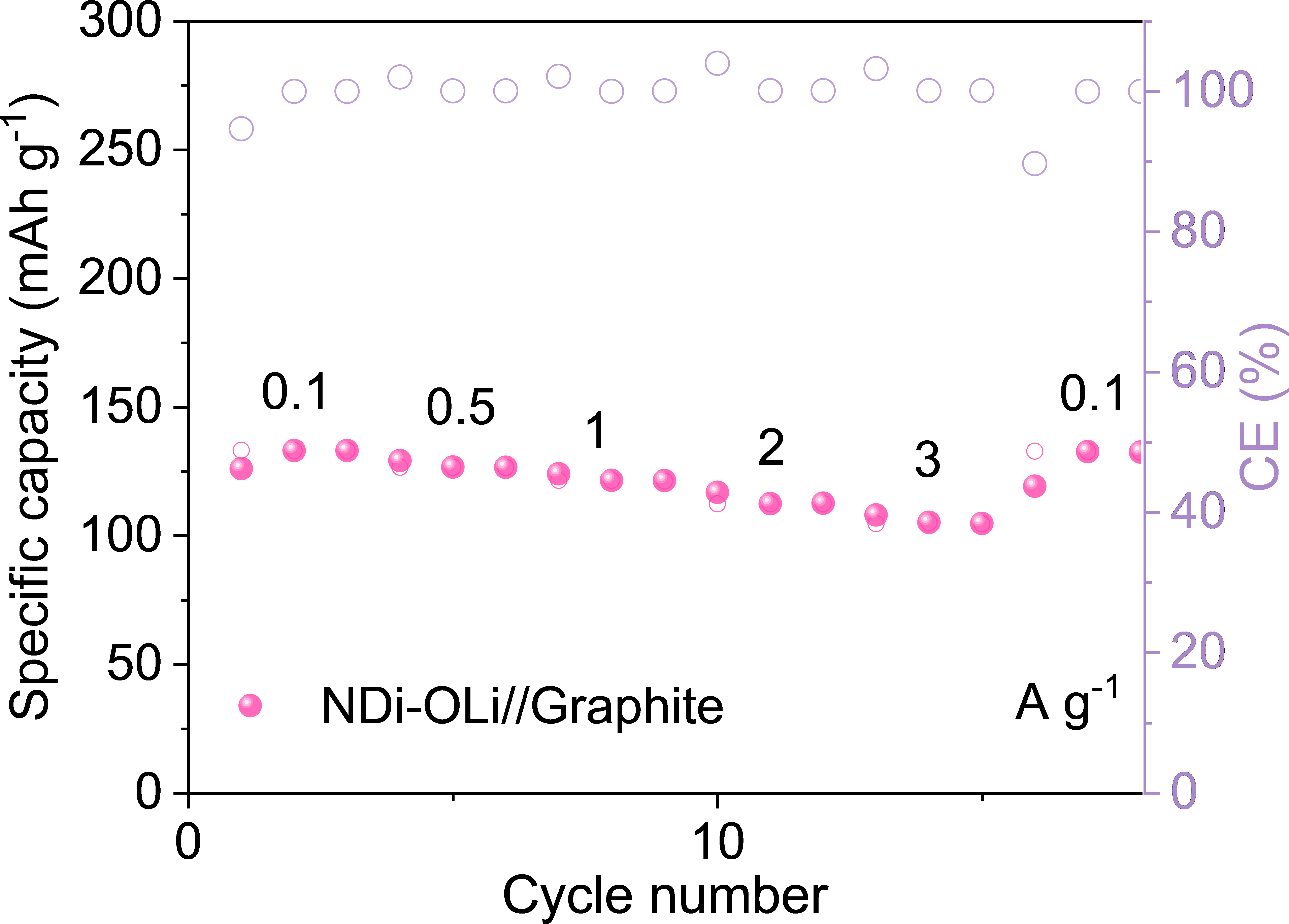


**Fig. S44** Rate capability of NDI-OLi//Graphite full cell

**Table S1** Electronic conductivity of NDI-OLi, AQ-OLi and BQ-OLi (four-probe method)

|  | ρ (g cm^-3^) | R (Ω) | σ (S m^-1^) |
| --- | --- | --- | --- |
| NDI-OLi | 1.50 | 1.10×10^6^ | 9.06×10^-7^ |
| AQ-OLi | 1.44 | 6.82×10^6^ | 1.47×10^-7^ |
| BQ-OLi | 1.28 | 1.16×10^9^ | 8.62×10^-10^ |

**Table S2** Summary of conductivity of common organic cathodes for LIB. The conductivity values were measured using the two-probe pressed pellet configuration unless otherwise noted

| Materials (reference) | Electrical  Conductivity of  active material  σ (S m^-1^) | Refs. |
| --- | --- | --- |
| NDI-OLi | 9.06×10^-7^ (four-probe) | **This work** |
|   Naphthalenetetracarbo xylic dianhydride (NTCDA) | 8.1×10^-8^ | [S5] |
|  Triquinoxalinylene (3Q) | 1.78×10^-11^ | [S6] |
|   Diquinoxalinylene (2Q) | 2.13×10^-12^ | [S6] |
|   1,4-Naphthoquinone (NQ) | 2.36×10^-9^ | [S7] |
|   Menadione | 8.98×10^-10^ | [S7] |
|   Polytriphenylamine (PTPAn) | 1×10^-4^ | [S8] |
|   Li_4_PTtSA | 5.1×10^-7^ | [S9] |
|   Phenazine (PNZ) | 2.8×10^-7^ | [S8] |
|   TAPT | 1.16×10^-6^ | [S9] |
|   Alloxazine (AX) | 4.8×10^-7^ | [S10] |
|   1,10-Phenanthroline- 5,6-dione (5,6-PhenQ) | 4.6×10^-7^ | [S10] |
|   Benzo[1,2-*b*:4,5-  *b*′]dithiophene-4,8- dione (BDTD) | 3.1×10^-7^ | [S11] |
|   Tetracyanoquinodimet hane (TCNQ) | 2.0×10^-7^ | [S12] |
|   Cu_3_(HHTN)_2_ | 9.01×10^-6^ | [S13] |
| Cu-HAOTP | 4.75×10^-7^ | [S14] |
| Cu-TPQG MOF | 2.39×10^-4^ | [S15] |
| CuSalphen MOF | 1.03×10^-4^ | [S16] |

**Table** **S3** Comparison of NDI-OLi cathode electrochemical properties and the other organic cathode materials reported in the literature for LIBs

| Materials (reference) | Electrode composition | Electrolyte | Average discharge/  cutoff voltage | Reversible/  theoretical capacity | Specific capacity (mAh g^-1^)  (Current density, A g^-1^) | Cycle Stability  Retention/ Cycles/ Current density | Ref. |
| --- | --- | --- | --- | --- | --- | --- | --- |
| NDI-OLi | 7:2:1 | 1 M LiTFSI  in DOL/DME | 2.5V/  1.5-3.5V | 163/173 mAh g^−1^ @ 100 mA g^−1^ | 155.6 mAh g^-1^ (0.1 A g^-1^)  101.9 mAh g^-1^ (8 A g^-1^) | 85%,5000,  1 A g^-1^ | **This work** |
|   Na₂BNDI | 7:3 | 1 M LiPF_6_  in EC/DMC | 2.5V/  1.5-4V | 147/125 mAh g^−1^  @ 25 mA g^−1^ | — | 93.6%,50,  0.025 A g^-1^ | [S17] |
|   Na₂BESANDI | 7:3 | 1 M LiPF_6_  in EC/DMC | 2.3V/  1.5-4V | 113/102 mAh g^−1^  @ 20.4 mA g^−1^ | — | 98.3%,50,  0.0204 A g^-1^ | [S17] |
|   Li_2_NTCDI | 6:3:1 | 1 M LiTFSI  in DOL/DME | 2.5 V/  1.5-3.5V | 131/207.7 mAh g^−1^  @ 65.5 mA g^−1^ | 131 mAh g^-1^ (0.013 A g^-1^)  130 mAh g^-1^ (0.026 A g^-1^)  128 mAh g^-1^ (0.065 A g^-1^)  125 mAh g^-1^ (0.131A g^-1^)  117 mAh g^-1^ (0.262 A g^-1^) | 98.3%,100,  0.0655 A g^-1^ | [S18] |
| Li_2_C_5_O_5_ | 6:3:1 | 1 M LiPF_6_  in DME | 0.8V  0.2-3V | 170/348 mAh g^−1^ @ 70 mA g^−1^ | 285.4 mAh g^-1^ (0.17 A g^-1^)  191.4 mAh g^-1^ (0.35 A g^-1^)  180.9 mAh g^-1^ (0.70 A g^-1^)  34.8 mAh g^-1^ (1.74 A g^-1^) | 103%,10,  0.07 A g^-1^ | [S19] |
| Li_2_C_6_H_2_O_4_∙2H_2_O | 55:35:10 | 1 M LiPF_6_  in EC/DMC | 1.9 V/  1.5−3.5 V | 178/285 mAh g^−1^ @ 100 mA g^−1^ | 134 mAh g^-1^ (0.1 A g^-1^) | 77%,10,  0.1 A g^-1^ | [S20] |
| Li_2_C_6_O_6_ | 85:15 | 1 M LiPF_6_  in EC/DMC | 2.1 V/  1.5−3.5 V | 580/589 mAh g^−1^ @ 15 mA g^−1^ | — | 46%,14,  0.015 A g^-1^ | [S21] |
| Li_4_C_6_O_6_ | 8:2 | 1 M LiPF_6_  in EC/DMC | 1.8 V/  1.5−2.5 V | 200/274 mAh g^−1^ @ 14 mA g^−1^ | 250 mAh g^-1^ (0.01 A g^-1^)  248 mAh g^-1^ (0.02 A g^-1^)  220 mAh g^-1^ (0.27 A g^-1^)  160 mAh g^-1^ (0.82 A g^-1^) | 90%,50,  0.014 A g^-1^ | [S22] |
| Li_2_C_8_H_6_O_6_ | 56.6:33.3:10 | 1 M LiPF_6_  in EC/DMC | 2.0 V/  2.15−3.6 V | 138/253 mAh g^−1^ @ 63 mA g^−1^ | 130 mAh g^-1^ (0.8 A g^-1^) | 58%,50,  0.063 A g^-1^ | [S23] |
| Li_2_C_6_O_4_Cl_2_ | 3:1 | 1 M LiPF_6_  in EC/DMC | 2.3 V/  1.35−3.0 V | 200/243 mAh g^−1^ @ 12 mA g^−1^ | — | 50%,25,  0.012 A g^-1^ | [S24] |
|   Li_2_C_8_H_2_O_6_@GO | 6:3:1 | 1 M LiPF_6_  in EC/DEC | 2.6 V/  1.8−3.2 V | 241/241 mAh g^−1^ @ 24 mA g^−1^ | 241 mAh g^-1^ (0.02 A g^-1^)  230 mAh g^-1^ (0.1 A g^-1^)  175 mAh g^-1^ (1 A g^-1^)  140 mAh g^-1^ (2 A g^-1^) | 84%,1000,  0.48 A g^-1^ | [S25] |
| BP_4_OLi | 4:5:1 | 2 M LiTFSI  in DOL/DME | 2.75V/  1.5-3.75V | 432/443 mAh g^−1^ @ 50 mA g^−1^ | 348 mAh g^-1^ (0.05 A g^-1^)  298 mAh g^-1^ (0.1 A g^-1^)  250 mAh g^-1^ (0.2 A g^-1^)  225 mAh g^-1^ (0.3 A g^-1^)  210 mAh g^-1^ (0.4 A g^-1^) | 53%,600,  0.5 A g^-1^ | [S26] |
| Li_2_DHNQ | 4:5:1 | 2 M LiTFSI  in G4 | 2.2 V/  1.2−4.0 V | 259/265 mAh g^−1^ @ 20 mA g^−1^ | — | 22%,20,  0.02 A g^-1^ | [S27] |
| Li_2_-1,5-DHAQ | 80:15:5 | 1 M LiPF_6_  in EC/DMC/EMC | 1.8 V/  1.0−3.8 V | 126/213 mAh g^−1^ @ 22 mA g^−1^ | 128 mAh g^-1^ (0.022 A g^-1^)  108 mAh g^-1^ (0.111 A g^-1^) | 95%,50,  0.022 A g^-1^ | [S28] |
| Li_2_BHNQ | 6:4 | 1 M LiTFSI  in G4 | 2.2 V/  0.7−3.0 V | 90/300 mAh g^−1^ @ 50 mA g^−1^ | 130 mAh g^-1^ (0.02 A g^-1^)  115 mAh g^-1^ (0.05 A g^-1^)  90 mAh g^-1^ (0.1 A g^-1^)  78 mAh g^-1^ (0.2 A g^-1^) | 33%,35,  0.05 A g^-1^ | [S29] |
| Li_4_THDTPT | 4:5:1 | 2.4 M LiTFSI  in Sulfolane | 2.7 V/  1.5−4.0 V | 420/462 mAh g^−1^ @ 50 mA g^−1^ | 420 mAh g^-1^ (0.02 A g^-1^)  380 mAh g^-1^ (0.1 A g^-1^)  300 mAh g^-1^ (0.4 A g^-1^)  200 mAh g^-1^ (0.8 A g^-1^) | 71%,100,  0.05 A g-1 | [S30] |
|   Li_2_-PDCA | 5:4:1 | 1 M LiPF₆ in EC/DMC | 2.75 V/2.2-3.3V | 160/320mAh g^−1^ @ 64 mA g^−1^ | 150 mAh g^-1^ (0.016 A g^-1^)  140 mAh g^-1^ (0.032 A g^-1^)  130 mAh g^-1^ (0.32 A g^-1^)  100 mAh g^-1^ (0.16 A g^-1^)  50 mAh g^-1^ (0.32 A g^-1^)  11 mAh g^-1^ (0.8 A g^-1^)  5 mAh g^-1^ (1.6 A g^-1^) | 80%,700,  0.16 A g^-1^ | [S31] |
| Li_4_- PTFSA | 5:4:1 | 1 M LiPF₆ in EC/DMC | 3.2V/2.8-3.6 | 78/78mAh g^−1^ @ 15.6 mA g^−1^ | 75 mAh g^-1^ (0.032 A g^-1^)  50 mAh g^-1^ (0.08 A g^-1^)  37.5 mAh g^-1^ (0.16 A g^-1^)  27 mAh g^-1^ (0.32 A g^-1^)  25 mAh g^-1^ (0.8 A g^-1^)  15 mAh g^-1^ (1.6 A g^-1^) | 76%,50,  0.032 A g^-1^ | [S31] |
| Na_2_C_6_O_6_ | 6:3:1 | 0.6 M NaPF_6_  in G2 | 1.6 V/  0.5−3.2 V | 498/501mAh g^−1^ @ 50 mA g^−1^ | 498 mAh g^-1^ (0.05 A g^-1^)  440 mAh g^-1^ (0.25 A g^-1^)  405 mAh g^-1^ (0.5 A g^-1^)  370 mAh g^-1^ (1.0 A g^-1^) | 91%,50,  0.5 A g^-1^ | [S32] |
| Na_2_-2,6-DHAQ | 70:22:8 | 1 M NaOTf  in G4 | 1.2 V/  1.0−3.0 V | 175/189 mAh g^−1^ @ 19 mA g^−1^ | 175 mAh g^-1^ (0.02 A g^-1^)  149 mAh g^-1^ (0.04 A g^-1^)  129 mAh g^-1^ (0.09 A g^-1^)  118 mAh g^-1^ (0.19 A g^-1^)  65 mAh g^-1^ (0.95A g^-1^)  28 mAh g^-1^ (1.89 A g^-1^) | 91%,50,  0.5 A g^-1^ | [S33] |
| AQDS | 7:2:1 | 1 M LiPF₆ in EC/DMC | 2.4V/  1.5-4V | 120/130 Ah g⁻¹  13 mA g⁻¹ | 130 mAh g^-1^ (0.013 A g^-1^)  110 mAh g^-1^ (0.026 A g^-1^)  55 mAh g^-1^ (0.13 A g^-1^) | 53%,100,  0.013 A g^-1^ | [S34] |
|   LiTCNQ | 6:3:1 | 1 M LiTFSI in DOL/DME with 1% LiNO_3_ | 3.1V/2.8-3.6V | 126/124 Ah g⁻¹  20 mA g⁻¹ | 125 mAh g^-1^ (0.04 A g^-1^)  105 mAh g^-1^ (0.06 A g^-1^)  100 mAh g^-1^ (0.08 A g^-1^)  97.5 mAh g^-1^ (0.1 A g^-1^)  90 mAh g^-1^ (0.15 A g^-1^) | 85%,100,  0.04 A g^-1^ | [S35] |
| Na_2_AQ_26_DS | 6:3:1 | 1 M LiTFSI in DOL/DME | 2.1V/  1-3.9V | 130/130 Ah g⁻¹  50 mA g⁻¹ | 138 mAh g^-1^ (0.05 A g^-1^)  120 mAh g^-1^ (0.1 A g^-1^)  100 mAh g^-1^ (0.2 A g^-1^)  80 mAh g^-1^ (0.5 A g^-1^)  60 mAh g^-1^ (1 A g^-1^)  40 mAh g^-1^ (2 A g^-1^) | 60%,2800,  0.5 A g^-1^ | [S36] |
|   AQ | 6:3:1 | 2 M LiTFSI in DOL/DME | 2.27V/  1.9-3.4V | 210/210 Ah g⁻¹  21 mA g⁻¹ | 257 mAh g^-1^ (0.051 A g^-1^)  130 mAh g^-1^ (1.12 A g^-1^) | 29.41%,1000,  1.12 A g^-1^ | [S37] |
|   DMAQ | 6:3:1 | 4 M LiTFSI in DOL/DME | 2.2V/  1.65-2.95V | 200/200 Ah g⁻¹  40 mA g⁻¹ | 200 mAh g^-1^ (0.04 A g^-1^)  139 mAh g^-1^ (0.16 A g^-1^)  70 mAh g^-1^ (2 A g^-1^) | 73%,80,  0.04 A g^-1^ | [S38] |
|   TAQ | 9:0:1 | 1 M LiPF_6_ in EC/DMC | 2.5V/  1.6-3.2V | 286/357.73 Ah g⁻¹  25 mA g⁻¹ | 297 mAh g^-1^ (0.04 A g^-1^)  240 mAh g^-1^ (0.1 A g^-1^)  106 mAh g^-1^ (0.5 A g^-1^)  105 mAh g^-1^ (0.75 A g^-1^) | 70%,2000,  1 A g^-1^ | [S39] |
|   NTCDA | 6:3:1 | LiTFSI in DOL/DME | 2.2V/  1.5-3.5V | 198/200 Ah g⁻¹  50 mA g⁻¹ | 140 mAh g^-1^ (0.05 A g^-1^)  135 mAh g^-1^ (0.1 A g^-1^)  125 mAh g^-1^ (0.2 A g^-1^)  120 mAh g^-1^ (0.5 A g^-1^)  80 mAh g^-1^ (1 A g^-1^) | 66%,1000,  0.1 A g^-1^ | [S40] |
|   P-NDI | 6:3:1 | 1 M LiTFSI in DOL/DME with 1% LiNO_3_ | 2.43V/  1.85-3V | 96/128 Ah g⁻¹  44.6 mA g⁻¹ | 60 mAh g^-1^ (0.05 A g^-1^)  45 mAh g^-1^ (0.1 A g^-1^)  42 mAh g^-1^ (0.2 A g^-1^)  28 mAh g^-1^ (0.5 A g^-1^)  20 mAh g^-1^ (1 A g^-1^)  13 mAh g^-1^ (2 A g^-1^) | 33%,400,  0.05 A g^-1^ | [S41] |
|   trans-2S | 6:3:1 | 1 M LiTFSI in DOL/DME with 1% LiNO_3_ | 2.55V/  1.85-3V | 116/122 Ah g⁻¹  44.6 mA g⁻¹ | 95 mAh g^-1^ (0.05 A g^-1^)  75 mAh g^-1^ (0.1 A g^-1^)  57 mAh g^-1^ (0.2 A g^-1^)  42 mAh g^-1^ (0.5 A g^-1^)  30 mAh g^-1^ (1 A g^-1^)  25 mAh g^-1^ (2 A g^-1^) | 37.5%,400,  0.05 A g^-1^ | [S41] |
|   cis-2S | 6:3:1 | 1 M LiTFSI in DOL/DME with 1% LiNO_3_ | 2.65V/  1.85-3V | 120/124 Ah g⁻¹  44.6 mA g⁻¹ | 105 mAh g^-1^ (0.05 A g^-1^)  90 mAh g^-1^ (0.1 A g^-1^)  75 mAh g^-1^ (0.2 A g^-1^)  60 mAh g^-1^ (0.5 A g^-1^)  45 mAh g^-1^ (1 A g^-1^)  25 mAh g^-1^ (2 A g^-1^) | 62.5%,400,  0.05 A g^-1^ | [S41] |
|   C4Q | 60:25:15 | 1 M LiPF_6_ in EC/DMC | 2.85V/  1.5-3.5V | 427/446 Ah g⁻¹  44.6 mA g⁻¹ | 446 mAh g^-1^ (0.044 A g^-1^)  350 mAh g^-1^ (0.088 A g^-1^)  300 mAh g^-1^ (0.132 A g^-1^)  250 mAh g^-1^ (0.22 A g^-1^)  170 mAh g^-1^ (0.44 A g^-1^) | 61%,100,  0.044 A g^-1^ | [S42] |
|   PTCDI/CB | 6:3:1 | 1 M LiPF_6_ in EC/DMC | 2.14V/  1.5-3.5V | 137/137 Ah g⁻¹  100 mA g⁻¹ | 137 mAh g^-1^ (0.1 A g^-1^)  133 mAh g^-1^ (0.2 A g^-1^)  130 mAh g^-1^ (0.5 A g^-1^)  124 mAh g^-1^ (1 A g^-1^)  110 mAh g^-1^ (2 A g^-1^) | 59%,600,  0.2 A g^-1^ | [S43] |
|   (COOLi)_2_-PDI | 55:35:10 | 1 M LiPF_6_ in EC/DMC | 2.12V/  1.5-3.5V | 101/103 Ah g⁻¹  17 mA g⁻¹ | 101 mAh g^-1^ (0.045 A g^-1^)  98 mAh g^-1^ (0.09 A g^-1^)  96 mAh g^-1^ (0.18 A g^-1^)  90 mAh g^-1^ (0.36 A g^-1^)  70 mAh g^-1^ (0.72 A g^-1^) | 84%,1000,  0.2 A g^-1^ | [S44] |
|   TDI | 5:4:1 | 1 M LiTFSI in DOL/DME | 2.63V/  1.8-3.2V | 56.7/64.3Ah g⁻¹  64.3 mA g⁻¹ | 60 mAh g^-1^ (0.013 A g^-1^)  45 mAh g^-1^ (0.032 A g^-1^)  37 mAh g^-1^ (0.0643 A g^-1^)  35 mAh g^-1^ (0.13 A g^-1^)  33 mAh g^-1^ (0.3215 A g^-1^) | 73.4%,100  0.064 A g^-1^ | [S45] |
|   PDI | 5:4:1 | 1 M LiTFSI in DOL/DME | 2.47V/  1.8-3.2V | 44.8/75.5Ah g⁻¹  75.5 mA g⁻¹ | 52 mAh g^-1^ (0.0151 A g^-1^)  30 mAh g^-1^ (0.0377 A g^-1^)  25 mAh g^-1^ (0.0755 A g^-1^)  22 mAh g^-1^ (0.151 A g^-1^)  18 mAh g^-1^ (0.377 A g^-1^) | 66%,100,  0.076 A g^-1^ | [S45] |
|   DQDPD@rGO | 5:4:1 | 1 M LiTFSI in DOL/DME | 2.2V/  1.0-3.5V | 505/519 mAh g⁻¹  200 mA g⁻¹ | 508 mAh g^-1^ (0.2 A g^-1^)  463 mAh g^-1^ (0.3 A g^-1^)  440 mAh g^-1^ (0.5 A g^-1^)  414 mAh g^-1^ (1 A g^-1^)  393 mAh g^-1^ (2 A g^-1^)  375 mAh g^-1^ (3 A g^-1^)  350 mAh g^-1^ (5 A g^-1^) | 82%,3000,  5 A g^-1^ | [S46] |
|   PTCDI-DAQ | 6:3:1 | 3 M LiTFSI in DOL/DME | 2.25V/  1.1-3.6V | 229/200 mAh g⁻¹  100 mA g⁻¹ | 146 mAh g^-1^ (0.1 A g^-1^)  94 mAh g^-1^ (3 A g^-1^) | 47%, 2000,  3 A g^-1^ | [S47] |
|   BAQB | 6:3:1 | 1 M LiTFSI in DOL/DME | 2.18V/  1.5-3V | 212/218 mAh g⁻¹  42.6 mA g⁻¹ | 218 mAh g^-1^ (0.043 A g^-1^)  125 mAh g^-1^ (2.15 A g^-1^） | 91%,100,  0.043 A g^-1^ | [S48] |

From the perspective of cycling performance, NDI-OLi outperforms all reported small-molecule organic cathode materials based on lithium salts. From the perspective of conjugation length, comparing with previously reported naphthalene diimide (NDI) small‑molecule derivatives can better highlight the advantages of our designed NDI‑OLi material. For example, P‑NDI possess similar conjugation lengths to NDI‑OLi (Table S3), but these non‑salt small molecules suffer from high solubility in organic electrolytes, leading to poor cycling performance. Although *trans‑2S* exhibits a slightly higher average discharge voltage (~2.6 V) than NDI‑OLi (~2.5 V) due to the electron‑withdrawing effect of the sulfur atom, it still faces dissolution issues, resulting in insufficient electrochemical stability. Li_2_NTCDI is structurally very similar to NDI‑OLi, but the ‑NLi group is prone to substitution during cycling, severely affecting its cycling reversibility.

In addition, NDI derivatives functionalized with carboxylic or sulfonic acid groups (e.g., Na_2_BNDI and Na_2_BESANDI) achieve commendable cycling stability, their ability to modulate the electronic structure and elevate the redox voltage is severely hindered by the presence of alkyl spacers (-CH_2_-). These saturated linkers effectively decouple the electron-withdrawing anionic groups from the NDI core, thereby diminishing the inductive effect on the redox-active centers. Consequently, Na_2_BNDI and Na_2_BESANDI deliver average discharge voltages of only 2.25 V and 2.3 V for LIBs, respectively, which are lower than that of NDI‑OLi. Furthermore, the high molecular weight of these electrochemically inactive carboxylate/sulfonate moieties significantly penalizes the theoretical specific capacity; for instance, Na₂BESANDI yields a capacity of only 113 mAh g^-1^. In summary, a systematic structural comparison reveals that NDI-OLi achieves an optimized balance of suppressed solubility, elevated voltage plateaus, robust cycling stability, and high specific capacity.

Supplementary References

1. M. J. F. et al. Gaussian, Inc, Wallingford CT. (2016).
2. Lu T, A comprehensive electron wavefunction analysis toolbox for chemists, Multiwfn. J. Chem. Phys. **161**(8), 082503 (2024). <https://doi.org/10.1063/5.0216272>
3. T. Lu, F. Chen, Multiwfn: a multifunctional wavefunction analyzer. J. Comput. Chem. **33**(5), 580–592 (2012). <https://doi.org/10.1002/jcc.22885>
4. W. Humphrey, A. Dalke, K. Schulten, VMD: Visual molecular dynamics. J. Mol. Graph. **14**(1), 33–38 (1996). <https://doi.org/10.1016/0263-7855(96)00018-5>
5. X. Han, C. Chang, L. Yuan, T. Sun, J. Sun, Aromatic carbonyl derivative polymers as high-performance Li-ion storage materials. Adv. Mater. **19**(12), 1616–1621 (2007). <https://doi.org/10.1002/adma.200602584>
6. G.S. Vadehra, R.P. Maloney, M.A. Garcia-Garibay, B. Dunn, Naphthalene diimide based materials with adjustable redox potentials: evaluation for organic lithium-ion batteries. Chem. Mater. **26**(24), 7151–7157 (2014). <https://doi.org/10.1021/cm503800r>
7. Y. Hanyu, I. Honma, Rechargeable quasi-solid state lithium battery with organic crystalline cathode. Sci. Rep. **2**, 453 (2012). <https://doi.org/10.1038/srep00453>
8. J. Li, H. Zhan, Y. Zhou, Synthesis and electrochemical properties of polypyrrole-coated poly(2, 5-dimercapto-1, 3, 4-thiadiazole). Electrochem. Commun. **5**(7), 555–560 (2003). <https://doi.org/10.1016/S1388-2481(03)00121-8>
9. J. Wang, A.E. Lakraychi, X. Liu, L. Sieuw, C. Morari et al., Conjugated sulfonamides as a class of organic lithium-ion positive electrodes. Nat. Mater. **20**(5), 665–673 (2021). <https://doi.org/10.1038/s41563-020-00869-1>
10. J. Hong, M. Lee, B. Lee, D.-H. Seo, C.B. Park et al., Biologically inspired pteridine redox centres for rechargeable batteries. Nat. Commun. **5**, 5335 (2014). <https://doi.org/10.1038/ncomms6335>
11. Deng, W, Shen, Y, Qian, J, Cao, Y, & Yang, H, A perylene diimide crystal with high capacity and stable cyclability for Na-ion batteries. ACS Appl. Mater. Interfaces **7**(38), 21095–21099 (2015). <https://doi.org/10.1021/acsami.5b04325>
12. Y. Liang, P. Zhang, S. Yang, Z. Tao, J. Chen, Fused heteroaromatic organic compounds for high-power electrodes of rechargeable lithium batteries. Adv. Energy Mater. **3**(5), 600–605 (2013). <https://doi.org/10.1002/aenm.201200947>
13. Z. Meng, K.A. Mirica, Two-dimensional d-π conjugated metal-organic framework based on hexahydroxytrinaphthylene. Nano Res. **14**(2), 369–375 (2021). <https://doi.org/10.1007/s12274-020-2874-x>
14. P. Chen, X. Su, C. Wang, G. Zhang, T. Zhang et al., Two-dimensional conjugated metal-organic frameworks with large pore apertures and high surface areas for NO_2_ selective chemiresistive sensing. Angew. Chem. Int. Ed. **62**(40), e202306224 (2023). <https://doi.org/10.1002/anie.202306224>
15. X. Sun, X. Yan, K. Song, T. Zhang, Z. Yang et al., A pyrazine-based 2D conductive metal-organic framework for efficient lithium storage. Chin. J. Chem. **41**(14), 1691–1696 (2023). <https://doi.org/10.1002/cjoc.202200819>
16. P. Chen, M. Liu, R. Li, X. Su, G. Xing et al., Multidentate macrocyclic salphen-based 2D conjugated metal–organic framework. Angew. Chem. Int. Ed. **64**(44), e202511048 (2025). <https://doi.org/10.1002/anie.202511048>
17. A.E. Lakraychi, K. Fahsi, L. Aymard, P. Poizot, F. Dolhem et al., Carboxylic and sulfonic N-substituted naphthalene diimide salts as highly stable non-polymeric organic electrodes for lithium batteries. Electrochem. Commun. **76**, 47–50 (2017). <https://doi.org/10.1016/j.elecom.2017.01.019>
18. D.J. Kim, S.H. Je, S. Sampath, J.W. Choi, A. Coskun, Effect of N-substitution in naphthalenediimides on the electrochemical performance of organic rechargeable batteries. RSC Adv. **2**(21), 7968–7970 (2012). <https://doi.org/10.1039/c2ra21239k>
19. Q. Zhao, J. Wang, Y. Lu, Y. Li, G. Liang et al., Oxocarbon salts for fast rechargeable batteries. Angew. Chem. Int. Ed. **55**(40), 12528–12532 (2016). <https://doi.org/10.1002/anie.201607194>
20. J. Xiang, C. Chang, M. Li, S. Wu, L. Yuan et al., A novel coordination polymer as positive electrode material for lithium ion battery. Cryst. Growth Des. **8**(1), 280–282 (2008). <https://doi.org/10.1021/cg070386q>
21. H. Chen, M. Armand, G. Demailly, F. Dolhem, P. Poizot et al., From biomass to a renewable LiXC_6_O_6_ organic electrode for sustainable Li-ion batteries. ChemSusChem **1**(4), 348–355 (2008). <https://doi.org/10.1002/cssc.200700161>
22. H. Chen, M. Armand, M. Courty, M. Jiang, C.P. Grey et al., Lithium salt of tetrahydroxybenzoquinone: toward the development of a sustainable Li-ion battery. J. Am. Chem. Soc. **131**(25), 8984–8988 (2009). <https://doi.org/10.1021/ja9024897>
23. A.-L. Barrès, J. Geng, G. Bonnard, S. Renault, S. Gottis et al., High-potential reversible Li deintercalation in a substituted tetrahydroxy-p-benzoquinone dilithium salt: an experimental and theoretical study. Chem. **18**(28), 8800–8812 (2012). <https://doi.org/10.1002/chem.201103820>
24. H. Chen, P. Poizot, F. Dolhem, N.I. Basir, O. Mentré et al., Electrochemical reactivity of lithium chloranilate vs Li and crystal structures of the hydrated phases. Electrochem. Solid-State Lett. **12**(5), A102 (2009). <https://doi.org/10.1149/1.3082038>
25. Q. Zhao, J. Wang, C. Chen, T. Ma, J. Chen, Nanostructured organic electrode materials grown on graphene with covalent-bond interaction for high-rate and ultra-long-life lithium-ion batteries. Nano Res. **10**(12), 4245–4255 (2017). <https://doi.org/10.1007/s12274-017-1580-9>
26. Q. Yu, Z. Yao, J. Shi, W. Tang, C. Wang et al., Electrochemically manipulating the redox state of 2, 2′, 5, 5′-tetrahydroxybiphenyl as a new organic Li-rich cathode for Li-ion batteries. Org. Electron. **81**, 105661 (2020). <https://doi.org/10.1016/j.orgel.2020.105661>
27. M. Yao, S. Umetani, H. Ando, T. Kiyobayashi, N. Takeichi et al., Rechargeable organic batteries using chloro-substituted naphthazarin derivatives as positive electrode materials. J. Mater. Sci. **52**(20), 12401–12408 (2017). <https://doi.org/10.1007/s10853-017-1368-z>
28. R.-H. Zeng, X.-P. Li, Y.-C. Qiu, W.-S. Li, J. Yi et al., Synthesis and properties of a lithium-organic coordination compound as lithium-inserted material for lithium ion batteries. Electrochem. Commun. **12**(9), 1253–1256 (2010). <https://doi.org/10.1016/j.elecom.2010.06.033>
29. M. Miroshnikov, K. Kato, G. Babu, K.P. Divya, L.M. Reddy Arava et al., A common tattoo chemical for energy storage: henna plant-derived naphthoquinone dimer as a green and sustainable cathode material for Li-ion batteries. RSC Adv. **8**(3), 1576–1582 (2018). <https://doi.org/10.1039/c7ra12357d>
30. M. Yao, N. Taguchi, H. Ando, N. Takeichi, T. Kiyobayashi, Improved gravimetric energy density and cycle life in organic lithium-ion batteries with naphthazarin-based electrode materials. Commun. Mater. **1**, 70 (2020). <https://doi.org/10.1038/s43246-020-00071-5>
31. X. Guo, P. Apostol, X. Zhou, J. Wang, X. Lin et al., Towards the 4 V-class n-type organic lithium-ion positive electrode materials: the case of conjugated triflimides and cyanamides. Energy Environ. Sci. **17**(1), 173–182 (2024). <https://doi.org/10.1039/d3ee02897f>
32. M. Lee, J. Hong, J. Lopez, Y. Sun, D. Feng et al., High-performance sodium–organic battery by realizing four-sodium storage in disodium rhodizonate. Nat. Energy **2**(11), 861–868 (2017). <https://doi.org/10.1038/s41560-017-0014-y>
33. L. Mu, Y. Lu, X. Wu, Y. Ding, Y.-S. Hu et al., Anthraquinone derivative as high-performance anode material for sodium-ion batteries using ether-based electrolytes. Green Energy Environ. **3**(1), 63–70 (2018). <https://doi.org/10.1016/j.gee.2017.09.002>
34. W. Wan, H. Lee, X. Yu, C. Wang, K.-W. Nam et al., Tuning the electrochemical performances of anthraquinone organic cathode materials for Li-ion batteries through the sulfonic sodium functional group. RSC Adv. **4**(38), 19878–19882 (2014). <https://doi.org/10.1039/c4ra01166j>
35. W. Deng, W. Shi, P. Li, N. Hu, S. Wang et al., A Li-contained air-stable cathode for high-performance all-organic lithium-ion batteries. Energy Storage Mater. **46**, 535–541 (2022). <https://doi.org/10.1016/j.ensm.2022.01.039>
36. W. Liu, W. Tang, X.-P. Zhang, Y. Hu, X. Wang et al., A polyanionic anthraquinone organic cathode for pure small-molecule organic Li-ion batteries. Int. J. Hydrog. Energy **46**(74), 36801–36810 (2021). <https://doi.org/10.1016/j.ijhydene.2021.08.203>
37. H. Zhang, R. Zhang, F. Ding, C. Shi, N. Zhao, Hydrogen bonding regulation enables indanthrone as a stable and high-rate cathode for lithium-ion batteries. Energy Storage Mater. **51**, 172–180 (2022). <https://doi.org/10.1016/j.ensm.2022.06.042>
38. J. Yang, Z. Wang, Y. Shi, P. Sun, Y. Xu, Poorly soluble 2, 6-dimethoxy-9, 10-anthraquinone cathode for lithium-ion batteries: the role of electrolyte concentration. ACS Appl. Mater. Interfaces **12**(6), 7179–7185 (2020). <https://doi.org/10.1021/acsami.9b19623>
39. T. Chen, H. Banda, J. Wang, J.J. Oppenheim, A. Franceschi et al., A layered organic cathode for high-energy, fast-charging, and long-lasting Li-ion batteries. ACS Cent. Sci. **10**(3), 569–578 (2024). <https://doi.org/10.1021/acscentsci.3c01478>
40. T. Cai, Y. Han, Q. Lan, F. Wang, J. Chu et al., Stable cycling of small molecular organic electrode materials enabled by high concentration electrolytes. Energy Storage Mater. **31**, 318–327 (2020). <https://doi.org/10.1016/j.ensm.2020.06.032>
41. B. Zhang, Y. Zhang, X. Yang, G. Li, S. Zhang et al., Isometric thionated naphthalene diimides as organic cathodes for high capacity lithium batteries. Chem. Mater. **32**(24), 10575–10583 (2020). <https://doi.org/10.1021/acs.chemmater.0c03661>
42. S. Zheng, H. Sun, B. Yan, J. Hu, W. Huang, High-capacity organic electrode material *Calix* [4] quinone/CMK-3 nanocomposite for lithium batteries. Sci. China Mater. **61**(10), 1285–1290 (2018). <https://doi.org/10.1007/s40843-018-9259-4>
43. D. Wu, F. Jing, X. Xi, L. Ma, D. Lu et al., An acid-pasting approach towards perylenetetracarboxylic diimide based lithium/sodium ion battery cathodes with high rate performances. J. Colloid Interface Sci. **538**, 597–604 (2019). <https://doi.org/10.1016/j.jcis.2018.11.085>
44. M. Veerababu, R. Kothandaraman, Rational functionalization of perylene diimide for stable capacity and long-term cycling performance for Li-ion batteries. Electrochim. Acta **232**, 244–253 (2017). <https://doi.org/10.1016/j.electacta.2017.02.152>
45. L. Li, J. Wang, M. Chen, Y. Chen, W. Xiao et al., The impact of vertical π-extension on redox mechanisms of aromatic diimide dyes. Chin. Chem. Lett. **30**(12), 2254–2258 (2019). <https://doi.org/10.1016/j.cclet.2019.05.040>
46. H. Peng, Y. Han, L. Zhang, Y. Geng, Z. Yu et al., Boosting lithium storage performance of small-molecule organic cathodes through synergistic molecular engineering and nanostructure design. Angew. Chem. Int. Ed. **64**(17), e202502088 (2025). <https://doi.org/10.1002/anie.202502088>
47. X. Wang, W. Tang, Y. Hu, W. Liu, Y. Yan et al., Insoluble small-molecule organic cathodes for highly efficient pure-organic Li-ion batteries. Green Chem. **23**(16), 6090–6100 (2021). <https://doi.org/10.1039/d1gc01927a>
48. J. Yang, H. Su, Z. Wang, P. Sun, Y. Xu, An insoluble anthraquinone dimer with near-plane structure as a cathode material for lithium-ion batteries. ChemSusChem **13**(9), 2436–2442 (2020). <https://doi.org/10.1002/cssc.201903227>
